# Supplementary material for: Effectiveness of a brief intervention and text-based booster in the emergency department to reduce harmful and hazardous alcohol use: A pragmatic randomized adaptive clinical trial in Moshi, Tanzania
Source: PLoS Med. 2025 Oct 27;22(10):e1004548. doi: 10.1371/journal.pmed.1004548 (PMC12578324; doi:10.1371/journal.pmed.1004548)
Supplement: S1 File — Fig A: Observed and predicted changes in binge drinking days using multiply imputed datasets, with and without statistical outliers. Table A: Enrollment criteria for patients that completed 3 months follow-up. Table B: Demographic and outcome characteristics for patients lost to follow-up at 3 months by study arm. Table C: Descriptive statistics of demographic variables and missingness in the main trial outcome at baseline or 3-months. Table D: Sensitivity analysis of primary and secondary outcomes: complete case-predicted means, between-group differences, confidence intervals, and p values (N = 310). Table E: Sensitivity analysis of primary outcome: complete case-predicted means, between-group differences, confidence intervals, and p values after excluding extreme outliers (based on 1.5 × IQR criterion) (n = 292). Table F: Sensitivity analysis of primary outcome: multiply-imputed predicted means, between-group differences, confidence intervals, and p values after excluding extreme outliers (based on 1.5 × IQR criterion) (N = 351). Table G: Observed and predicted median difference in binge drinking days and Wilcoxon rank-sum test results across analytic samples. Table H: Pooled coefficients from the zero-inflated model for the primary binge drinking days outcome, based on 100 multiply imputed datasets (n = 369). Table I: Pooled coefficients for the drinking days secondary outcome, based on 100 multiply imputed datasets, from models fit using negative binomial, zero-inflated negative binomial, and generalized Poisson distributions (n = 369). Table J: Bootstrapped within- and between-arm differences in predicted mean change in drinking days, based on 100 multiply imputed datasets, for models fit using negative binomial, zero-inflated negative binomial, and generalized Poisson distributions (n = 369). Table K: Observed and predicted median difference in drinking days and Wilcoxon rank-sum test results. Table L Pooled coefficients for the drinking amount secondary outco [file pmed.1004548.s001.docx]

# Supplementary File S1: Supplementary Tables and Figures

## Table A file S1. Enrollment criteria for patients that completed 3 months follow-up.

| **Characteristic** | **Overall**  **N = 364**^1^ | **Usual care**,  n = 123^1^ | **Intervention**,  n = 241^1^ |
| --- | --- | --- | --- |
| **Breathalyzer positive** | 9 / 362 (2.5%) | 4 / 123 (3.3%) | 5 / 239 (2.1%) |
| Missing | 2 | 0 | 2 |
| **AUDIT > 8** | 261 / 363 (72%) | 89 / 123 (72%) | 172 / 240 (72%) |
| Missing | 1 | 0 | 1 |
| **Self-reported alcohol use prior injury** | 259 / 364 (71%) | 91 / 123 (74%) | 168 / 241 (70%) |
| ^1^n / N (%)  AUDIT = Alcohol Use Disorder Identification Test | | | |

## Table B file S1. Demographic and outcome characteristics for patients lost to follow-up at 3 months by study arm

| **Characteristic** | **Intervention**, n = 48^1^ | **Usual care**, n = 23^1^ | ***p*-value**^2^ |
| --- | --- | --- | --- |
| **Self-reported sex** |  |  | 0.5 |
| Male | 47 (98%) | 22 (96%) |  |
| Female | 1 (2.1%) | 1 (4.3%) |  |
| **Age** | 32 (25, 41) | 30 (28, 49) | 0.4 |
| **Religion** |  |  | 0.12 |
| Christian | 42 (88%) | 16 (70%) |  |
| Muslim | 6 (13%) | 6 (26%) |  |
| Other | 0 (0%) | 1 (4.3%) |  |
| None | 0 (0%) | 0 (0%) |  |
| Refused/Don't know | 0 (0%) | 0 (0%) |  |
| **Years of education** | 7.00 (7.00, 11.00) | 7.00 (7.00, 9.00) | 0.10 |
| **Employment** |  |  | >0.9 |
| Unskilled-employment | 19 (40%) | 7 (33%) |  |
| Farmer (self-employed) | 12 (26%) | 7 (33%) |  |
| Skilled-employment | 10 (21%) | 4 (19%) |  |
| Other | 3 (6.4%) | 1 (4.8%) |  |
| Professional | 2 (4.3%) | 2 (9.5%) |  |
| Farmer (employee) | 1 (2.1%) | 0 (0%) |  |
| Unknown | 1 | 2 |  |
| **Tribe** |  |  | >0.9 |
| Chagga | 28 (58%) | 13 (57%) |  |
| Other | 15 (31%) | 7 (30%) |  |
| Pare | 5 (10%) | 3 (13%) |  |
| **Monthly personal income (Tz Shilling)** | 160,000 (100,000, 300,000) | 200,000 (100,000, 250,000) | 0.5 |
| Unknown | 10 | 2 |  |
| **Monthly total household income (Tz Shilling)** | 200,000 (150,000, 675,000) | 200,000 (140,000, 300,000) | 0.4 |
| Unknown | 18 | 4 |  |
| **Binge-drinking days** | 1 (0, 8) | 1 (0, 4) | >0.9 |
| Unknown | 1 | 1 |  |
| **Drinking days** | 6 (4, 12) | 6 (3, 12) | 0.9 |
| Unknown | 1 | 1 |  |
| **Drinking Amount** | 27 (11, 70) | 23 (9, 84) | 0.8 |
| Unknown | 1 | 1 |  |
| **DrInC score** | 13 (3, 31) | 11 (2, 20) | 0.2 |
| Unknown | 3 | 2 |  |
| **AUDIT score** | 14 (10, 19) | 14 (11, 20) | 0.8 |
| **PHQ9 score** | 2.0 (0.5, 8.0) | 2.0 (0.5, 5.0) | 0.9 |
| Unknown | 1 | 0 |  |
| ^1^n (%); Median (IQR) | | | |
| ^2^Fisher's exact test; Wilcoxon rank sum test  PHQ-9 = Patient Health Questionnaire-9  AUDIT = Alcohol Use Disorder Identification Test  DrInC = The Drinker Inventory of Consequences  *The intervention group is a pooled sample combining participants receiving standard and personalized text messages | | | |

## Table C file S1. Descriptive statistics of demographic variables and missingness in the main trial outcome at baseline or 3-months

| **Characteristic** | **Non-Missing**  N = 341^1^ | **Missing**  N = 28^1^ |
| --- | --- | --- |
| **Age** | 33 (27, 43) | 38 (28, 44) |
| **Self-reported sex** |  |  |
| Male | 319 (94%) | 27 (96%) |
| Female | 22 (6.5%) | 1 (3.6%) |
| **Tribe** |  |  |
| Chagga | 202 (59%) | 14 (50%) |
| Other | 105 (31%) | 11 (39%) |
| Pare | 34 (10.0%) | 3 (11%) |
| **Years of education** | 7.00 (7.00, 11.00) | 7.00 (7.00, 10.50) |
| *Missing* | 3 | 0 |
| **Employment** |  |  |
| Farmer (employee) | 4 (1.2%) | 0 (0%) |
| Farmer (self-employed) | 76 (23%) | 8 (29%) |
| Other | 27 (8.3%) | 0 (0%) |
| Professional | 37 (11%) | 5 (18%) |
| Skilled employment | 91 (28%) | 10 (36%) |
| Unskilled employment | 91 (28%) | 5 (18%) |
| *Missing* | 15 | 0 |
| **Monthly total household income (Tz Shilling)** | 300,000  (150,000, 550,000) | 300,000  (150,000, 450,000) |
| *Missing* | 88 | 6 |
| **Monthly personal income (Tz Shilling)** | 240,000  (100,000, 400,000) | 220,000  (150,000, 300,000) |
| *Missing* | 37 | 4 |

^1^Median (Q1, Q3); n (%)

# Sensitivity Analysis - Binge drinking days, main outcome

##

## Table D file S1. Sensitivity analysis of primary and secondary outcomes: complete case-predicted means, between-group differences, confidence intervals, and p values (N=310)

| **Variable** | **Arm** | **Predicted means** | | **Difference in means (95% CI)^1^** | **Difference in differences (95% CI)^2^** | ***p* value** |
| --- | --- | --- | --- | --- | --- | --- |
|  |  | **Baseline** | **3 Months** |  |  |  |
| Binge Drinking Days | Intervention | 3.5 | 0.1 | -3.4 (-4.8, -2.3) | -1.4 (-1.7, -1) | 0.0044 |
|  | Usual Care | 1.7 | 0.3 | -1.4 (-1.7, -1) |  |  |
| Drinking Days | Intervention | 8.3 | 0.8 | -7.5 (-8.5, -6.6) | -1 (-2.6, 0.8) | 0.0006 |
|  | Usual Care | 8.1 | 1.6 | -6.5 (-8, -5.3) |  |  |
| Drinking Amount | Intervention | 52.4 | 2.2 | -50.2 (-65.6, -38.1) | -11.4 (-28.9, 4.8) | 0.0033 |
|  | Usual Care | 43.3 | 4.5 | -38.8 (-48.7, -30.6) |  |  |
| DRINC | Intervention | 12.9 | 2.5 | -10.3 (-10.9, -9.7) | 0.2 (-1.1, 1.4) | 0.8922 |
|  | Usual Care | 13.4 | 2.8 | -10.5 (-11.6, -9.5) |  |  |
| AUDIT | Intervention | 13 | 4 | -9.1 (-9.4, -8.7) | -0.3 (-0.9, 0.2) | 0.6175 |
|  | Usual Care | 12.8 | 4.1 | -8.7 (-9.2, -8.3) |  |  |
| PHQ9 | Intervention | 3.1 | 4.1 | 1 (0.8, 1.2) | 0.2 (-0.1, 0.6) | 0.5882 |
|  | Usual Care | 3 | 3.7 | 0.7 (0.5, 1) |  |  |

¹,² Positive and negative values reflect the reduction or increase from predicted values at baseline compared to follow-up, respectively. *p* values represent the significance of the difference in differences;*The intervention group is a pooled sample combining participants receiving standard and personalized text messages

Table S4 displays primary and secondary outcome model results for patients that completed 3-month follow-up and had complete outcome data recorded. Figure 1 in the main text file displays the flow of participants in this trial and details the sample size at each stage. Figure 2 and Table 3 in the main text display the primary and secondary outcome model results using a multiple imputation approach (n=369).

## Table E file S1. Sensitivity analysis of primary outcome: complete case-predicted means, between-group differences, confidence intervals, and p values after excluding extreme outliers (based on 1.5×IQR criterion) (n=292)

| **Variable** | **Arm** | **Predicted Means** | | **Difference in Means (95% CI)1** | **Difference in Differences (95% CI)2** |
| --- | --- | --- | --- | --- | --- |
|  |  | **Baseline** | **3 Months** |  |  |
| Binge Drinking Days (complete cases, outliers removed) | Intervention | 1.7 | 0.1 | -1.6 (-2, -1.4) | -1 (-1.3, -0.7) |
|  | Usual Care | 1 | 0.3 | -0.7 (-0.8, -0.6) |  |

¹,² Positive and negative values reflect the reduction or increase from predicted values at baseline compared to follow-up, respectively.*The intervention group is a pooled sample combining participants receiving standard and personalized text messages

Table S5 presents the primary outcome model results for participants who completed the 3-month follow-up, had no missing data and had binge drinking values within 1.5 times the interquartile range (IQR) from the first and third quartiles (i.e., extreme outliers were excluded based on boxplot criteria). Figure 1 in the main manuscript illustrates the participant flow and sample sizes at each stage of the trial. Figure 2 and Table 3 in the main manuscript present the primary and secondary outcome model results using the multiply imputed analytic sample (n = 369).

## Table F file S1. Sensitivity analysis of primary outcome: multiply-imputed predicted means, between-group differences, confidence intervals, and p values after excluding extreme outliers (based on 1.5×IQR criterion) (N=351)

| **Variable** | **Arm** | **Predicted means** | | **Difference in Means (95% CI)1** | **Difference in Differences (95% CI)2** |
| --- | --- | --- | --- | --- | --- |
|  |  | **Baseline** | **3 Months** |  |  |
| Binge Drinking Days  (complete cases) | Intervention | 1.8 | 0.1 | -1.72 (-2.22, -1.37) | -0.92 (-1.45, -0.47) |
|  | Usual Care | 1.1 | 0.5 | -0.8 (-1.06, -0.58) |  |

¹,² Positive and negative values reflect the reduction or increase from predicted values at baseline compared to follow-up, respectively. *p* values represent the significance of the difference in differences*The intervention group is a pooled sample combining participants receiving standard and personalized text messages

Table S6 presents the primary outcome model results for participants who completed the 3-month follow-up and had binge drinking values within 1.5 times the interquartile range (IQR) from the first and third quartiles (i.e., extreme outliers were excluded based on boxplot criteria). Figure 1 in the main manuscript illustrates the participant flow and sample sizes at each stage of the trial. Figure 2 and Table 3 in the main manuscript present the primary and secondary outcome model results using the multiply imputed analytic sample (n = 369).

## Table G file S1. Observed and predicted median difference in binge drinking days and Wilcoxon rank-sum test results across analytic samples.

| **Sample** | **N** | **Median Difference (IQR)** | | **p value** |
| --- | --- | --- | --- | --- |
|  |  | **Usual care** | **Intervention** |  |
| Complete Cases | 310 | 0 (-1, 0) | 0 (-3, 0) | 0.0054 |
| Complete Cases - Removed Outliers | 292 | 0 (-1, 0) | 0 (-2, 0) | 0.0049 |
| Imputed data | 369 | 0 (-1, 0) | 0 (-3, 0) | 0.0148 |
| Imputed data - Removed Outliers | 351 | 0 (-1, 0) | 0 (-2, 0) | 0.0047 |

*The intervention group is a pooled sample combining participants receiving standard and personalized text messages

The imputed data analyses represent the primary results reported in the main text (N = 369, same sample as in Table 3 and Figure 2). Complete case analyses are presented for comparison (N = 310, corresponding to the sample used in Table S4). Additional analyses were conducted after removing statistical outliers, using interquartile range (IQR) thresholds, for both the imputed dataset (N = 351, same as Table S5) and the complete case dataset (N = 292, same as Table S6). For each sample, the table displays the median difference in binge drinking days along with interquartile ranges (IQR), and associated p-values from the Wilcoxon rank-sum test. Results were consistent across all analytic strategies, with the intervention group demonstrating greater reductions in binge drinking days compared to usual care.

## Table H file S1. Pooled coefficients from the zero-inflated model for the primary binge drinking days outcome, based on 100 multiply imputed datasets (n=369)

| **Characteristic** | **IRR** | **95% CI** | **p-value** |
| --- | --- | --- | --- |
| **Conditional Model** |  |  |  |
| Period 3 Months | 0.17 | 0.07, 0.43 | **<0.001** |
| Period 3 Months x Intervention | 0.25 | 0.09, 0.69 | **0.008** |
| **Zero-inflation** |  |  |  |
| Usual care | — | — |  |
| Intervention | 0.00 | 0.00, Inf | >0.9 |
| Abbreviation: IRR, Incidence Rate Ratio; CI = Confidence Interval;*The intervention group is a pooled sample combining participants receiving standard and personalized text messages | | | |

##

##


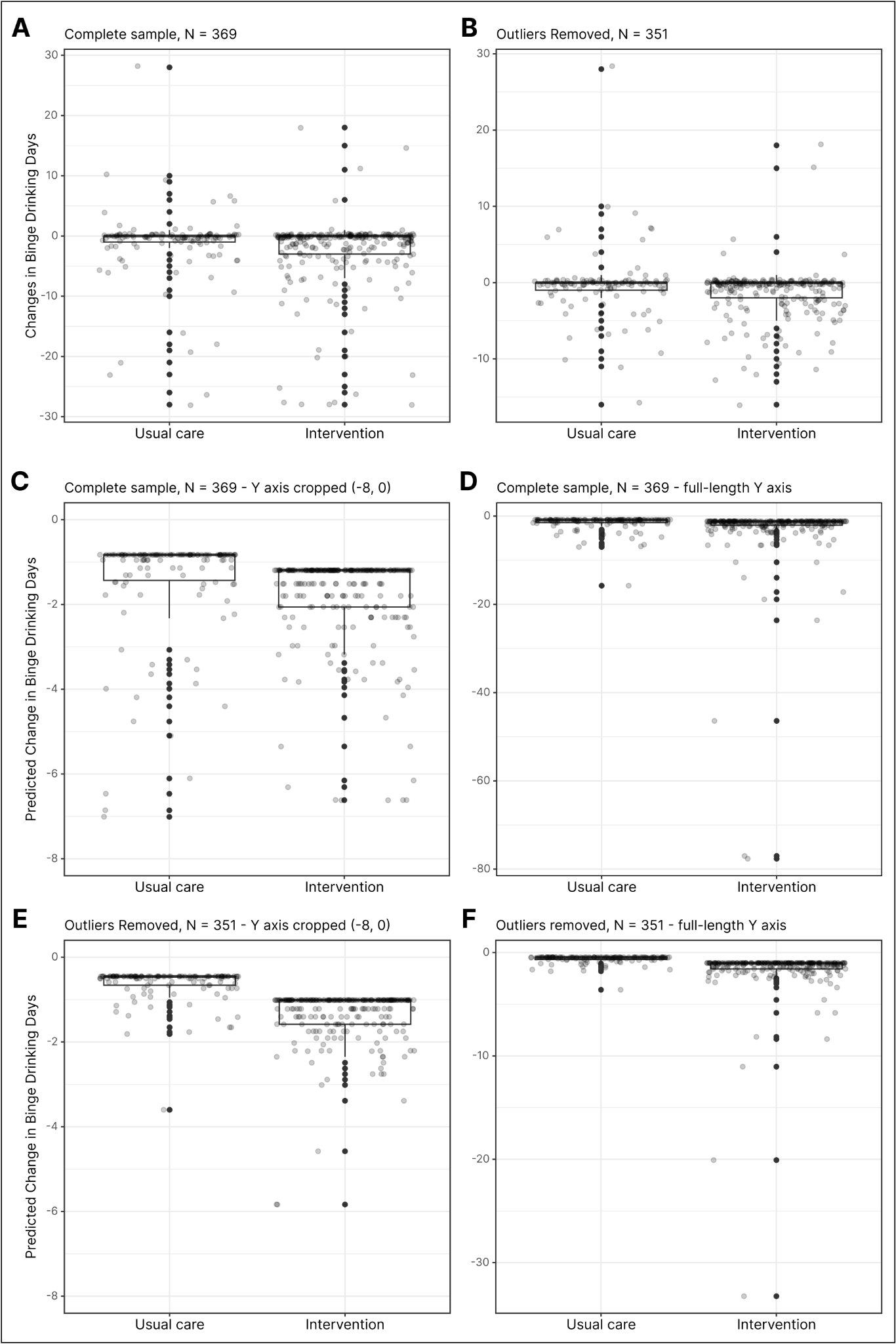


## Figure A file S1. Observed and predicted changes in binge drinking days using multiply imputed datasets, with and without statistical outliers.

Panels A and B display the *observed* change in binge drinking days. Panels C through F display the *predicted* change from the fitted models. Panels A, C, and D use the full imputed sample (N = 369), while Panels B, E, and F exclude statistical outliers (N = 351), defined using the interquartile range (IQR) method. Y-axis scaling varies to facilitate visual interpretation: Panels C and E use a cropped Y-axis (−8 to 0), while Panels D and F show the full Y-axis range.

# Sensitivity Analysis - Drinking Days, secondary drinking outcome

## Table I file S1- Pooled coefficients for the drinking days secondary outcome, based on 100 multiply imputed datasets, from models fit using negative binomial, zero-inflated negative binomial, and generalized Poisson distributions (n=369)

##

|  | **NBM** | | | **ZINBM** | | | **Gen. Poisson** | | |
| --- | --- | --- | --- | --- | --- | --- | --- | --- | --- |
| **Characteristic** | **IRR** | **95% CI** | **p-value** | **IRR** | **95% CI** | **p-value** | **IRR** | **95% CI** | **p-value** |
| **Conditional Model** |  |  |  |  |  |  |  |  |  |
| Period 3 Months | 0.22 | 0.16, 0.32 | <0.001 | 0.22 | 0.16, 0.32 | <0.001 | 0.20 | 0.15, 0.28 | <0.001 |
| Period 3 Months x Intervention | 0.49 | 0.33, 0.74 | <0.001 | 0.49 | 0.33, 0.74 | <0.001 | 0.61 | 0.41, 0.91 | 0.016 |
| **Zero-inflation** |  |  |  |  |  |  |  |  |  |
| Usual care |  |  |  | — | — |  |  |  |  |
| Intervention |  |  |  | 0.14 | 0.00, Inf | >0.9 |  |  |  |
| Empty values in the Zero-Inflation section indicate models where zero inflation was not included. Abbreviations: CI, Confidence Interval; NBM, Negative Binomial Model; ZINBM, Zero-Inflated Negative Binomial Model; Gen. Poisson, Generalized Poisson Model; IRR, Incidence Rate Ratio.*The intervention group is a pooled sample combining participants receiving standard and personalized text messages | | | | | | | | | |

## Table J file S1- Bootstrapped within- and between-arm differences in predicted mean change in drinking days, based on 100 multiply imputed datasets, for models fit using negative binomial, zero-inflated negative binomial, and generalized Poisson distributions (n=369)

##

| **Modelling Approach** | **Difference in Means (95% CI)1** | | **Difference in Differences (95% CI)2** |
| --- | --- | --- | --- |
|  | **Usual Care** | **Intervention** |  |
| Generalized Poisson | -6.25 (-6.58; -5.91) | -6.94 (-7.18; -6.7) | -0.69 (-1.04; -0.34) |
| Negative Binomial | -6.18 (-7.34; -5.14) | -7.19 (-8.09; -6.39) | -1 (-2.35; 0.36) |
| ZI - Negative Binomial | -6.18 (-7.34; -5.14) | -7.19 (-8.1; -6.39) | -1 (-2.33; 0.37) |

¹,² Positive and negative values reflect the reduction or increase from predicted values at baseline compared to follow-up, respectively. ZI, Zero-Inflated;*The intervention group is a pooled sample combining participants receiving standard and personalized text messages

## Table K file S1. Observed and predicted median difference in drinking days and Wilcoxon rank-sum test results.

| **Study Arm** | **Median Change (IQR)** | ***p value*** |
| --- | --- | --- |
| Intervention | -4 (-8.7, -2) | 0.0151 |
| Usual care | -3 (-7, -1) |  |

IQR, Interquartile range;*The intervention group is a pooled sample combining participants receiving standard and personalized text messages

# Sensitivity Analysis - Drinking Amount, secondary drinking outcome

## Table L file S1 - Pooled coefficients for the drinking amount secondary outcome, based on 100 multiply imputed datasets, from models fit using negative binomial, zero-inflated negative binomial, and generalized Poisson distributions

##

|  | **NBM** | | | **ZINBM** | | | **Gen. Poisson** | | |
| --- | --- | --- | --- | --- | --- | --- | --- | --- | --- |
| **Characteristic** | **IRR** | **95% CI** | **p-value** | **IRR** | **95% CI** | **p-value** | **IRR** | **95% CI** | **p-value** |
| **Conditional Model** |  |  |  |  |  |  |  |  |  |
| Period 3 Months | 0.15 | 0.09, 0.24 | <0.001 | 0.15 | 0.08, 0.26 | <0.001 | 0.14 | 0.10, 0.20 | <0.001 |
| Period 3 Months x Intervention | 0.37 | 0.21, 0.64 | <0.001 | 0.37 | 0.2, 0.67 | 0.001 | 0.53 | 0.36, 0.80 | 0.002 |
| **Zero-inflation** |  |  |  |  |  |  |  |  |  |
| Usual care |  |  |  | — | — |  |  |  |  |
| Intervention |  |  |  | 0.00 | Inf | 0.99 |  |  |  |
| Empty values in the Zero-Inflation section indicate models where zero inflation was not included. Abbreviations: CI, Confidence Interval; NBM, Negative Binomial Model; ZINBM, Zero-Inflated Negative Binomial Model; Gen. Poisson, Generalized Poisson Model; IRR, Incidence Rate Ratio.*The intervention group is a pooled sample combining participants receiving standard and personalized text messages | | | | | | | | | |

## Table M file S1. Bootstrapped within- and between-arm differences in predicted mean change in drinking amount, based on 100 multiply imputed datasets, for models fit using negative binomial, zero-inflated negative binomial, and generalized Poisson distributions (n=369)

| **Modelling Approach** | **Difference in Means (95% CI)1** | | **Difference in Differences (95% CI)2** |
| --- | --- | --- | --- |
|  | **Usual Care** | **Intervention** |  |
| Generalized Poisson | -36.95 (-38.85; -35.12) | -32.81 (-35.36; -30.32) | -4.14 (-7.13; -1.1) |
| Negative Binomial | -46.01 (-56.42; -37.45) | -34.86 (-41.79; -28.83) | -11.13 (-23.04; -0.29) |
| ZI-Negative Binomial | -46.01 (-56.53; -37.4) | -34.66 (-41.53; -28.54) | -11.32 (-23.04; -0.57) |

¹,² Positive and negative values reflect the reduction or increase from predicted values at baseline compared to follow-up, respectively. ZI, Zero-Inflated;*The intervention group is a pooled sample combining participants receiving standard and personalized text messages

##

##

## Table N file S1. Observed and predicted median difference in drinking amount and Wilcoxon rank-sum test results.

| **Study Arm** | **Median Change (IQR)** | ***p value*** |
| --- | --- | --- |
| Intervention | -18.7 (-42, -4.6) | 0.0091 |
| Usual care | -9 (-32, 0) |  |

IQR, Interquartile range.*The intervention group is a pooled sample combining participants receiving standard and personalized text messages
