# Supplementary material for: Effectiveness of a brief intervention and text-based booster in the emergency department to reduce harmful and hazardous alcohol use: A pragmatic randomized adaptive clinical trial in Moshi, Tanzania
Source: PLoS Med. 2025 Oct 27;22(10):e1004548. doi: 10.1371/journal.pmed.1004548 (PMC12578324; doi:10.1371/journal.pmed.1004548)
Supplement: S2 File — (PDF) [file pmed.1004548.s002.pdf]

PRACT: A Pragmatic Randomized Adaptive Clinical Trial to Investigate Controlling Alcohol-related Harms in a Low-Income Setting; Emergency Department Brief Interventions in Tanzania

This title should include, where possible, information on the participants, condition being evaluated, and intervention(s) studied.

Unique Protocol Identification Number: Pro00103724

National Clinical Trial (NCT) Identified Number: NCT04535011

Principal Investigator: Catherine A. Staton

Sponsor: NIAAA

"Sponsor" indicates an institution, foundation, or individual who takes responsibility for and initiates a clinical investigation; often times this is the university with which the Principal Investigator is affiliated.

Grant Title: PRACT: A Pragmatic Randomized Adaptive Clinical Trial to Investigate Controlling Alcohol-related Harms in a Low-Income Setting; Emergency Department Brief Interventions in Tanzania

Grant Number: R01AA027512

Funded by: NIAAA

Version Number: v.1.1

16 June 2023

All versions should have a version number and a date. Use an international date format (e.g.,YYYY-MM-DD [2017-12-21] or write out the month (e.g., 21 December 2017).
For the initial submission of a protocol to the IRB, indicate "Not applicable; this is the first version of the protocol." in the table below. For any subsequent amendment being submitted to the IRB, add details of the specific changes that are being implemented in the amendment. Please note that Section 10.4 is a high-level summary of <u>all</u> formal protocol versions/amendments.

Changes in the current version:

1. Due to a slower enrollment rate than initially anticipated and COVID-related delays to study start, the previous three-phase study design is not feasible within the planned five-year study period. As such, we intend to modify our plan to be completed in **two phases**. The amended second phase will:
   - compare the brief intervention ("PPKAY") alone with PPKAY + standard SMS booster and PPKAY + personalized SMS booster,
   - reduce the overall maximum sample size to 864 participants, and
   - still manage to achieve the original aims of the project.

2. In addition, in support of Objective #3, **qualitative interview questions** have been added, to be asked of a subset of enrolled participants at the 24-month follow-up visit.

3. Finally, as the original protocol considered the **24-month follow-up** visit as an uncertain possibility, we have clarified that the 24-month follow-up visit is a planned part of the study activities.

Table of Contents

Statement of Compliance 5
Investigator's Signature 5
1 Protocol Summary 7
1.1 Synopsis 7
1.2 Schema 8
1.3 Schedule of Activities 9
2 Introduction 10
2.1 Study Rationale 10
2.2 Background 10
2.3 Risk/Benefit Assessment 11
2.3.1 Known Potential Risks 11
2.3.2 Known Potential Benefits 12
2.3.3 Assessment of Potential Risks and Benefits 12
3 Objectives and Endpoints 13
4 Study Design 14
4.1 Overall Design 14
4.2 Scientific Rationale for Study Design 16
4.3 Justification for Intervention 17
4.4 End-of-Study Definition 19
5 Study Population 20
5.1 Inclusion Criteria 20
5.2 Exclusion Criteria 20
5.3 Lifestyle Considerations 20
5.4 Screen Failures 20
5.5 Strategies for Recruitment and Retention 21
6 Study Intervention(s) or Experimental Manipulation(s) 24
6.1 Study Intervention(s) or Experimental Manipulation(s) Administration 24
6.1.1 Study Intervention or Experimental Manipulation Description 24
6.1.2 Administration and/or Dosing 24
6.2 Fidelity 25
6.2.1 INTERVENTIONIST TRAINING AND TRACKING 25
6.3 Measures to Minimize Bias: Randomization and Blinding 26
6.4 Study Intervention/Experimental Manipulation Adherence 26
6.5 Concomitant Therapy 27
6.5.1 Rescue Therapy 27
7 Study Intervention/Experimental Manipulation Discontinuation and Participant Discontinuation/Withdrawal 28
7.1 Discontinuation of Study Intervention/Experimental Manipulation 28
7.2 Participant Discontinuation/Withdrawal from the Study 28
7.3 Lost to Follow-Up 29
8 Study Assessments and Procedures 30
8.1 Endpoint and Other Non-Safety Assessments 30
8.2 Safety Assessments 32
8.3 Adverse Events and Serious Adverse Events 33
8.3.1 Definition of Adverse Events 33
8.3.2 Definition of Serious Adverse Events 33

8.3.3 Classification of an Adverse Event 33
8.3.4 Time Period and Frequency for Event Assessment and Follow-Up 33
8.3.5 Adverse Event Reporting 33
8.3.6 Serious Adverse Event Reporting 34
8.3.7 Reporting Events to Participants 34
8.3.8 Events of Special Interest 34
8.3.9 Reporting of Pregnancy 34
8.4 Unanticipated Problems 34
8.4.1 Definition of Unanticipated Problems 34
8.4.2 Unanticipated Problems Reporting 34
8.4.3 Reporting Unanticipated Problems to Participants 35
9 Statistical Considerations 36
9.1 Statistical Hypotheses 36
9.2 Sample Size Determination 36
9.3 Populations for Analyses 37
9.4 Statistical Analyses 38
9.4.1 General Approach 38
9.4.2 Analysis of the Primary Endpoint(s) 38
9.4.5 Baseline Descriptive Statistics 38
9.4.6 Planned Interim Analyses 38
9.4.7 Sub-Group Analyses 39
9.4.8 Tabulation of Individual Participant Data 39
10 Supporting Documentation and Operational Considerations 40
10.1 Regulatory, Ethical, and Study Oversight Considerations 40
10.1.1 Informed Consent Process 40
10.1.2 Study Discontinuation and Closure 40
10.1.3 Confidentiality and Privacy 41
10.1.4 Future Use of Stored Specimens and Data 41
10.1.5 Key Roles and Study Governance 41
10.1.6 Safety Oversight 41
10.1.8 Quality Assurance and Quality Control 41
10.1.9 Data Handling and Record Keeping 42
10.1.10 Protocol Deviations 42
10.1.11 Publication and Data Sharing Policy 42
10.1.12 Conflict of Interest Policy 42
10.2 Additional Considerations 43
10.3 Abbreviations and Special Terms 43
10.4 Protocol Amendment History 43
11 References 45
References 45

STATEMENT OF COMPLIANCE

Provide a statement that the trial will be conducted in compliance with the protocol, International Council on Harmonisation Good Clinical Practice (ICH GCP) and applicable state, local and federal regulatory requirements. Each engaged institution must have a current Federal-Wide Assurance (FWA) issued by the Office for Human Research Protections (OHRP) and must provide this protocol and the associated informed consent documents and recruitment materials for review and approval by an appropriate Institutional Review Board (IRB) or Ethics Committee (EC) registered with OHRP. Any amendments to the protocol or consent materials must also be approved before implementation. Select one of the two statements below. If the study is an ***intramural*** *NIH study, use the second statement below:*

1. The trial will be carried out in accordance with International Council on Harmonisation Good Clinical Practice (ICH GCP) and the following:

    - United States (US) Code of Federal Regulations (CFR) applicable to clinical studies (45 CFR Part 46, 21 CFR Part 50, 21 CFR Part 56, 21 CFR Part 312, and/or 21 CFR Part 812).

    National Institutes of Health (NIH)-funded investigators and clinical trial site staff who are responsible for the conduct, management, or oversight of NIH-funded clinical trials have completed Human Subjects Protection and ICH GCP Training.
    OR
2. The trial will be conducted in accordance with International Council on Harmonisation Good Clinical Practice (ICH GCP), applicable United States (US) Code of Federal Regulations (CFR), and the [specify NIH Institute or Center (IC) [Terms and Conditions of Award. The Principal Investigator will assure that no deviation from, or changes to the protocol will take place without prior agreement from the funding agency and documented approval from the Institutional Review Board (IRB), and the Investigational New Drug (IND) or Investigational Device Exemption (IDE) sponsor, if applicable, except where necessary to eliminate an immediate hazard(s) to the trial participants. All personnel involved in the conduct of this study have completed Human Subjects Protection and ICH GCP Training.
    For either option above, the following paragraph would be included:
    The protocol, informed consent form(s), recruitment materials, and all participant materials will be submitted to the IRB for review and approval. Approval of both the protocol and the consent form(s) must be obtained before any participant is consented. Any amendment to the protocol will require review and approval by the IRB before the changes are implemented to the study. All changes to the consent form(s) will be IRB approved; a determination will be made regarding whether a new consent needs to be obtained from participants who provided consent, using a previously approved consent form.

INVESTIGATOR'S SIGNATURE

Principal Investigator or Clinical Site Investigator:

Signed: [signature] Date: 16 June 2023

Name*: Catherine A. Staton
Title*: Associate Professor

Investigator Contact Information:

Affiliation*: Duke University
Address: 2301 Erwin Road, DUMC Box 3096
Telephone: 919-681-2981
Email: catherine.staton@duke.edu

For multi-site studies, the protocol should be signed by the clinical site investigator who is responsible for the day to day study implementation at his/her specific clinical site:

Signed: [signature] Date: 16 June 2023

Name: Blandina T. Mmbaga
Title: Director
Affiliation: Kilimanjaro Clinical Research Institute

1 PROTOCOL SUMMARY

1.1 SYNOPSIS

**Title:**	PRACT: A Pragmatic Randomized Adaptive Clinical Trial to Investigate Controlling Alcohol-related Harms in a Low-Income Setting; Emergency Department Brief Interventions in Tanzania
**Grant Number:**	
**Study Description:**	
**Objectives*:**	Primary Objective: To evaluate the effectiveness of PPKAY for a patient-level intervention in this high-risk population with a PRACT<br>Secondary Objectives:
**Endpoints*:**	Primary Endpoint: Number of binge drinking days<br>Secondary Endpoints: Quantity and frequency of alcohol use, Alcohol-related harms measured by the Drinker Inventory of Consequences (DrInC), Alcohol use disorder (AUDIT score), Depression (PHQ-9)
**Study Population:**	**Sample size**: 864 maximum<br>**Gender**: Approximately 24% women, 76% men,<br>**Age**: ≥ 18 years<br>**Characteristics**:<br>• Acute injury patients<br>• Seeking emergency treatment at KCMC in Moshi, Tanzania<br>• Alcohol-related injury **or** suspected alcohol use disorder<br>• Fluent in Swahili<br>• Have access to SMS-capable mobile device
**Phase* or Stage:**	II/III
**Description of Sites/Facilities Enrolling Participants:**	Kilimanjaro Christian Medical Center (KCMC), established in 1971 as a Zonal Referral Consult Hospital in Moshi, Tanzania. KCMC has a 450-bed hospital, outpatient departments that attend to 500+ patients daily, administrative offices, dormitories, and housing for international visitors.

**Description of Study Intervention/Experimental Manipulation:**	• Usual care<br>• Brief intervention<br>• Brief intervention + standard text booster<br>• Brief intervention + personalized text booster
**Study Duration*:**	9-57 months
**Participant Duration:**	Maximum of 24 months

1.2 SCHEMA

PRACT Study Design

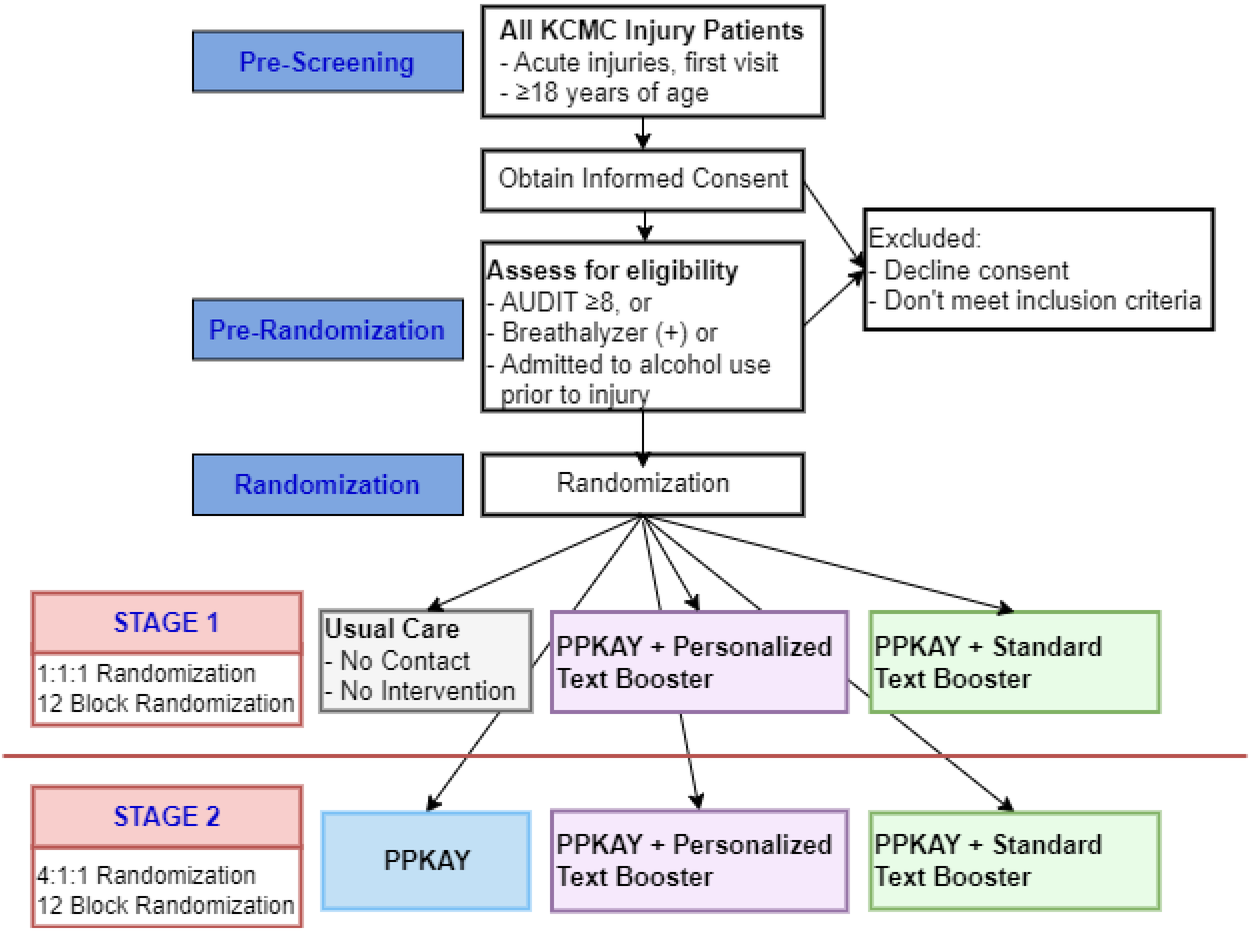

PRACT Participant Event Schema

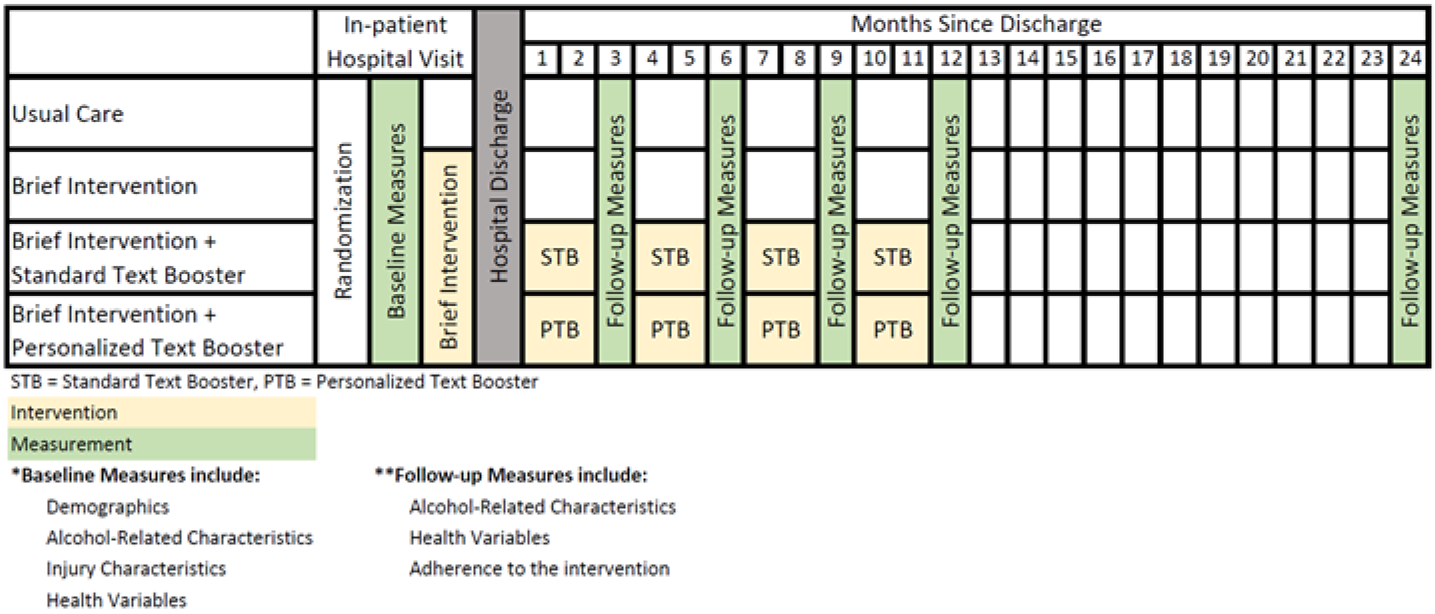

1.3 SCHEDULE OF ACTIVITIES

Baseline Measures (in hospital) Include:
Demographics
Alcohol-Related Characteristics
Injury Characteristics
Health Variables

Follow-up Measures (3, 6, 9, 12, 24 months post-discharge) Include:
Alcohol-Related Characteristics
Health Variables
Adherence to the intervention

At 24-month follow-up visit only, we will conduct qualitative interviews with a sub-sample of enrolled participants to further understand potential mediators or moderators of the intervention.

Follow-up Research Visits at 3, 6, 9, 12, and 24 months post-discharge will be allowed +/- 4 weeks of scheduled visit.

Text messages will be sent from 0 to 12 months post-discharge.

2 INTRODUCTION

2.1 STUDY RATIONALE

Alcohol use is rapidly increasing in low- and middle-income countries, where it is inexpensive, readily available, poorly regulated, and there are few resources devoted to promoting safe alcohol use. Like many other limited resource settings, there are no treatment facilities or addiction practitioners in the Kilimanjaro region of Tanzania. For many in this setting, they only seek care after an emergency like an acute injury. From our preliminary work, we have seen that 30% of injury patients presenting for care in the Emergency Department report excess alcohol use and are at risk of a repeat injury. A Brief Intervention based on a motivational interviewing framework has been shown to reduce alcohol use and alcohol-related harms. We have translated and adapted a Brief Intervention for alcohol to the Tanzanian context and Swahili language called “Punguza Pombe Kwa Afya Yako (PPKAY)/ Reduce Alcohol for Your Health.” This project will evaluate this intervention in injury patients presenting for care at the Kilimanjaro Christian Medical Center in Moshi, Tanzania. By using innovative adaptive clinical trial methods, we will expedite the development of the most effective way to integrate this intervention into clinical care. By the end of this project, we will have identified the most effective brief intervention components and be able to characterize the intervention’s effect overall. Additionally, we will standardize adaptive trial methods to revolutionize the science of clinical trials for behavioral sciences in low-resource settings.

2.2 BACKGROUND

Alcohol-related health consequences, in Africa and specifically Tanzania, are increasing; especially where there are severely limited treatment options. Globally, alcohol causes over 3.3 million deaths annually.[1] Concerningly, alcohol use has increased over 32% in the last 20 years worldwide, becoming the 5th leading risk factor for death or disability.[2] Nearly one-third of all deaths attributed to alcohol globally are caused by injuries.[1] More specifically, in the Africa region, drinking patterns are the second worst worldwide with high rates of binge drinking and alcohol dependence.[2,3] East Africa, and specifically Tanzania has a high rate of Alcohol Use Disorders (AUD) due to an increase in availability of alcohol.[4] In Tanzania, 11.5% of men and 6.8% of the overall population were estimated to have an AUD, with 33.4% of males having heavy episodic drinking.[1] Within Tanzania, the Kilimanjaro region’s scarce literature has shown very high rates of AUD, especially among high-risk groups.[5,6] Both chronic and heavy episodic alcohol use have been associated with many high-risk behaviors: crime, aggressive driving, HIV risk behaviors, interpersonal violence, unintentional injuries, and self-inflicted injury.[1,7,8,9,10,11,12] As such, in Tanzania, and specifically the Kilimanjaro region, harmful alcohol use is common and escalating, and there are limited treatment options, personnel, training, and capacity to curb this burden.[13] Further research, including the current proposal, is needed to guide implementation priorities in this region. Our Solution to Tanzania’s Treatment Gap: An Emergency Department -based Brief Intervention Scientific

Premise Aim 1a: A brief intervention for alcohol reduces alcohol -related harms compared to usual care. In high-income settings, a brief intervention (BI) administered in an Emergency Department (ED) setting for patients with AUDs cost-effectively reduces a patient's alcohol intake and re-injury rate up to 3 years post-intervention.[14,15,16,17,18] Numerous meta-analyses have documented this effect for both primary care and ED settings.[19,20,21,22,23] While a recent pragmatic trial, the SIPS-ED trial, showed minimal to no reduction in alcohol use,[24] many suggest this was due to poor intervention fidelity.[25] While the premise of BIs is well founded, there is limited literature about this intervention's impact in low-income country settings, nor the optimal length, frequency and content of interventions.[19,20] There have been numerous studies in Africa on BIs, but the vast majority have occurred in South Africa and in non-ED settings.[262728293031323334] Sorsdahl et al. conducted a feasibility and clinical trial of a BI versus other intervention methods (problem solving therapy) in the ED setting in Cape Town, South Africa.[353637] Yet, these studies are set in a far more resourced environment in South Africa and require more advanced counseling capacity than is a vailable in Tanzania's setting. In low- and middle-income countries (LMICs), such as Tanzania, politicians, planners, and healthcare practitioners are more focused on those with alcohol dependence rather than the more highly functioning harmful and hazardous drinkers, who suffer most of the alcohol -related consequences.3,38 Accordingly, treatment in LMICs, if there is any, has been focused on long-term residential treatment centers with limited efficacy.[383940] But Benegal et al. suggest a step-wise implementation of alcohol interventions, starting with opportunistic screening and brief advice and progressing to other, more complex treatment options.[38] However, the optimal format of such interventions is not evident. For example, it is not clear whether the additional complexity of using personalized boosters has added value in this limited resource context. Our team has created and piloted "Punguza Pombe Kwa Afya Yako" (PPKAY)/ "Reduce Alcohol for Your Health"; a brief intervention for the KCMC ED. PPKAY was based upon NIAAA BI suggested standards, adapted to Swahili and the Tanzanian environment, and piloted during prior NIH-funded work in Tanzania (See Methods and Intervention Development).[41] This is a one-time, 15-minute, nurse-led intervention based on motivational interviewing principles, discusses safe drinking behaviors, and negotiates change in alcohol use.[4243] Our trial compares PPKAY to usual care of no alcohol harm prevention education.

2.3 RISK/BENEFIT ASSESSMENT

2.3.1 KNOWN POTENTIAL RISKS

Potential risks to participants in our adaptive clinical trial include potential psychological stress when discussing alcohol use during the trial, security of information about personal alcohol use and the potential social and medico-legal risk of collecting information about personal alcohol use amongst acutely injured patients. We have performed similar alcohol-related surveys and alcohol-related testing for this injury population in the ED without any of our >800 participants reporting problems related to any of these potential risks. As such, we anticipate the probability of any of these risks to be low. Even still, all information collected from these surveys will be kept in a confidential manner separate from any identifying information in order to ensure patient confidentiality and avoid these potential risks.

2.3.2 KNOWN POTENTIAL BENEFITS

Proposed Benefits: Those involved in all but one of the intervention arms of the trial will receive our brief intervention to reduce high-risk alcohol use. There are no current alternative treatment options for alcohol use in Moshi, Tanzania, so these services are not otherwise available. This knowledge will allow us to guide appropriate treatment of alcohol use and high-risk alcohol use in Tanzania. Therefore, these studies have limited risk to participants, have the potential of improvement of their alcohol use disorder and will have great importance in terms of the knowledge gained for the Tanzanian community.

2.3.3 ASSESSMENT OF POTENTIAL RISKS AND BENEFITS

Given most of the risk in this study stems from potential loss of confidentiality, especially related to alcohol use information, we will maintain strict confidentiality for our study. Study nurses will complete all pre-randomization, post randomization procedures as well as the intervention and follow-up in a quiet secure location, and all study documents will have only a study identification number without other identifying information on them. Enrollment logs, which will have personal identifying information and study identification numbers on them, will remain only in paper form in a locked room in a locked drawer at KCMC and will not be placed in our online data repository. Should we identify patients with severe alcohol use disorders or patients with severe mental health issues, we will do our best to ensure the safety of these patients. There are no treatment options for those with severe alcohol use disorder available in Tanzania. For patients with severe alcohol use disorders who require admission to the hospital and stabilization, we will refer them to Kilimanjaro Christian Medical Center Emergency Department for evaluation and potential admission to the hospital for stabilization. If we encounter patients with severe mental health issues, such as severe depression or suicidal thoughts or behaviors, we will refer and facilitate urgent follow-up with a local mental health provider in Moshi.

3 OBJECTIVES AND ENDPOINTS

OBJECTIVES	ENDPOINTS	
Primary		
To determine if "Punguza Pombe Kwa Afya Yako" (PPKAY) reduces alcohol use and alcohol-related harms among injury patients in Moshi, Tanzania.	Alcohol-related harms (DrInC)<br>Quantity of alcohol use<br>Frequency of alcohol use<br>Number of binge drinking episodes<br>Depression (PHQ-9)	
Secondary		
To determine if a mobile health-based booster improves PPKAY effectiveness in our injury patient population in Moshi, Tanzania.	Alcohol-related harms (DrInC)<br>Quantity of alcohol use<br>Frequency of alcohol use<br>Number of binge drinking episodes<br>Depression (PHQ-9)	
N/A		

4 STUDY DESIGN

4.1 OVERALL DESIGN

Aim 1: To determine if our brief intervention, PPKAY, reduces alcohol use and alcohol-related harms among injury patients in Moshi, Tanzania. Aim 1 will occur during phase 1 of this two-phase PRACT. We will evaluate whether PPKAY or PPKAY with booster will reduce the quantity or frequency of alcohol use, the number of binge drinking events or the number of alcohol-related consequences based on the Drinker Inventory of Consequences (DrInC) compared to usual care.

Aim 2: To determine if a booster improves the effectiveness of the PPKAY in our injury patient population in Moshi, Tanzania. Next, we will evaluate if a standard or a personalized (Phase 2) text booster will improve the effectiveness of PPKAY. Given Aim 1 and 2 use similar methods, we will describe them together below. For each Aim, we will also conduct a mediator/moderator analysis.
Phase: II/III

Trial design: pragmatic, randomized, adaptive clinical trial.

Design: Phase 1 of our PRACT is a three-arm trial comparing usual care to PPKAY with personalized booster or PPKAY with standard booster using a 1:1:1 allocation with a 12-block randomization. Adaptation to Phase 2 will occur when we have reached statistical significance of a difference in the number of binge drinking days in our intervention groups over control groups. Phase 2 of our PRACT adds PPKAY alone and maintains our PPKAY with personalized booster and PPKAY with standard booster at a 4:1:1 allocation with a 12-block randomization. Completion of our trial will occur when 324 participants are enrolled in Phase 2

Randomization: Prior to study initiation, Phase 1 potential study identification numbers will be randomized by computer software into 3 groups (Usual Care, PPKAY with Personalized Booster or PPKAY with Standard Booster) in a 12-block randomization at a 1:1:1 allocation. Potential Phase 2 study identification numbers will be randomized by computer software into 3 groups (PPKAY alone, PPKAY with Personalized Booster or PPKAY with Standard Booster) in a 12-block randomization at a 4:1:1 allocation. We will prepare randomization envelopes for 110% of maximum enrollment numbers per Phase (See Sample Size). Block randomization will maintain balance among the study arms given the potential impact of time-dependent events impacting alcohol use (e.g., seasons, holidays, payday, etc.). Enrollment packets of the same size and thickness will be placed in opaque envelopes, sealed and locked in a drawer at the study site in consecutive order by study identification number. In each envelope there will be one paper delineating the random group assignment for each participant. To ensure reproducibility in any resourced context, we will maintain paper randomization packets and data collection sheets.

Blinding and Limiting Biases: In an adaptive clinical trial, investigator level blinding is key to limiting biases during the adaptations and analysis, while blinding at the nursing level will limit the bias at the patient level. Our unblinded analysis leader, Dr. Vissoci, will conduct the analysis and bring the data to the DSMB for them to determine if we have reached a priori established adaptation decision points. Our DSMB will evaluate our preliminary analysis data, oversee the sample size re-estimation based on up-to-date trial effect size data and decide if and when adaptations are implemented, as investigators and research personnel will remain blinded whenever possible. Since Dr. Vissoci will manage the data management, the data quality improvement processes and present the data to the DSMB for decision-making, he will remain unblinded. To ensure rigorous data, intensive quality assurance processes and audits are planned, which blinded investigators cannot conduct. Thus, Dr. Vissoci must remain unblinded. Dr. Vissoci will also have no role in the management of the clinical trial in Tanzania to ensure we limit any potential biases. At the Research Nurse level, we have established roles so that outcomes assessors are blinded to a patient's allocation.

Retention: Once patients are enrolled, randomized, and undergo the initial processes associated with their intervention arm, patient follow-up will start. Follow-up will be at 3, 6, 9, 12, and 24 months (all arms). We will obtain three phone numbers for patients including family members or friends in order to ensure we can contact patients after discharge from the hospital and initial study processes. We will obtain outcome data through self-report from patients over the phone or in-person based on patient preference. We have also budgeted funds in order to reimburse patients for their time and phone expenses to conduct these outcome interviews.

Administrative Site/Data Coordinating Site: Duke University Medical Center
Participating Site (the only site at which patients will be recruited): Kilimanjaro Christian Medical Center

Trial Arms

Usual Care	PPKAY	PPKAY with Standard Booster	PPKAY with Personalized Booster
No prevention or educational information verbally or written coinciding with current usual care	One-time, 15-minute, nurse-led motivational interview discussing safe drinking behaviors, and negotiating change in alcohol use	One-time, 15-minute, nurse-led motivational interview discussing safe drinking behaviors, and negotiating change in alcohol use Weekly standard text booster until final follow-up (e.g., "Reducing your alcohol intake to less than 4 drinks per day reduces your risk of alcohol-related consequences")	One-time, 15-minute, nurse-led motivational interview discussing safe drinking behaviors, and negotiating change in alcohol use Weekly personalized text booster until final follow-up (e.g., "Remember to reduce your alcohol less than 4 drinks to achieve your goal of… [being a better husband].")

PPKAY=Punguza Pombe Kwa Afya Yako ("Reduce Alcohol For Your Health")
**Study periods vary depending on effect size, but minimum follow up 12 months and, if time permits, a maximum follow-up time 24 months

Interim Analyses/Adaptations:
In Stage 1, up to 3 interim analyses will be conducted after the first batch of patients complete 3 months of follow-up. At interim analyses, the PRACT will 1) continue the enrollment, 2) adapt to another Stage according to predefined criteria, or 3) end the study for success or futility. Success in Stage 1 will be defined as the randomization to the intervention arm (PPKAY + Personalized Text Booster or PPKAY + Standard Text Booster) demonstrating superiority to Usual Care in the difference in reduction of number of binge drinking days per month, with 80% power to identify the necessary effect at interim analysis. If a stopping rule is reached, we will progress to Stage 2 and stop enrollment in UC. Futility in Stage 1 will be defined if no statistical difference is detected after maximum enrollment (N=205 per arm) in Stage 1. Assessing success in Stage 2 will be defined in a three-way non-inferiority evaluation of PPKAY alone, PPKAY + Standard Text Booster, and PPKAY + Personalized Text Booster. Successful analyses for Stage 2 is defined as one arm shown to be non-inferior than another.

4.2 SCIENTIFIC RATIONALE FOR STUDY DESIGN

We will use an innovative Pragmatic Randomized Adaptive Controlled Trial (PRACT) to rapidly improve and evaluate the effectiveness of our BI. While the BI has strong global evidence, there are no efficacy or effectiveness evaluations in Tanzania, and little data in Africa. There is already strong efficacy evidence supporting a BI to reduce alcohol -related complications for ED patients. Saitz has suggested that to date, BI effects seen in efficacy trials have not been translated into effectiveness trials. The author further suggests that researchers focus on how to implement alcohol interventions to retain the efficacy seen in clinical trials. In our low-resource ED setting, where expediting our trials can provide the only alcohol treatment option, we have chosen an effectiveness trial that emphasizes the intervention fidelity to rapidly progress to real-world implementation of this much needed intervention. Thus, a pragmatic trial determining our BI's real-world effectiveness in the Tanzanian clinical context allows for realistic integration into standard care. Our pragmatic trial will use Tanzanian nurses and will integrate the intervention into the current environment. While a recent pragmatic trial, the SIPS-ED trial showed minimal to no reduction in alcohol use, many suggest this was due to poor intervention fidelity/feasibility. Therefore, our study uses Tanzanian nurses dedicated to delivering the intervention, which will ensure intervention fidelity.

A traditional approach to investigating intervention adjuvants would include multiple, successive randomized clinical trials, each with specific research questions requiring significant time and financial resources. Given our aims, if we used a traditional randomized design we would have to enroll additional patients compared with our proposed minimum of 966 patients. Given the global literature support for our BIs and limited current treatments in Tanzania, our use of an adaptive trial will allow us to both show effectiveness over usual care while ensuring that most patients can receive this potentially

beneficial intervention. Similarly, our innovative PRACT highlights the strengths of an adaptive trial using an adaptive method during the phases where we expect a large effect size.

4.3 JUSTIFICATION FOR INTERVENTION

BI Intervention Justification

In high-income settings, a brief intervention (BI) administered in an Emergency Department (ED) setting for patients with AUDs cost-effectively reduces a patient's alcohol intake and re-injury rate up to 3 years postintervention. Numerous meta-analyses have documented this effect for both primary care and ED settings. While a recent pragmatic trial, the SIPS-ED trial, showed minimal to no reduction in alcohol use, many suggest this was due to poor intervention fidelity.

While the premise of BIs is well founded, there is limited literature about this intervention's impact in low-income country settings, nor the optimal length, frequency and content of interventions. There have been numerous studies in Africa on BIs, but the vast majority have occurred in South Africa and in non-ED settings. Sorsdahl et al. conducted a feasibility and clinical trial of a BI versus other intervention methods (problem solving therapy) in the ED setting in Cape Town, South Africa. Yet, these studies are set in a far more resourced environment in South Africa and require more advanced counseling capacity than is a vailable in Tanzania's setting. In low- and middle-income countries (LMICs), such as Tanzania, politicians, planners, and healthcare practitioners are more focused on those with alcohol dependence rather than the more highly functioning harmful and hazardous drinkers, who suffer most of the alcohol -related consequences. Accordingly, treatment in LMICs, if there is any, has been focused on long-term residential treatment centers with limited efficacy. But Benegal et al. suggest a step-wise implementation of alcohol interventions, starting with opportunistic screening and brief advice and progressing to other, more complex treatment options.38 However, the optimal format of such interventions is not evident. For example, it is not clear whether the additional complexity of using personalized boosters has added value in this limited resource context.

Our team has created and piloted "Punguza Pombe Kwa Afya Yako" (PPKAY)/ "Reduce Alcohol for Your Health"; a brief intervention for the KCMC ED. PPKAY was based upon NIAAA BI suggested standards, adapted to Swahili and the Tanzanian environment, and piloted during prior NIH-funded work in Tanzania (See Methods and Intervention Development). This is a one-time, 15-minute, nurse-led intervention based on motivational interviewing principles, discusses safe drinking behaviors, and negotiates change in alcohol use. Our trial compares PPKAY to usual care of no alcohol harm prevention education.

In Tanzania, similar to other low-resource settings, there are limited treatment options for AUDs. Siloed care, an overburdened healthcare system, and under recognition of alcohol as a priority have limited alcohol -focused regulation and prevention initiatives. Seeking care in low-resource settings is a

prohibitively expensive and time-consuming endeavor. Therefore, care seeking occurs mainly for acute events. Acute injuries are a leading cause of ED utilization, and highly associated with alcohol use. In high-income settings, patients who present to an ED for care of injuries are more likely than the general population to have AUDs; also, ED injury patients with AUDs have a higher mortality risk after discharge than those without. Patients who suffer an injury after drinking alcohol who require ED-based care are, by definition, harmful alcohol users who have a very high risk of repeated alcohol-related complications. In high-income settings, medical providers have harnessed these potential teachable moments when harmful alcohol users face their injury complications. A brief intervention based on motivational interviewing during this teachable moment to reduce alcohol use increases a patient's chance of changing behavior. In high-income setting EDs, challenges to implementing an alcohol intervention are well described; limitations in funding and time, and providers believing it is not their role are the most common barriers.

SMS Intervention Justification

Based on the strength of the BI literature for US injury patients, the American College of Surgeons has now mandated a BI for all injured patients with harmful or hazardous alcohol use. But to date, its optimal implementation is still controversial. For instance, some have found boosters, or intermittent post-intervention reminders, to be helpful, while others have found no improvement. Similarly, integrating mobile health technology, such as text messaging, into a BI has shown promise, but also carries resource challenges, and is unproven in a low-resource context. Finally, in a high-income setting, personalized boosters recounting the specific reasons for a patient's behavior change have been shown to increase effectiveness at no increased cost. However, that has not been tested in low-income settings where the resources required to personalize boosters might not be available and might add unnecessary complexity.

We will compare the effectiveness of PPKAY with an augmented version. PPKAY will be successively augmented in two ways: the inclusion of a standard text-based booster and a personalized text-based booster. Our feasibility study has shown that mobile technology is ubiquitous with over 95% of our population having a text capable cell phone; similarly, a mobile health platform has been successful at KCMC for intervention and follow-up studies. While smartphone technology and internet costs are challenging in the Tanzanian context, text-based communication is reliable, inexpensive, adaptable to the research environment and available to most of our population. Modeling our boosters on evidence from high-income settings,67 our standard booster will remind patients of the benefits of alcohol reduction, while our personalized booster will reiterate the patient’s own reasons for alcohol use reduction obtained during the BI. While high-income setting data have shown a personalized booster is more effective, this remains to be shown in a low-income setting. Simple reproducible interventions may be best in a limited resource setting, so a standard booster developed a priori might be a less complex, higher quality intervention with more fidelity and effectiveness.

4.4 END-OF-STUDY DEFINITION

A participant is considered to have completed the study if he or she has completed the baseline assessment, follow-up assessments at 3, 6, 9, 12 and 24 months post-discharge.

5 STUDY POPULATION

5.1 INCLUSION CRITERIA

Inclusion Criteria: We will offer informed consent to adult patients (≥18 years of age) who present seeking initial care at the KCMC ED for an acute (<24 hours) injury who are not clinically intoxicated (i.e., have capacity to consent). The patient's clinician will determine capacity to consent based on history and physical exam. Patients who are intoxicated or too ill on arrival to the ED will be reassessed for up to 24 hours to gauge if they have regained capacity to consent. After consent is obtained, patients will need to have one of the following eligibility criteria: 1) disclosed alcohol use prior to injury, 2) scored ≥8 on the AUDIT or 3) test positive (>0.0 g/dL) by alcohol breathalyzer.

5.2 EXCLUSION CRITERIA

Exclusion Criteria: Patients will be excluded if they do not speak the native Swahili language, they are too ill or unable to communicate, they have been enrolled in this study already, or they decline informed consent.

5.3 LIFESTYLE CONSIDERATIONS

N/A

5.4 SCREEN FAILURES

Screen failures are defined as participants who consent to participate in this study, but are not subsequently assigned to the study intervention or entered in the study. Individuals who do not meet the criteria for participation in this trial (screen failure) because of meeting one or more exclusion criteria that are may change over time **may be rescreened** if they visit the KCMC ED for a subsequent injury.

5.5 STRATEGIES FOR RECRUITMENT AND RETENTION

Adaptive Screening Size: The number of patients screened depends on the effect of the intervention as determined during interim analyses. Based on previous studies, the anticipated screening load will involve approximately 16 patients per week. If effect size is small, screening and enrollment may extend for a maximum of 33 months, resulting in a maximum of approximately 2,200 patients screened. If effect size is large, screening and enrollment may be limited to a minimum of 21 months.

Adaptive Sample Size and Enrollment: The sample size was calculated for a change in the primary outcome of reduction in the number binge drinking days per month. Depending on the effect size found at each interim analysis, it is possible that significance could be reached earlier than expected. For Stage 1, recent literature supports effect sizes of 60% decrease. Besides, there is no intervention currently available for alcohol use reduction post injury in Tanzania. Through adaptive sample re-estimation and the most effective scenario, total minimum enrollment could be as low as 520 patients. Hypothesis testing was adjusted at interim analysis according to the O'Brien-Fleming alpha-spending function. Thus, instead of spreading the alpha equally across interim analysis, the distribution is progressive leaving most of the significance levels later on the trial, when more patients are enrolled. If challenges arise with the adaptive nature of this trial, we will resort to standard sequential RCTs with maximum enrollment numbers. If standard RCT enrollment is used, an adequate sample size will be obtained to show the same effect size with higher power.

Sample Characteristics: Our study will enroll patients who present to Kilimanjaro Christian Medical Center in Moshi, Tanzania. We encourage participation of all those who present for care into our study regardless of age, sex, gender, race or ethnicity. Due to our setting, we anticipate that nearly 100% of our enrolled patients will be Africans. Similarly, the majority of injury patients in this setting are males (80%) according to previous studies. We will continue to encourage the participation of all local tribes (Chagga, Masai, Saama etc.) in our study. We will not exclude patients based on age, sex, race, gender, or ethnicity.

Recruitment: After screening for age (≥ 18 years) and first visit for an acute (<24 hours) injury, patients who present to the Kilimanjaro Christian Medical Center Emergency Department will be offered an informed consent. If they agree to participate, they will undergo pre-randomization screening, which includes breathalyzer testing, AUDIT tool administration and questions about alcohol use prior to injury. If they fit the inclusion criteria based on their pre-randomization screening, they will be randomized. Study nurses will be present in the emergency department 7 days a week, 96 hours a week in order to identify potential participants for recruitment. If patients are to be discharged home, they will be enrolled prior to discharge. If they are admitted to the hospital, they will be enrolled when they are medically stable and at the most convenient time period for the patient. We will do no further recruitment than screening patients who present to the emergency department.

Patient Privacy: Patient privacy will be maintained by keeping all records in an anonymous fashion. Screening and data collection will occur in a quiet, private room near the Emergency Department. None of the results from screening or this study will be shared with anyone other than the treating physician. Any communication over phone between researchers and participants will occur over a phone number that the patient provides. Any text-based boosters will be vague enough to ensure no loss of confidential information in case phones are accessed by someone other than the intended participant.

Study Site: All recruitment for this study will occur at the Kilimanjaro Christian Medical Centre in Moshi, Tanzania. This tertiary care hospital is an academic teaching hospital and referral hospital for the Northern Tanzania region. Based on preliminary data, 2000 injury patients are seen annually in the KCMC ED (38 patients per week).

Retention: During enrollment, at least two phone numbers will be identified and tested by participants for follow-up needs. Nurses maintain contact with patients during the follow-up period and make phone calls to set up follow-up assessments. To ensure follow-up, if needed, we travel to patients, financially support their travel to us, or conduct follow-ups over the phone. Our research team has over 5 years follow-up experience in this population, including collecting family phone numbers and other 'locator' information with patient permission. Participants are not paid for participation but transportation is reimbursed and food and drinks are provided for on-site follow-up. These methods have ensured an 83% retention at 6-month follow-up in our feasibility trial.

Participant Compensation: We will reimburse 5000 TSH (approx. $2 US) per participant for each follow-up visit completed. Participants will be reimbursed with cash or with cell phone minutes of the same cost.

Vulnerable Populations: We will attempt consecutive screening of all adult patients who arrive to the KCMC ED for acute care during the enrollment hours. Participants will be approached if they suffered an injury, regardless if they are part of a potential vulnerable population (e.g., pregnant women). Prisoners are not able to have phone contact with others, therefore would be excluded from our study.

We do not anticipate that any participants in our study would be members of vulnerable populations. Regardless, we are not excluding persons based on their gender, so some women might be pregnant. It is not commonplace to test women presenting with an injury for pregnancy in this setting, so potential pregnancies may remain unknown. If we do enroll a pregnant individual, excluding her from this potentially beneficial intervention for alcohol without any alternative treatments would be unethical.

We also do not anticipate that there would be any prisoners who are enrolled in our study, but since they are not allowed to have phone contact, prisoners would need to be excluded as they cannot take part in either the complete intervention nor in the follow-up for this study.

6 STUDY INTERVENTION(S) OR EXPERIMENTAL MANIPULATION(S)

6.1 STUDY INTERVENTION(S) OR EXPERIMENTAL MANIPULATION(S) ADMINISTRATION

6.1.1 STUDY INTERVENTION OR EXPERIMENTAL MANIPULATION DESCRIPTION

Trial Arms

	**Usual Care**	**PPKAY**	**PPKAY with Standard Booster**	**PPKAY with Personalized Booster**
**Intervention**	No prevention or educational information verbally or written coinciding with current usual care	One-time, 15-minute, nurse-led motivational interview discussing safe drinking behaviors, and negotiating change in alcohol use	One-time, 15-minute, nurse-led motivational interview discussing safe drinking behaviors, and negotiating change in alcohol use Weekly standard text booster until final follow-up (e.g., “Reducing your alcohol intake to less than 4 drinks per day reduces your risk of alcohol-related consequences”)	One-time, 15-minute, nurse-led motivational interview discussing safe drinking behaviors, and negotiating change in alcohol use Weekly personalized text booster until final follow-up (e.g., “Remember to reduce your alcohol less than 4 drinks to achieve your goal of… [being a better husband].”)
**Follow-up**	3, 6, 9, 12, 24 months			
**Outcomes**	Alcohol Use Disorder Identification Test (AUDIT); Alcohol-related harms (DrInC); Quantity/ Frequency; Number of binge drinking days; Depression measure (PHQ-9)			

PPKAY=Punguza Pombe Kwa Afya Yako (“Reduce Alcohol For Your Health”)

6.1.2 ADMINISTRATION AND/OR DOSING

PPKAY: We created a nurse administered, one-on-one, 15-minute brief intervention (BI) using FRAMES motivational interviewing techniques. The BI is a four-step discussion: 1) Raise the Subject of Alcohol, 2) Provide Feedback, 3) Enhance Motivation 4) Negotiate and Advice. Counselors will approach patients and ask permission to discuss alcohol and their current alcohol use compared to international safe use guidelines. Counselors will assess the patient's motivation to change using a piloted and validated Readiness Ruler. Counselors will then identify a patient's specific reasons for changing behavior (social, financial etc.) and compare it to their Readiness Ruler response, highlighting self-efficacy and personal capacity for behavior change. Finally, the counselor will negotiate a reduction in alcohol use, optimally lower than international use guidelines. At the close of the BI, the provider will thank the patient for their openness in discussing alcohol and continue to promote self-efficacy.

PPKAY with Standard Booster: Patients allocated to the BI with Standard Booster will undergo the same BI process during their enrollment period listed above. They will provide a phone number where they can be reached, and this cell phone will be texted by the study team to ensure they are reachable. After discharge from the hospital, a standard motivational text message will be sent to their cell phone twice monthly for the duration of the study. In a rotating fashion, one of four standard motivational texts translated into Swahili will be sent to their cell phones.

PPKAY with Personalized Booster: Patients allocated to the BI with Personalized Booster will undergo the same BI process and text booster processes listed above. After discharge from the hospital, a personalized motivational text message will be sent to participant cell phones twice monthly for the duration of the study. In a rotating fashion, one of four personalized motivational texts will be sent to their cell phones. Personalized texts will be created with the content obtained from the motivational interview sessions. At each session, the research nurse conducting the intervention will record four messages based on the content of the session to be sent as the Personalized Booster.

6.2 FIDELITY

6.2.1 INTERVENTIONIST TRAINING AND TRACKING

<u>PPKAY</u>

Training: During a feasibility trial, our bilingual research nurses were trained by Dr. Staton in Good Clinical Practices as well as our PPKAY. We plan annual nurse training and evaluation to maintain a group of nurses who can conduct a high-quality intervention.

Fidelity: To ensure intervention fidelity, a bilingual researcher with experience administering PPKAY will evaluate audiotaped interventions and give intermittent feedback to nurses on the intervention adherence to motivational interviewing guidelines and the BNI Assessment Scale. Our team has adapted and validated the BNI Assessment Scale to the Tanzanian setting. This adapted BI Assessment Scale was found to have good psychometric properties including inter- and intra-rater reliability (Kappa and ICC values above 0.80). We noted a strong ability to differentiate deviations in the intervention protocol by comparing standard PPKAY sessions with poorly performed PPKAY sessions. During the feasibility trial, our nurses were assessed using this BI Assessment Scale and have shown to have high-fidelity scores as measured by this scale and by external expert opinion. During our study, we will audio record the interventions and have these interventions evaluated weekly by bilingual researchers to determine

adherence to the intervention plan using our validated scale. If we find intervention protocol deviations, educational feedback will be provided to the nurse interventionists.

Standard and Personalized Booster

Training: Kennedy Ngowi, the investigator managing our SMS system, has worked on numerous projects (including the feasibility trial related to the present study), which employ the ubiquitous use of mobile phones in Tanzania to improve patient treatment and wellness.

Fidelity: Mr. Ngowi will prepare monthly reports summarizing: the number and content of message transmissions attempted, number of message transmission failures and successes, reasons for transmission failure, number of participants opting out of messages, and incoming message content.

6.3 MEASURES TO MINIMIZE BIAS: RANDOMIZATION AND BLINDING

In an adaptive clinical trial, investigator level blinding is key to limiting biases during the adaptations and analysis, while blinding at the nursing level will limit the bias at the patient level. Our unblinded analysis leader, Dr. Vissoci, will conduct the analysis and bring the data to the DSMB for them to determine if we have reached a priori established adaptation decision points. Our DSMB will evaluate our preliminary analysis data, oversee the sample size re-estimation based on up-to-date trial effect size data and decide if and when adaptations are implemented, as investigators and research personnel will remain blinded whenever possible. Since Dr. Vissoci will manage the data management, the data quality improvement processes and present the data to the DSMB for decision-making, he will remain unblinded. To ensure rigorous data, intensive quality assurance processes and audits are planned, which blinded investigators cannot conduct. Thus, Dr. Vissoci must remain unblinded. Dr. Vissoci will also have no role in the management of the clinical trial in Tanzania to ensure we limit any potential biases. At the Research Nurse level, we have established roles so that outcomes assessors are blinded to a patient’s allocation.

6.4 STUDY INTERVENTION/EXPERIMENTAL MANIPULATION ADHERENCE

Not Applicable

6.5 CONCOMITANT THERAPY

N/A

6.5.1 RESCUE THERAPY

This motivational alcohol reduction intervention has no treatment alternative available in the region, so there are no alternatives to suggest for these patients. We do not anticipate any medication-related issues for this behavioral health trial. Psychological stress triggered by our brief intervention or any subsequent boosters is a potential risk for patients. Patients will be told they may discontinue their enrollment at any time during this trial by contacting the research team and/or the site PI. Our research nurses are trained counselors who will be able to assist patients should they suffer increased psychological stress during this intervention. For more aggressive mental health assistance (for potential self-harm), patients will be referred for further treatment at a local mental health provider.

7 STUDY INTERVENTION/EXPERIMENTAL MANIPULATION DISCONTINUATION AND PARTICIPANT DISCONTINUATION/WITHDRAWAL

7.1 DISCONTINUATION OF STUDY INTERVENTION/EXPERIMENTAL MANIPULATION

Patients will be told they may discontinue their enrollment at any time during this trial by contacting the research team and/or the site PI. Discontinuation of the PPKAY intervention is not anticipated, as it is a one-time, 15-minute intervention. Continued receipt of intervention messages for those enrolled in Standard Booster or Personalized Booster arms can be discontinued by the participant responding "S" or "STOP" to the text messages. This method of withdrawal from the intervention is explained in the initial message sent to the participants. If participants discontinue participation in the booster interventions, they may continue to participate in research follow-up visits.

For this study, we will gather information on Adverse Events (AEs), Serious Adverse Events (SAEs) and unanticipated problems involving risks to subjects or others. Each of these events will be reported by research personnel to the PI or Tanzanian site PI within 1 week of finding out the information. Non-compliance with this reporting guideline for research personnel could lead to suspension or termination. It is the responsibility of the Tanzanian site PI to relay this information to the PI as soon as possible. The PI will be responsible for reporting any AE, SAE or unanticipated problems to the DSMB as well as appropriate regulatory bodies within 1 week. As a rule, blinding will be maintained for all patients with AEs, SAEs and unanticipated problems. If the DSMB determines that unblinding of the allocation group of the patients who suffered this event for the protection of other trial participants, this can occur. Unblinding a patient’s allocation arm can also occur should the patients be in an emergency where the patient’s allocation could potentially impact their health; given our type of intervention, this case is very unlikely to occur.

7.2 PARTICIPANT DISCONTINUATION/WITHDRAWAL FROM THE STUDY

Participants may withdraw voluntarily from the study data collection or the study intervention at any time.

Participants may be withdrawn (by the study team) from data collection or intervention if

- They permanently move out of the country
- They are imprisoned
- A medical condition develops or a situation occurs such that continued collection of follow-up study data would not be in the best interest of the participant or might require an additional treatment that would confound the interpretation of the study's results

7.3 LOST TO FOLLOW-UP

A participant will be considered lost to follow-up if he or she fails to return for all scheduled visits, and study staff are unable to contact the participant after at least 3 attempts related to each follow-up appointment.

The following actions must be taken if a participant fails to return to complete a follow-up study visit:

- The research staff will counsel the participant on the importance of maintaining the assigned visit schedule and ascertain if the participant wishes to and/or should continue in the study
- Before a participant is deemed lost to follow-up, the study staff will make every effort to regain contact with the participant (where possible, 3 telephone calls per planned study visit). These contact attempts will be documented in the participant's study file.
- Should the participant continue to be unreachable, he or she will be considered to have withdrawn from the study with a primary reason of lost to follow-up

8 STUDY ASSESSMENTS AND PROCEDURES

8.1 ENDPOINT AND OTHER NON-SAFETY ASSESSMENTS

Procedures and Assessments

Pre-screening assessment
Upon arrival to the KCMC ED, patient charts will be pre-screened and patients asked preliminary questions to determine:

- If injury was within 24 hours
- If patient age is greater than 18 years
- If patient is clinically sober, as determined by the treating physician
- If patient is fluent in Swahili
- If patient has a phone capable of receiving text messages

Consent
If the patient passes the pre-screening assessment, he or she will be offered informed consent.

Screening assessment
Consenting patients will be screened. Screening assessments measure:

- Date and time of injury
- Alcohol use (type and quantity) in the 6 hours prior to injury
- Alcohol status (breathalyzer)
- Demographic information
    - Religion (type and importance)
    - Education level and years
    - Employment status and type
    - Tribe, Race, and Ethnicity
- Alcohol use disorder (AUDIT)

If the patient reports (A) alcohol use within 6 hours of injury, (B) positive breathalyzer, or (C) AUDIT score ≥8, the patient will be randomized and issued a study ID#.

Baseline Assessment

Upon enrollment, baseline assessment will measure:

- Alcohol-related harms (Drinker Inventory of Consequences (DrInC))
- Severity of AUD (DSM criteria)
- Injury characteristics
  - Mechanism of injury
  - Intention of injury
  - Location of injury on body
  - Perceived seriousness of injury
  - Clinical severity of injury
  - Alcohol's relation to injury
- Patient's functionality
- Depression symptoms (PHQ-9)
- Resilience and Coping (Brief Resilience Scale), Coping Inventory for Stressful Situations)
- Alcohol expectancies (East Africa Alcohol Expectancy Scale)
- Motivation for alcohol control (TSRQ Alcohol)
- Alcohol control confidence (Brief Situational Confidence Questionnaire)
- Perceived Alcohol Stigma (Perceived Devaluation-Discrimination scale)
- Perceived Social Support
- Food security (Household Food Insecurity Access Scale (HFIAS) Generic Questions)
- PTSD symptoms (PTSD Scale for Primary Care)
- Anxiety Symptoms (GAD-7)

Follow-up Assessment

At 3, 6, 9, 12, and 24 months post-discharge, follow-up assessments will measure:

- Alcohol-related harms (Drinker Inventory of Consequences (DrInC))
- Severity of AUD (DSM criteria)
- Post-discharge injuries
- Post-discharge emergency room visits

- Patient's functionality
- Depression symptoms (PHQ-9)
- Resilience and Coping (Brief Resilience Scale), Coping Inventory for Stressful Situations)
- Alcohol expectancies (East Africa Alcohol Expectancy Scale)
- Motivation for alcohol control (TSRQ Alcohol)
- Alcohol control confidence (Brief Situational Confidence Questionnaire)
- Perceived Alcohol Stigma (Perceived Devaluation-Discrimination scale)
- Perceived Social Support
- Food security (Household Food Insecurity Access Scale (HFIAS) Generic Questions)
- PTSD symptoms (PTSD Scale for Primary Care)
- Anxiety Symptoms (GAD-7)
- Intervention adherence

Qualitative Interviews: At 24-month follow up, we will conduct qualitative interviews to further understand potential mediators or moderators of the intervention. These will include the participation of up to 12 women and 12 men (already enrolled in the study) and those with and without certain mental health indicators, such as high PHQ-9 scores. We will complete data collection when we reach saturation or no new thematic ideas.

8.2 SAFETY ASSESSMENTS

Once enrolled in the study, the specific events which would preclude participants from continuing in the trial are death or incapacitating injury. This motivational alcohol reduction intervention has no treatment alternative available in the region, so there are no alternatives to suggest for these patients. We do not anticipate any medication-related issues for this behavioral health trial. Psychological stress triggered by our brief intervention or any subsequent boosters is a potential risk for patients. Patients will be told they may discontinue their enrollment at any time during this trial by contacting the research team and/or the site PI. Our research nurses are trained counselors who will be able to assist patients should they suffer increased psychological stress during this intervention. For more aggressive mental health assistance (for potential self-harm), patients will be referred for further treatment at a local mental health provider.

8.3 ADVERSE EVENTS AND SERIOUS ADVERSE EVENTS

8.3.1 DEFINITION OF ADVERSE EVENTS

In accordance with Duke University Health System (DUHS) policy, a non-serious adverse experience or event requires prompt reporting when it both: (a) is unanticipated and (b) indicates that the research places subjects or others at a greater risk of harm (including physical, psychological, economic, or social harm) than was previously known or recognized.
Adverse events include any unfavorable medical change that occurs during or after beginning the study that may or may not be related to the study treatments.

8.3.2 DEFINITION OF SERIOUS ADVERSE EVENTS

In accordance with Duke University Health System (DUHS) policy
A serious adverse experience or event requires prompt reporting when it is all of the following:
(a) unanticipated
(b) indicates that the research places subjects or others at a greater risk of harm (including physical, psychological, economic, or social harm) than was previously known or recognized.
(c) life-threatening, OR requires hospitalization, OR results in persistent or significant disability, OR results in congenital abnormality, birth defect, or death OR is another significant medical event.

8.3.3 CLASSIFICATION OF AN ADVERSE EVENT

8.3.3.1 SEVERITY OF EVENT

8.3.3.2 RELATIONSHIP TO STUDY INTERVENTION/EXPERIMENTAL MANIPULATION

8.3.3.3 EXPECTEDNESS

8.3.4 TIME PERIOD AND FREQUENCY FOR EVENT ASSESSMENT AND FOLLOW-UP

8.3.5 ADVERSE EVENT REPORTING

Within ten business days of the investigator becoming aware of the event, study personnel will send to the IRB a Safety Event submission in the eIRB.

8.3.6 SERIOUS ADVERSE EVENT REPORTING

Immediately (within 24 hours) upon learning of an unanticipated study-related death, study personnel will notify by providing a brief summary of the event. Then, within 1 week (five business days), study personnel will send to the IRB a Safety Event submission in iRIS.

For a reportable serious adverse event, study personnel will notify the IRB within five business days of the investigator becoming aware of the event. Study personnel will send a Safety Event submission in iRIS.

8.3.7 REPORTING EVENTS TO PARTICIPANTS

We will inform participants of any significant new findings developed during the course of the research which may affect the participants' willingness to continue in the study. Participants can be notified by signing a IRB-approved information letter or an updated revised consent form.

8.3.8 EVENTS OF SPECIAL INTEREST

8.3.9 REPORTING OF PREGNANCY

We do not anticipate that any participants in our study would be pregnant. Regardless, we are not excluding persons based on their gender, so some women might be pregnant. It is not commonplace to test women presenting with an injury for pregnancy in this setting, so potential pregnancies may remain unknown.

8.4 UNANTICIPATED PROBLEMS

8.4.1 DEFINITION OF UNANTICIPATED PROBLEMS

In accordance with Duke University Health System (DUHS) policy, an unanticipated problem is any incident, experience, or outcome that is both (a) unanticipated and (b) indicative of the research placing subjects or others at a greater risk of harm (including physical, psychological, economic, or social harm) than was previously known or recognized.

8.4.2 UNANTICIPATED PROBLEMS REPORTING

Immediately (within 24 hours) upon learning of an unanticipated study-related death, study personnel will notify the DUHS IRB via phone or email by providing a brief summary of the event. Then within 1 week (five business days), study personnel will send to the DUHS IRB a Safety Event submission in iRIS. For any other problem or event requiring prompt reporting to the IRB, within ten business days of the site investigator becoming aware of the event, study personnel will send to the DUHS IRB a Safety Event submission in iRIS. It is the responsibility of the Tanzanian site PI to relay this information to the PI as soon as possible. The PI will be responsible for reporting any unanticipated problems to the DSMB, DUHS IRB, the KCMC Ethics Committee, and the Tanzanian National Institute for Medical Research within 1 week.

8.4.3 REPORTING UNANTICIPATED PROBLEMS TO PARTICIPANTS

We will inform participants of any significant new findings developed during the course of the research which may affect the participants' willingness to continue in the study. Participants can be notified by signing a IRB-approved information letter or an updated revised consent form.

9 STATISTICAL CONSIDERATIONS

9.1 STATISTICAL HYPOTHESES

Primary Efficacy Endpoint: A measure of binge-drinking days per month is our primary endpoint for adapting and assessing success in this trial. Binge-drinking days will be compared across trial arms to test the following hypotheses:

- Phase 1: The intervention (PPKAY + Personalized Text Booster or PPKAY + Standard Text Booster) arms will result in less # of binge drinking days per month up to 3 months after hospital discharge, relative to those in the Usual Care arm.
- Phase 2: PPKAY alone is non-inferior to either booster intervention arm in reducing the # of binge drinking days per month up to 3 months after hospital discharge.

9.2 SAMPLE SIZE DETERMINATION

Sample size

Stage 1: An effective intervention for Stage 1 will be defined as having an effect size of at least 35% difference in the reduction in the primary outcome (number of binge drinking events per month), between control and intervention arms. Assuming an average of four binge drinking days per month (SD = 3.4), a 35% difference translates into a difference of about 1.2 days in the reduction in the number of binge drinking days per month. Evidence from studies in high-income settings suggests that studies similar to Stage 1 have shown moderate to high treatment effects of 20 - 40% in the difference. A sample size of 205 patients per arm will have 80% power to be able to identify the best study arm with a significance of 5%, and a conservative 80% retention rate.

Stage 2: A total of 324 participants will be required for Stages 2. Enrollment numbers carried forward from Stage 1 will allow allocation to be lower in those arms, thus will enroll at a 4:1:1 ratio, favoring the PPKAY-only arm. This sample size will allow for testing the non-inferiority of PPKAY alone in comparison to the PPKAY + Personalized Text Booster and the PPKAY + Standard Text Booster. This sample would be enough to identify non-inferiority with a plausible futility margin of 0.9 binge drinking day per month (half of the effect of the intervention against Usual Care in Stage 1), assuming a standard deviation of the difference at 3.4, 80% power, 5% significance level and 80% retention rate. Currently there are no studies similar to Stage 2 for ED injury patients to model the effect size estimation. Since Stage 2 analyses will include patients enrolled from Stage 1, in contrast to a typical trial, there will be smaller maximum enrollment per stage.

Interim Analyses and Adaptations:
In Stage 1, up to 3 interim analyses will be conducted after the first batch of patients complete 3 months of follow-up. At interim analyses, the PRACT will 1) continue the enrollment, 2) adapt to another Stage according to predefined criteria, or 3) end the study for success or futility. Success in Stage 1 will be defined as the randomization to the intervention arm (PPKAY + Personalized Text Booster or PPKAY + Standard Text Booster) demonstrating superiority to Usual Care in the difference in reduction of number of binge drinking days per month, with 80% power to identify the necessary effect at interim analysis. If a stopping rule is reached, we will progress to Stage 2 and stop enrollment in UC. Futility in Stage 1 will be defined if no statistical difference is detected after maximum enrollment (N=205 per arm) in Stage 1. Assessing success in Stage 2 will be defined in non-inferiority evaluation of PPKAY alone compared to PPKAY + Standard Text Booster and PPKAY + Personalized Text Booster. Successful analyses for Stage 2 is defined as reaching a point where one arm is shown to be non-inferior to another

Adaptive Sample Size: The sample size was calculated for a change in the primary outcome of reduction in the number binge drinking days per month. Depending on the effect size found at each interim analysis, it is possible that significance could be reached earlier than expected. For Stage 1, recent literature supports effect sizes of 60% decrease. Besides, there is no intervention currently available for alcohol use reduction post injury in Tanzania. Through adaptive sample re-estimation and the most effective scenario, total minimum enrollment could be as low as 520 patients. Hypothesis testing was adjusted at interim analysis according to the O'Brien-Fleming alpha-spending function.[163] Thus, instead of spreading the alpha equally across interim analysis, the distribution is progressive leaving most of the significance levels later on the trial, when more patients are enrolled.

Maximum Enrollment: If we encounter challenges with the adaptive nature of this trial, we will resort to standard sequential RCTs with maximum enrollment numbers. If we use standard RCT enrollment, we will have an adequate sample size to show the same effect size with more power.

9.3 POPULATIONS FOR ANALYSES

Intention-to-treat analyses will be used to include all randomized patients in statistical analyses according to the assigned trial arm, regardless of the treatment ultimately received.

Missing data will be handled by multiple imputation and inverse weighting design with sequential sensitivity analysis to evaluate the impact on the effect of the trial.

9.4 STATISTICAL ANALYSES

9.4.1 GENERAL APPROACH

Patient demographics will be collected and compared across all arms of the study. Descriptive data will be reported as means, standard deviations, and frequencies. Outcome data will be reported as predicted means with confidence intervals for each follow up time point (3, 6, and 12 months), depicting the optimal intervention at each phase. Missing data will be handled by multiple imputation and inverse weighting design with sequential sensitivity analysis to evaluate impact on the effect of the trial.

9.4.2 ANALYSIS OF THE PRIMARY ENDPOINT(S)

To evaluate the efficacy of both intervention arms in Phase 1 in relation to the control group, generalized linear model will be constructed, allowing for the prediction of follow-up scores after controlling for participants' baseline characteristics and other potential confounders (potential predictors of attrition and outcome confounders). Count models will be used for the frequency of alcohol-related complications, frequency of drinking and frequency of binge drinking. Given our previous observational data, we expect that the data distribution will best fit a negative binomial method with a log link function, a recommended approach for overdispersed alcohol consumption data. For AUDIT score and quantity of drinking, linear models will be used. For significant interaction terms, contrasts of marginal means will be conducted and the Wald chi-squared test statistic interpreted. Effect size will be reported as incidence risk ratios (for count data) and the difference in the predicted means (for numeric data), to highlight the magnitude of difference between groups. Interim analysis will be conducted at each new month of complete follow up and will signal the end of the Phase 1 enrollment if the required effect size is reached (Table 1) or until the maximum sample size is reached.

Power was set at 80% and 2.5 percent (two tailed) Type I error.

9.4.5 BASELINE DESCRIPTIVE STATISTICS

Preliminary analysis will be conducted to evaluate differences at baseline and differences in attrition among all study arms. These comparisons will be made using Student's t-test for continuous variables or Mann-Whitney U test according to the data distribution, and the Pearson chi-squared test for categorical variables.

9.4.6 PLANNED INTERIM ANALYSES

In Stage 1, up to 3 interim analyses will be conducted after the first batch of patients complete 3 months of follow-up. At interim analyses, the PRACT will 1) continue the enrollment, 2) adapt to another Stage according to predefined criteria, or 3) end the study for success or futility. Success in Stage 1 will be defined as the randomization to the intervention arm (PPKAY + Personalized Text Booster or PPKAY + Standard Text Booster) demonstrating superiority to Usual Care in the difference in reduction of number of binge drinking days per month, with 80% power to identify the necessary effect at interim analysis. If a stopping rule is reached, we will progress to Stage 2 and stop enrollment in UC. Futility in Stage 1 will be defined if no statistical difference is detected after maximum enrollment (N=205 per arm) in Stage 1. Assessing success in Stage 2 will be defined in non-inferiority evaluation of PPKAY alone versus PPKAY +

Standard Text Booster and PPKAY + Personalized Text Booster. Successful analyses for Stage 2 is defined as reaching a point where one of the arms is non-inferior to the other.

9.4.7 SUB-GROUP ANALYSES

For categorical moderators (e.g., sex), analyses of variance will be conducted with the moderator and intervention arm as factors and the baseline level of the dependent variable as a covariate. For numeric moderators (e.g., age), generalized linear models will be used. Multivariate regression will test the main effects of intervention, moderator, and the interaction of the intervention arm and the moderator for each outcome group.

Qualitative interview data are expected to elucidate these findings further.

9.4.8 TABULATION OF INDIVIDUAL PARTICIPANT DATA

Individual participant data will be listed by measure and time point.

10 SUPPORTING DOCUMENTATION AND OPERATIONAL CONSIDERATIONS

10.1 REGULATORY, ETHICAL, AND STUDY OVERSIGHT CONSIDERATIONS

10.1.1 INFORMED CONSENT PROCESS

10.1.1.1 CONSENT/ASSENT AND OTHER INFORMATIONAL DOCUMENTS PROVIDED TO PARTICIPANTS

Written informed consent forms will be offered to all participants. Consent forms describe the study intervention (with slight obscuration regaring the focus on alcohol), study procedures, and risks are given to the participant and written documentation of informed consent is required prior to study intervention.

10.1.1.2 CONSENT PROCEDURES AND DOCUMENTATION

Verbal and written informed consent will be obtained for all patients. During this process, research nurses read the written informed consent to the patients to ensure their comprehension given variable literacy rates. The informed consent process will take place after medical stabilization, when appropriate, and will occur in a private, quiet location by trained research nurses in the local language of Swahili. The research nurse will state the purpose of the study in a broad sense about wellness. For this study, as the outcomes are self-reported, a slight obscuration of the study goals is necessary to reduce potential social desirability and reporting biases. Similarly, risks and benefits of the study and the participant's responsibilities will be explained. All data collection will be performed in an anonymous manner to minimize participant risk.

10.1.2 STUDY DISCONTINUATION AND CLOSURE

This study may be temporarily suspended or prematurely terminated if there is sufficient cause. Written notification, documenting the reason for study suspension or termination, will be provided by the suspending or terminating party to study participants, investigators, funding agency, and regulatory authorities. If the study is prematurely terminated or suspended, the Principal Investigator (PI) will promptly inform all of the above of the reason(s) for the termination or suspension. Study participants will be contacted, as applicable, and be informed of changes to study visit schedule. Circumstances that may warrant termination or suspension include, but are not limited to:

- Determination of unexpected, significant, or unacceptable risk to participants
- Insufficient compliance of study staff to the protocol (i.e., significant protocol violations)
- Data that are not sufficiently complete and/or valuable

The study may resume once concerns about safety, protocol compliance, and data quality are addressed, and satisfy the funding agency, IRB, or other relevant regulatory or oversight bodies (OHRP, DSMB).

10.1.3 CONFIDENTIALITY AND PRIVACY

All data (screening, enrollment and intervention logs, questionnaires/ data collection sheets, audio recordings, translations and transcriptions) will be stored by study identification number. All hard copy paper will be stored by study identification number in locked cabinets in a locked office of the second floor of the KCMC CCFCC. Online data will be stored by study identification number behind the Duke firewall and accessible to those included in the regulatory process.

10.1.4 FUTURE USE OF STORED SPECIMENS AND DATA

10.1.5 KEY ROLES AND STUDY GOVERNANCE

**Principal Investigator**	**Data Safety Monitoring Board**
Catherine A. Staton	Roger Lewis, roger @ emedharbor.edu
+1 919 684 5537	David Wright, dwwrigh @ emory.edu
catherine.staton @ duke.edu	Kajiru Kilonzo, mtundumliasi @ googlemail.com
2301 Erwin Road, DUMC Box 3096	Sylvia Kaaya, skaaya @ gmail.com
Durham, NC 27710	

10.1.6 SAFETY OVERSIGHT

This PRACT will be monitored by a Data Safety Monitoring Board (DSMB) as seen above. Analysis lead investigator Dr. Vissoci will be responsible for assembling data to be presented at the DSMB meetings, which are expected to occur monthly after the first month of follow-up in Phase 1 and during the course of the study. During early recruitment periods of Phase 2, meetings may be cancelled if 1) data analysis has not reached an a priori adaptation point and 2) no adverse or significant adverse events occurred during the month to be reviewed. The members of the DSMB are not investigators of this study and have no conflicts of interest to disclose.

10.1.8 QUALITY ASSURANCE AND QUALITY CONTROL

Quality Assurance will occur at three levels: 1) at the research nurse level 2) at the data entry level and 3) at the data system level. First, at the research nurse level, after data collection, the project coordinator on-site at KCMC will be responsible for performing a quality check daily on data collected. He/She will mark unfilled, incomplete or nonsensical data for reevaluation by the nurse who collected the data. This will occur on a daily basis at the end of each day of data collection. Next, during data entry into our online systems, all data will be evaluated for completeness and appropriateness. Any erroneous data will be updated and we will attempt to identify potential systematic errors or process challenges in order to standardize our process to remove data entry errors. After initial data entry, our project coordinator will check 10% of the participants' data to ensure appropriate and complete data entry. Finally, at the data system level, we will have weekly quality assurance evaluations where we can determine the amount of missing data, find any responses which lie outside a priori established

validation parameters and which are outside expected range of valid responses. These data evaluations can occur weekly and provide feedback to our research team at KCMC to perform rapid quality improvement and changes in protocols as needed.

10.1.9 DATA HANDLING AND RECORD KEEPING

10.1.9.1 DATA COLLECTION AND MANAGEMENT RESPONSIBILITIES

All data (screening, enrollment and intervention logs, questionnaires/ data collection sheets, audio recordings, translations and transcriptions) will be stored by study identification number. As discussed above, all hard copy paper will be stored by study identification number in locked cabinets in a locked office of the second floor of the CCFCC. Online data will be stored by study identification number behind the Duke firewall and accessible to those included in the regulatory process.

10.1.9.2 STUDY RECORDS RETENTION

TBD

10.1.10 PROTOCOL DEVIATIONS

The study team will review protocol deviations on a weekly basis, and the PI will implement corrective actions when the quantity or nature of deviations are deemed to be at a level of concern.

Should involvement of the DSMB and/or Advisory Board become necessary, the PI will provide direct access to all trial related sites, source data/documents, and reports for the purpose of monitoring and auditing by those individuals, and, if needed, the funding agency or regulatory bodies.

10.1.11 PUBLICATION AND DATA SHARING POLICY

This study will comply with the NIH Data Sharing Policy and Policy on the Dissemination of NIH-Funded Clinical Trial Information and the Clinical Trials Registration and Results Information Submission rule. As such, this trial will be registered at ClinicalTrials.gov, and results information from this trial will be submitted to ClinicalTrials.gov. In addition, every attempt will be made to publish results in open access, peer-reviewed journals. Data from this study may be requested from other researchers 6 years after the completion of the primary endpoint by contacting Dr. Catherine A. Staton.

10.1.12 CONFLICT OF INTEREST POLICY

Therefore any actual conflict of interest of persons who have a role in the design, conduct, analysis, publication, or any aspect of this trial will be disclosed and managed. Furthermore, persons who have a perceived conflict of interest will be required to have such conflicts managed in a way that is appropriate to their participation in the design and conduct of this trial.

10.2 ADDITIONAL CONSIDERATIONS

10.3 ABBREVIATIONS AND SPECIAL TERMS

AUD: Alcohol Use Disorder
BI: Brief Intervention
ED: Emergency Department
KCMC: Kilimanjaro Christian Medical Center
LMIC: Low and Middle Income Countries
NIAAA: (United States) National Institute of Alcohol Abuse and Alcoholism
NIH: (United States) National Institute of Health
PPKAY: Punguza Pombe Kwa Afya Yako/ Reduce Alcohol for Your Health
PRACT: Pragmatic Randomized Adaptive Clinical Trial
SBIRT: Screening and Brief Intervention for Treatment
WHO: World Health Organization

10.4 PROTOCOL AMENDMENT HISTORY

Version	Date	Description of Change	Brief Rationale
	30 Nov 2020	Primary outcome changed from harms (DrInC) to binge drinking days, schema of phases changed, sample size adjusted, participant compensation modified, and DSMB updated	DSMB, Advisory Board, and study staff offered feedback suggesting all changes. Changes to sample size resulted from change of phase schema and primary outcome, plus extra consideration for attrition.
	2 Dec 2020	SF8 Quality of Life measure removed	Advisory Board suggested removal of SF8
	7 Dec 2020	PHQ-9 measure replaced instances of PHQ-2 or Kessler as depression measure. Added depression (PHQ-9) as secondary outcome where missing.	Depression measure was erroneously omitted as outcome measure in previous versions. Inconsistencies in which measure (Kessler, PHQ-9, PHQ-2 rectified.
	16 June 2023	Condensing from 3 to only 2 phases, in which Phase 2 will:<br>• compare the brief intervention (“PPKAY”) alone with PPKAY +	Slower than anticipated enrollment will not allow full enrollment as initially planned.

		standard SMS booster and PPKAY + personalized SMS booster,<br>• reduce the overall maximum sample size to 864 participants<br><br>Qualitative interview questions have been added, to be asked of a subset of enrolled participants at the 24-month follow-up visit.<br><br>Clarified that the 24-month follow-up visit is a planned part of the study activities.	

11 REFERENCES

REFERENCES

1. WHO. Global status report on alcohol and health 2018. Geneva: World Health Organization;2018.

2. Lim SS, Vos T, Flaxman AD, Danaei G, Shibuya K, Adair-Rohani H, Amann M, Anderson HR, Andrews KG, Aryee M, Atkinson C, Bacchus LJ, Bahalim AN, Balakrishnan K, Balmes J, Barker-Collo S, Baxter A, Bell ML, Blore JD, Blyth F, Bonner C, Borges G, Bourne R, Boussinesq M, Brauer M, Brooks P, Bruce NG, Brunekreef B, Bryan-Hancock C, Bucello C, Buchbinder R, Bull F, Burnett RT, Byers TE, Calabria B, Carapetis J, Carnahan E, Chafe Z, Charlson F, Chen H, Chen JS, Cheng AT, Child JC, Cohen A, Colson KE, Cowie BC, Darby S, Darling S, Davis A, Degenhardt L, Dentener F, Des Jarlais DC, Devries K, Dherani M, Ding EL, Dorsey ER, Driscoll T, Edmond K, Ali SE, Engell RE, Erwin PJ, Fahimi S, Falder G, Farzadfar F, Ferrari A, Finucane MM, Flaxman S, Fowkes FG, Freedman G, Freeman MK, Gakidou E, Ghosh S, Giovannucci E, Gmel G, Graham K, Grainger R, Grant B, Gunnell D, Gutierrez HR, Hall W, Hoek HW, Hogan A, Hosgood HD 3rd, Hoy D, Hu H, Hubbell BJ, Hutchings SJ, Ibeanusi SE, Jacklyn GL, Jasrasaria R, Jonas JB, Kan H, Kanis JA, Kassebaum N, Kawakami N, Khang YH, Khatibzadeh S, Khoo JP, Kok C, Laden F, Lalloo R, Lan Q, Lathlean T, Leasher JL, Leigh J, Li Y, Lin JK, Lipshultz SE, London S, Lozano R, Lu Y, Mak J, Malekzadeh R, Mallinger L, Marcenes W, March L, Marks R, Martin R, McGale P, McGrath J, Mehta S, Mensah GA, Merriman TR, Micha R, Michaud C, Mishra V, Mohd Hanafiah K, Mokdad AA, Morawska L, Mozaffarian D, Murphy T, Naghavi M, Neal B, Nelson PK, Nolla JM, Norman R, Olives C, Omer SB, Orchard J, Osborne R, Ostro B, Page A, Pandey KD, Parry CD, Passmore E, Patra J, Pearce N, Pelizzari PM, Petzold M, Phillips MR, Pope D, Pope CA 3rd, Powles J, Rao M, Razavi H, Rehfuess EA, Rehm JT, Ritz B, Rivara FP, Roberts T, Robinson C, Rodriguez-Portales JA, Romieu I, Room R, Rosenfeld LC, Roy A, Rushton L, Salomon JA, Sampson U, Sanchez-Riera L, Sanman E, Sapkota A, Seedat S, Shi P, Shield K, Shivakoti R, Singh GM, Sleet DA, Smith E, Smith KR, Stapelberg NJ, Steenland K, Stöckl H, Stovner LJ, Straif K, Straney L, Thurston GD, Tran JH, Van Dingenen R, van Donkelaar A, Veerman JL, Vijayakumar L, Weintraub R, Weissman MM, White RA, Whiteford H, Wiersma ST, Wilkinson JD, Williams HC, Williams W, Wilson N, Woolf AD, Yip P, Zielinski JM, Lopez AD, Murray CJ, Ezzati M, AlMazroa MA, Memish ZA, A comparative risk assessment of burden of disease and injury attributable to 67 risk factors and risk factor clusters in 21 regions, 1990-2010: a systematic analysis for the Global Burden of Disease Study 2010., Lancet. 2012 Dec 15;380(9859):2224-60

   Pmid:23245609

3. Rehm J, Rehn N, Room R, Monteiro M, Gmel G, Jernigan D, Frick U, The global distribution of average volume of alcohol consumption and patterns of drinking., Eur Addict Res. 2003 Oct;9(4):147-56

   Pmid:12970583

4. Degenhardt L, Whiteford HA, Ferrari AJ, Baxter AJ, Charlson FJ, Hall WD, Freedman G, Burstein R, Johns N, Engell RE, Flaxman A, Murray CJ, Vos T, Global burden of disease attributable to illicit drug use and dependence: findings from the Global Burden of Disease Study 2010., Lancet. 2013 Nov 9;382(9904):1564-74

   Pmid:23993281

5. Francis JM, Weiss HA, Mshana G, Baisley K, Grosskurth H, Kapiga SH, The Epidemiology of Alcohol Use and Alcohol Use Disorders among Young People in Northern Tanzania., PLoS One. 2015;10(10):e0140041

Pmid:26444441

6. Isaksen AB, Østbye T, Mmbaga BT, Daltveit AK, Alcohol consumption among pregnant women in Northern Tanzania 2000-2010: a registry-based study., BMC Pregnancy Childbirth. 2015 Sep 3;15:205

Pmid:26337194

7. Cherpitel CJ, Alcohol and injuries: a review of international emergency room studies since 1995., Drug Alcohol Rev. 2007 Mar;26(2):201-14

Pmid:17364856

8. Foran HM, O'Leary KD, Alcohol and intimate partner violence: a meta-analytic review., Clin Psychol Rev. 2008 Oct;28(7):1222-34

Pmid:18550239

9. Zaleski M, Pinsky I, Laranjeira R, Ramisetty-Mikler S, Caetano R, Intimate partner violence and alcohol consumption., Rev Saude Publica. 2010 Feb;44(1):53-9

Pmid:20140329

10. Medley A, Seth P, Pathak S, Howard AA, DeLuca N, Matiko E, Mwinyi A, Katuta F, Sheriff M, Makyao N, Wanjiku L, Ngare C, Bachanas P, Alcohol use and its association with HIV risk behaviors among a cohort of patients attending HIV clinical care in Tanzania, Kenya, and Namibia., AIDS Care. 2014;26(10):1288-97

Pmid:24773163

11. Cherpitel CH BG, Giesbrecht N, Hungerford, Peden M, Poznyak V, Room R, Stockwell T,, ed Alcoholand Injuries: Emergency Department Studies in an International Perspective. Geneva, Switzerland: WorldHealth Organization; 2009.

12. WHO. Alcohol Use and Sexual Risk Behavior: A Cross Cultural Study in Eight Countries. Geneva: World Health Organization;2005.

13. WHO. Mental Health Atlas 2014. Geneva, Switzerland: World Health Organization;2015.

14. Elzerbi C, Donoghue K, Drummond C, A comparison of the efficacy of brief interventions to reduce hazardous and harmful alcohol consumption between European and non-European countries: a systematic review and meta-analysis of randomized controlled trials., Addiction. 2015 Jul;110(7):1082-91

Pmid:25916993

15. Désy PM, Howard PK, Perhats C, Li S, Alcohol screening, brief intervention, and referral to treatment conducted by emergency nurses: an impact evaluation., J Emerg Nurs. 2010 Nov;36(6):538-45

Pmid:21078465

16. D'Onofrio G, Fiellin DA, Pantalon MV, Chawarski MC, Owens PH, Degutis LC, Busch SH, Bernstein SL, O'Connor PG, A brief intervention reduces hazardous and harmful drinking in emergency department patients., Ann Emerg Med. 2012 Aug;60(2):181-92

Pmid:22459448

17. Barbosa C, Cowell A, Bray J, Aldridge A, The Cost-effectiveness of Alcohol Screening, Brief Intervention, and Referral to Treatment (SBIRT) in Emergency and Outpatient Medical Settings., J Subst Abuse Treat. 2015 Jun;53:1-8

Pmid:25648375

18. WHO. Global status report on alcohol and health. Geneva, Switzerland: World Health Organization;2014. ISBN 978 92 4 069276 3.

19. O'Donnell A, Anderson P, Newbury-Birch D, et al. The impact of brief alcohol interventions in primary healthcare: a systematic review of reviews. Alcohol Alcohol. 2014;49(1):66-78.

20. Kaner EF, Beyer Fr Fau - Muirhead C, Muirhead C Fau - Campbell F, et al. Effectiveness of brief alcohol interventions in primary care populations. (1469-493X (Electronic)).

21. Barata IA, Shandro JR, Montgomery M, Polansky R, Sachs CJ, Duber HC, Weaver LM, Heins A, Owen HS, Josephson EB, Macias-Konstantopoulos W, Effectiveness of SBIRT for Alcohol Use Disorders in the Emergency Department: A Systematic Review., West J Emerg Med. 2017 Oct;18(6):1143-1152

Pmid:29085549

22. O'Donnell A, Anderson P, Newbury-Birch D, et al. The impact of brief alcohol interventions in primaryhealthcare: a systematic review of reviews. Alcohol Alcohol. 2014;49(1):66-78.

23. Joseph J, Basu D. Efficacy of Brief Interventions in Reducing Hazardous or Harmful Alcohol Use in Middle-Income Countries: Systematic Review of Randomized Controlled Trials. Alcohol Alcoholism. 2017;52(1):56-64.

24. Drummond C, Deluca P, Coulton S, Bland M, Cassidy P, Crawford M, Dale V, Gilvarry E, Godfrey C, Heather N, McGovern R, Myles J, Newbury-Birch D, Oyefeso A, Parrott S, Patton R, Perryman K, Phillips T, Shepherd J, Touquet R, Kaner E, The effectiveness of alcohol screening and brief intervention in emergency departments: a multicentre pragmatic cluster randomized controlled trial., PLoS One. 2014;9(6):e99463

    Pmid:24963731

25. Heather N, The efficacy-effectiveness distinction in trials of alcohol brief intervention., Addict Sci Clin Pract. 2014 Aug 18;9:13

    Pmid:25127717

26. Takahashi R, Wilunda C, Magutah K, Mwaura-Tenambergen W, Atwoli L, Perngparn U, Evaluation of Alcohol Screening and Community-Based Brief Interventions in Rural Western Kenya: A Quasi-Experimental Study., Alcohol Alcohol. 2018 Jan 1;53(1):121-128

    Pmid:29087434

27. Myers B, Sorsdahl K, Morojele NK, Kekwaletswe C, Shuper PA, Parry CD, "In this thing I have everything I need": perceived acceptability of a brief alcohol-focused intervention for people living with HIV., AIDS Care. 2017 Feb;29(2):209-213

    Pmid:27435957

28. Wandera B, Tumwesigye NM, Nankabirwa JI, Mafigiri DK, Parkes-Ratanshi RM, Kapiga S, Hahn J, Sethi AK, Efficacy of a Single, Brief Alcohol Reduction Intervention among Men and Women Living with HIV/AIDS and Using Alcohol in Kampala, Uganda: A Randomized Trial., J Int Assoc Provid AIDS Care. 2017 May/Jun;16(3):276-285

    Pmid:27215561

29. Pitpitan EV, Kalichman SC, Garcia RL, Cain D, Eaton LA, Simbayi LC, Mediators of behavior change resulting from a sexual risk reduction intervention for STI patients, Cape Town, South Africa., J Behav Med. 2015 Apr;38(2):194-203

    Pmid:25179265

30. Pengpid S, Peltzer K, van der Heever H, Skaal L, Screening and brief interventions for hazardous and harmful alcohol use among university students in South Africa: results from a randomized controlled trial., Int J Environ Res Public Health. 2013 May 21;10(5):2043-57

    Pmid:23698697

31. Peltzer K, Matseke G, Azwihangwisi M, Evaluation of alcohol screening and brief intervention in routine practice of primary care nurses in Vhembe district, South Africa., Croat Med J. 2008 Jun;49(3):392-401

Pmid:18581618

32. Peltzer K, Naidoo P, Louw J, Matseke G, Zuma K, McHunu G, Tutshana B, Mabaso M, Screening and brief interventions for hazardous and harmful alcohol use among patients with active tuberculosis attending primary public care clinics in South Africa: results from a cluster randomized controlled trial., BMC Public Health. 2013 Jul 31;13:699

Pmid:23902931

33. Sorsdahl K, Petersen Williams P, Everett-Murphy K, Vythilingum B, de Villiers P, Myers B, Stein DJ, Feasibility and Preliminary Responses to a Screening and Brief Intervention Program for Maternal Mental Disorders Within the Context of Primary Care., Community Ment Health J. 2015 Nov;51(8):962-9

Pmid:25744699

34. Mertens JR, Ward CL, Bresick GF, Broder T, Weisner CM, Effectiveness of nurse-practitioner-delivered brief motivational intervention for young adult alcohol and drug use in primary care in South Africa: a randomized clinical trial., Alcohol Alcohol. 2014 Jul-Aug;49(4):430-8

Pmid:24899076

35. Sorsdahl K, Stein DJ, Corrigall J, Cuijpers P, Smits N, Naledi T, Myers B, The efficacy of a blended motivational interviewing and problem solving therapy intervention to reduce substance use among patients presenting for emergency services in South Africa: A randomized controlled trial., Subst Abuse Treat Prev Policy. 2015 Nov 14;10:46

Pmid:26576946

36. Myers B, Stein DJ, Mtukushe B, Sorsdahl K, Feasibility and Acceptability of Screening and Brief Interventions to Address Alcohol and Other Drug Use among Patients Presenting for Emergency Services in Cape Town, South Africa., Adv Prev Med. 2012;2012:569153

Pmid:23198159

37. Sorsdahl K, Stein DJ, Naledi T, Breuer E, Myers B, Problematic alcohol and other substance use among patients presenting to emergency services in South Africa: Who is ready for change?, S Afr Med J. 2017 Mar 29;107(4):352-353

Pmid:28395690

38. Benegal V, Chand PK, Obot IS, Packages of care for alcohol use disorders in low- and middle-income countries., PLoS Med. 2009 Oct;6(10):e1000170

    Pmid:19859536

39. Perngparn U, Assanangkornchai S, Pilley C, Aramrattana A. Drug and alcohol services in middle-income countries. Curr Opin Psychiatry. 2008;21(3):229-233.

40. Kohn R, Saxena S, Levav I, Saraceno B, The treatment gap in mental health care., Bull World Health Organ. 2004 Nov;82(11):858-66

    Pmid:15640922

41. Staton C. Addressing High Risk Alcohol Use among Injury Patients in an Emergency Department inTanzania. Moshi, Tanzania: Fogarty International Center; 2015-2019.

42. ACEP. Alcohol Screening and Brief Intervention in the Emergency Department: Alcohol Screening Kit Overview. https://www.acep.org/globalassets/uploads/uploaded-files/acep/clinical-and-practicemanagement/resources/publichealth/alcohol-screening/alcohol_screening_kit_overview.pdf. Accessed February 27, 2019.

43. Platt L, Melendez-Torres GJ, O'Donnell A, Bradley J, Newbury-Birch D, Kaner E, Ashton C, How effective are brief interventions in reducing alcohol consumption: do the setting, practitioner group and content matter? Findings from a systematic review and metaregression analysis., BMJ Open. 2016 Aug 11;6(8):e011473

    Pmid:27515753
