# Supplementary material for: Effectiveness of a brief intervention and text-based booster in the emergency department to reduce harmful and hazardous alcohol use: A pragmatic randomized adaptive clinical trial in Moshi, Tanzania
Source: PLoS Med. 2025 Oct 27;22(10):e1004548. doi: 10.1371/journal.pmed.1004548 (PMC12578324; doi:10.1371/journal.pmed.1004548)
Supplement: S3 File — (DOCX) [file pmed.1004548.s003.docx]

**Supplementary File S3 - Statistical Supplement - Sample size, periodic assessments and citations for R packages used**

**Sample Size and Periodic Assessments**

An effective intervention for Stage 1 was defined as having an effect size of at least 35% difference in the reduction in the primary outcome, between control and intervention arms, according to previous literature [^1–4^](https://www.zotero.org/google-docs/?HK3ROg). A 35% difference, based on average binge drinking reported in prior trials, refers to a reduction of 1.2 binge drinking days per month. The study design was estimated to have 80% power at 5% significance to detect a predicted mean difference of 1.2 in the primary end point with 164 participants. Our total planned sample was then inflated to a conservative 80% rate of retention. We pre-specified up to three periodic assessments at 25, 70 and 100% enrollment. We adjusted our sample for the adaptive periodic assessments using the O'Brien–Fleming alpha spending function. The stopping rule was defined as obtaining an effect size above pre-specified boundaries for superiority or futility.

During the second assessment, we observed an effect that was consistent with our criteria for stopping enrollment for superiority, thus our results report the analysis conducted on April 13, 2023, and reviewed during our DSMB meeting. Only the data presented in this report was unblinded to the research team. Allocation to each of the intervention arms are kept blinded for the next stages of the trial.

**Modelling**

We used a constrained longitudinal modeling approach to estimate the effect of the intervention on primary and secondary outcomes over time. For the primary outcome of binge drinking days, we fit a mixed-effects, zero-inflated negative binomial model using the glmmTMB package to account for overdispersion and excess zeros. A model matrix approach was used to explicitly select the indicator for follow-up at 3 months and the interaction between follow-up and intervention, with baseline serving as the reference timepoint. The main effect of treatment was omitted, constraining baseline means to be equal across groups. A random intercept for participant (practid) was included to account for within-subject correlation. The zero-inflation component modeled the probability of excess zeros as a function of treatment group (treat).

A similar modeling framework was applied to the secondary drinking outcomes. Importantly, no zero-inflation was applied to the secondary drinking outcomes

- Drinking days: Modeled using a mixed-effects negative binomial model. The model included fixed effects for an indicator of follow-up (period3mo) and its interaction with treatment (period3mo:treatIntervention), with baseline serving as the reference timepoint. The main effect of treatment was omitted, constraining baseline means to be equal across groups. A random intercept for participant (practid) was included to account for within-subject correlation
- Drinking amount: Modeled using a mixed-effects negative binomial model with the same structure as above.

For continuous secondary outcomes including DrInC total score, AUDIT total score, and PHQ-9, we used linear mixed-effects models (lmer from the lmerTest package). The models included fixed effects for an indicator of follow-up (period3mo) and its interaction with treatment (period3mo:treatIntervention), with baseline serving as the reference timepoint. The main effect of treatment was omitted, constraining baseline means to be equal across groups with random intercepts for each participant (practid) to account for clustering.

No other covariates were used in the modelling.

To estimate treatment effects across outcomes, we applied an approach using a non-parametric bootstrap resampling within a multiple imputation framework (see Multiple Imputation by Chained Equations below). For each imputed dataset, we fit the prespecified model (described above) to generate predicted outcome values for each individual. We then calculated within-group differences from baseline to follow-up and used bootstrap resampling (1,000 iterations per imputation) to estimate the distribution of these changes separately for each treatment group. To estimate the treatment effect, we computed the difference in these within-group changes (i.e., the difference-in-differences) using the same bootstrap procedure. This process was repeated across all imputed datasets, and the resulting bootstrap replicates were pooled across imputations. Final point estimates were calculated as the mean of the pooled bootstrap samples, and 95% confidence intervals were obtained using the 2.5th and 97.5th percentiles of the empirical bootstrap distributions. This approach was applied consistently across all primary and secondary outcomes.

Complete case analysis followed the same logic, but in the single original dataset.

Please refer to our GitHub repository (https://github.com/gemini-duke/PRACT-3Months) for a full version of our analysis codes.

**Multiple imputation by chained equations**

To address missingness, we implemented multiple imputation by chained equations using the mice package in R. Imputation of outcomes was performed using predictive mean matching (PMM), a semi-parametric method that retains the distributional properties of observed data by drawing from actual observed values of similar cases rather than generating synthetic ones. For each outcome, 100 imputed datasets were created. The imputation algorithm was run for up to 50 iterations per dataset following 100 burn-in iterations to ensure stability and convergence. Final analyses were performed across all imputed datasets, and estimates were combined using Rubin’s rules. Each outcome was imputed separately, using a long-format dataset that combined baseline and 3-month observations. For each imputation model, covariates included age, sex, tribe, employment status, marital status, living arrangement, insurance status, individual income, household income, and education level - hereafter referred to as “demographics set”. More detail on how the variables were collected is available in the Supplementary File S7.

In addition, the imputation of each outcome variable included the individual items that composed the final summed score as well as the total score itself. In detail:

- Binge Drinking Days: demographics set + 28 variables from the 28-item Timeline Follow-Back questionnaire representing the number standard drinks consumed in the last 28 days + the corresponding summary measure (i.e. number of binge drinking days in the last 28 days);
- Drinking Days: demographics set + 28 variables from the 28-item Timeline Follow-Back questionnaire representing the number standard drinks consumed in the last 28 days + the corresponding summary measure (i.e. number of drinking days in the last 28 days);
- Drinking Amount: demographics set + 28 variables from the 28-item Timeline Follow-Back questionnaire representing the number standard drinks consumed in the last 28 days + the corresponding summary measure (i.e. number of drinks consumed in the last 28 days);
- AUDIT: demographics set + the 10 individual AUDIT items in addition to the total AUDIT score.
- DrInC: demographics set + the 50 DrInC item-level responses were included, along with the total DrInC score.
- PHQ-9: demographics set + the 9 individual PHQ-9 item responses and the total PHQ-9 score.

**R packages used in analysis/writing**

rpact [^5^](https://www.zotero.org/google-docs/?ikAH0k) - sample size calculations and power estimations at periodic assessments;

lmerTest[^6^](https://www.zotero.org/google-docs/?b88WEk), glmmTMB[^7^](https://www.zotero.org/google-docs/?faW6Fe), lme4[^8^](https://www.zotero.org/google-docs/?N9nbFV) - statistical modeling;

gtsummary [^9^](https://www.zotero.org/google-docs/?xSvy4f), gt [^10^](https://www.zotero.org/google-docs/?xme9sl) - tables and statistical summaries;

tidyverse [^11^](https://www.zotero.org/google-docs/?OLrPc7) - data wrangling, plotting;

lubridate [^12^](https://www.zotero.org/google-docs/?DRpysV) - working with dates,

redcapAPI [^13^](https://www.zotero.org/google-docs/?sTAR4a) - downloading data from REDCap;

stringr [^14^](https://www.zotero.org/google-docs/?edPgZC) - working with strings;

ggh4x [^15^](https://www.zotero.org/google-docs/?Jh3pT4), qwraps2 [^16^](https://www.zotero.org/google-docs/?7JV0Ys), ggsignif [^17^](https://www.zotero.org/google-docs/?oMejmO) - extensions to ggplot plots;

**References**

[1 Elzerbi C, Donoghue K, Drummond C. A comparison of the efficacy of brief interventions to reduce hazardous and harmful alcohol consumption between European and non-European countries: a systematic review and meta-analysis of randomized controlled trials. *Addiction* 2015; **110**: 1082–91.](https://www.zotero.org/google-docs/?Vhjb7L)

[2 Cherpitel CJ, Ye Y, Moskalewicz J, Świątkiewicz G. Does brief intervention work for heavy episodic drinking? A comparison of emergency department patients in two cultures. *Alcoholism and Drug Addiction* 2015; **28**: 145–62.](https://www.zotero.org/google-docs/?Vhjb7L)

[3 Schmidt CS, Schulte B, Seo H-N, *et al.* Meta-analysis on the effectiveness of alcohol screening with brief interventions for patients in emergency care settings. *Addiction* 2016; **111**: 783–94.](https://www.zotero.org/google-docs/?Vhjb7L)

[4 Woolard R, Baird J, Longabaugh R, *et al.* Project Reduce: Reducing alcohol and marijuana misuse: Effects of a brief intervention in the emergency department. *Addictive Behaviors* 2013; **38**: 1732–9.](https://www.zotero.org/google-docs/?Vhjb7L)

[5 Wassmer G, Pahlke F, Jensen T, Schueuerhuis S. rpact: Confirmatory Adaptive Clinical Trial Design and Analysis. 2023; published online July 3. https://cran.r-project.org/web/packages/rpact/index.html (accessed Nov 11, 2023).](https://www.zotero.org/google-docs/?Vhjb7L)

[6 Kuznetsova A, Brockhoff PB, Christensen RHB. lmerTest Package: Tests in Linear Mixed Effects Models. *Journal of Statistical Software* 2017; **82**: 1–26.](https://www.zotero.org/google-docs/?Vhjb7L)

[7 Brooks ME, Kristensen K, Benthem KJ van, *et al.* glmmTMB Balances Speed and Flexibility Among Packages for Zero-inflated Generalized Linear Mixed Modeling. *The R Journal* 2017; **9**: 378–400.](https://www.zotero.org/google-docs/?Vhjb7L)

[8 Bates D, Mächler M, Bolker B, Walker S. Fitting Linear Mixed-Effects Models Using lme4. *Journal of Statistical Software* 2015; **67**: 1–48.](https://www.zotero.org/google-docs/?Vhjb7L)

[9 Sjoberg DD, Whiting K, Curry M, Lavery JA, Larmarange J. Reproducible Summary Tables with the gtsummary Package. *The R Journal* 2021; **13**: 570–80.](https://www.zotero.org/google-docs/?Vhjb7L)

[10 Iannone R, Cheng J, Schloerke B, Hughes E, Lauer A, Seo J. gt: Easily Create Presentation-Ready Display Tables. 2023 https://CRAN.R-project.org/package=gt.](https://www.zotero.org/google-docs/?Vhjb7L)

[11 Wickham H, Averick M, Bryan J, *et al.* Welcome to the Tidyverse. *Journal of Open Source Software* 2019; **4**: 1686.](https://www.zotero.org/google-docs/?Vhjb7L)

[12 Grolemund G, Wickham H. Dates and Times Made Easy with lubridate. *Journal of Statistical Software* 2011; **40**: 1–25.](https://www.zotero.org/google-docs/?Vhjb7L)

[13 Benjamin, Beasley W, jeffreyhorner. redcapAPI v1.0. 2014; published online Sept 22. DOI:10.5281/zenodo.11826.](https://www.zotero.org/google-docs/?Vhjb7L)

[14 Wickham H. stringr: Simple, Consistent Wrappers for Common String Operations. 2022 https://CRAN.R-project.org/package=stringr.](https://www.zotero.org/google-docs/?Vhjb7L)

[15 Brand T van den. ggh4x: Hacks for ‘ggplot2’. 2023 https://CRAN.R-project.org/package=ggh4x.](https://www.zotero.org/google-docs/?Vhjb7L)

[16 DeWitt P. qwraps2: Quick Wraps 2. 2021 https://CRAN.R-project.org/package=qwraps2.](https://www.zotero.org/google-docs/?Vhjb7L)

[17 Constantin A-E, Patil I. ggsignif: R Package for Displaying Significance Brackets for ‘ggplot2’. *PsyArxiv* 2021. DOI:10.31234/osf.io/7awm6.](https://www.zotero.org/google-docs/?Vhjb7L)
