# Supplementary material for: Effectiveness of a brief intervention and text-based booster in the emergency department to reduce harmful and hazardous alcohol use: A pragmatic randomized adaptive clinical trial in Moshi, Tanzania
Source: PLoS Med. 2025 Oct 27;22(10):e1004548. doi: 10.1371/journal.pmed.1004548 (PMC12578324; doi:10.1371/journal.pmed.1004548)
Supplement: S4 File — (PDF) [file pmed.1004548.s004.pdf]

[redacted]		*Version: DSP 1*<br>*Effective Date: 07AUG19*
NIAAA Data Archive (NIAAA$_{DA}$) Data Sharing Plan (DSP)		
PI Name: Catherine Staton	Grant Number: R01AA027512	
Approval Date:		

NIAAA Data Archive (NIAAA$_{DA}$) Data Sharing Plan (DSP) Template Instructions

Per NOT-AA-19-020, the National Institute on Alcohol Abuse and Alcoholism (NIAAA) expects investigators with applicable grant applications to include plans for sharing NIAAA-funded human subjects grant-related data with the NIAAA Data Archive (NIAAA$_{DA}$). For this purpose, **NIAAA strongly encourages that investigators complete and include the following NIAAA$_{DA}$ Data Sharing Plan (DSP) template in the Resource Sharing Plan section of grant applications.** This DSP includes a summary tasks and expectations related to submitting data to the NIAAA$_{DA}$.

Alterations to this DSP template are generally not encouraged (except where grant-specific information is queried); however, if any alterations are proposed, they must be accompanied by a justification as to why fulfillment of the tasks and expectations are not possible. This justification will be reviewed by the NIAAA Program Officer assigned to the grant and may require further justification/alteration to be approved by the Program Officer.

More detailed information related to investigator tasks and expectations can be found in the NIAAA$_{DA}$ website.

NIAAA Data Archive ($NIAAA_{DA}$) Data Sharing Plan (DSP)

Per NOT-AA-19-020, this study will submit and share data with NIAAA Data Archive ($NIAAA_{DA}$), a data repository housed within the NIMH Data Archive (NDA). To this end, this study will fulfill the following summary of tasks and expectations:

- Obtain Informed Consent that allows for broad sharing of the research subjects' de-identified data.
- Collect Personally Identifiable Information (PII) from research subjects that will allow for the creation of the NDA GUID (a de-identified subject ID).
- Create an NDA Account with access to the GUID Tool.
- Complete and submit a Data Submission Agreement (DSA) within 6 months of the Notice of Award (NoA) issue date.
- Create a list of data items to be collected in the project in the Data Expected tab of the NDA Collection within 6 months of NoA issue date.
- Create a GUID for each research subject using the NDA GUID tool.
- Format and submit data according to the NDA Data Dictionary and/or work with NDA staff to define an appropriate data structure, if one does not already exist.
- Submit data on or before the NDA submission due dates (April 1 and October 1 each year) in accordance with the applicable Data Sharing Terms and Conditions of award.
- Perform QA/QC checks on data within 4 months after the submission due dates and address any issues identified by the NDA.
- Share data in accordance with the default $NIAAA_{DA}$ data sharing schedule in accordance with the applicable Data Sharing Terms and Conditions of award.
- Upload supporting documents that allow future analysts to effectively use the data, including: study protocols, assessment schedules, operating procedures, original assessment instruments (if not proprietary), and analytic/statistical algorithms used to derive variables in publications.

(continued next page)

- Create an NDA Study, linking relevant publications to submitted data at the time of publication, and use the appropriate Digital Object Identifier (DOI) and Acknowledgement template in these publications.

- Submit a statement of progress on data sharing in non-competing renewals and progress reports. Further instructions are available in the RPPR Instruction guide under section C.5.b. Resource Sharing. Refer to specific Tutorials about data submission and sharing via the NDA or refer to the data sharing planning section where the data will be shared.

Grant-specific details (to be completed by applicant):

1. Brief summary of the assessment schedule for data that will eventually be shared with the NIAAA$_{DA}$[1]:

In this pragmatic randomized controlled trial, adult (>18 years) injury patients presenting for an acute injury (<24 hours) will be enrolled if they 1) have a positive breathalyzer 2) have an Alcohol Use Disorder Identification Tool assessment ≥8 or 3) admit to alcohol use prior to the injury. Participants will be allocated to one of four arms depending on the trial phase, usual care, brief intervention, brief intervention with standard booster or brief intervention with personalized booster. At 3, 6, 9, 12 and 24 months, we evaluate clinical effectiveness with assessments of alcohol and biopsychosocial measures.

2. Listing of proposed data collection instruments (assessments) that do not currently exist in the NDA (if applicable)[2]:
   a. Drinker Inventory of Consequences (DrInC)
   b. Types of alcohol used (distilled, beer…)
   c. Alcohol attributed initial event
   d. Readiness to change
   e. Patient Specific Functional Scale
   f. Short form health survey 8 items version (SF-8)
   g. Coping Inventory for Stressful Situations (CISS-21)
   h. East Africa Alcohol Expectancy Scale (AFEXS)
   i. Treatment Self-Regulation Questionnaire
   j. Situational Confidence Questionnaire

[1] A sample summary for illustrative purposes: *"In this 6-week clinical trial, clinical assessments to establish baseline subject characteristics and eligibility criteria will be collected from all research participants at the screening and randomization visits, prior to the administration of study medication. Clinical efficacy and safety assessments will continue for each in-clinic visit (Weeks 1, 2, 4, 6) and during telephone screenings (Weeks 3 and 5). Structural and functional MRI scans will be collected from half of the participants during Week 3. At post-treatment follow-up visits (Weeks 12, 24, 52), clinical efficacy and safety assessments will be repeated to assess long-term effect of study medication, well as patient satisfaction with the treatment."*

[2] A list of existing data collection instruments (data dictionaries) can be found at https://nda.nih.gov/data_dictionary.html?source=NDA&submission=ALL. Whenever feasible and/or appropriate, NIAAA strongly encourages awardees to use existing data collection instruments rather the create new ones. It is understood that this listing of proposed instruments that are not currently in the NDA may change in the early development phase of the grant as instrument selection is finalized.

k. Perceived Alcohol Stigma
l. Household Food Insecurity Access Scale (HFIAS)
m. Self-reported perceived seriousness of event

3. Proposed schedule for running the data validation tool once data collection begins[3]:

In accordance with our data quality assurance plan, we will conduct data validation tool weekly.

[3] The data validation tool is a web-based application that runs quality assurance checks on data submitted to the $NIAAA_{DA}$. The tool ensures that the submitted data are consistent with expected data definitions in pre-defined data dictionaries. At a minimum, the validation tool must be run twice a year for the biannual data submission dates. However, as the tool can be run anytime during the data collection phase of the study, **NIAAA strongly recommends applicants propose a more frequent running of the tool (e.g., daily or once per week)** to ensure the rigor and reproducibility of their studies.

[redacted]		*Version: DSP 1*
		Effective Date: 07AUG19

NIAAA Data Archive (NIAAA$_{DA}$) Data Sharing Plan (DSP)	
PI Name: Catherine Staton	Grant Number: R01AA027512
Approval Date:	

The DSP must be signed by the Principal Investigator(s) (PI), Authorized Organization Representative (AOR), and the NIAAA Program Officer:

Signatures:

_______________________________ *17 Dec 2019*

Catherine Staton, PI Date

_______________________________ _______________

<Name> PI Date

Carol P. Smith, MBA for John Michnowicz Digitally signed by Carol P. Smith, MBA for John Michnowicz Date: 2019.12.19 10:13:20 -05'00'

_______________________________ _______________

<Name> AOR Date

_______________________________ _______________

<Name> NIAAA PO Date
