# Supplementary material for: Effectiveness of a brief intervention and text-based booster in the emergency department to reduce harmful and hazardous alcohol use: A pragmatic randomized adaptive clinical trial in Moshi, Tanzania
Source: PLoS Med. 2025 Oct 27;22(10):e1004548. doi: 10.1371/journal.pmed.1004548 (PMC12578324; doi:10.1371/journal.pmed.1004548)
Supplement: S5 File — (DOCX) [file pmed.1004548.s005.docx]

**Supplementary File S5: Statistical Analysis Plan**

Statistical Analysis Plan

**PRACT: A Pragmatic Randomized Adaptive Clinical Trial to Investigate Controlling Alcohol-related Harms in a Low-Income Setting; Emergency Department Brief Interventions in Tanzania**

SAP Version: 2

SAP Date: September 15th, 2020

Revision Date(s): September 12, 2022

Authors:

Principal Investigator Approval: Catherine Staton

Analysis team: Linda Minja, Timothy Antipas Peter, Joao Vitor Perez de Souza, John Gallis, Joao Ricardo N. Vissoci

Advisor: Gail Donofrio, Michael Pantalon, Brian Suffoletto, Monica Swahn, John Bartlett, Kristine Broglio, Jon Mark Hirshon, Judith Boshe, Emanueli Msuya

Revisions/Notes:

# 1 Study Hypotheses and Outcomes

# Background

Alcohol-related health consequences, in Africa and specifically Tanzania, are increasing; especially where there are severely limited treatment options. Nearly one-third of all deaths attributed to alcohol globally are caused by injuries. More specifically, in the Africa region, drinking patterns are the second worst worldwide with high rates of binge drinking and alcohol dependence. In Tanzania, 11.5% of men and 6.8% of the overall population were estimated to have an AUD, with 33.4% of males having heavy episodic drinking. Within Tanzania, the Kilimanjaro region’s scarce literature has shown very high rates of AUD, especially among high-risk groups. Both chronic and heavy episodic alcohol use have been associated with many high-risk behaviors: crime, aggressive driving, HIV risk behaviors, interpersonal violence, unintentional injuries, and self-inflicted injury. As such, our team has created and piloted “Punguza Pombe Kwa Afya Yako” (PPKAY)/ “Reduce Alcohol for Your Health”; a brief intervention for the KCMC ED. This is a one-time, 15-minute, nurse-led intervention based on motivational principles, which discusses safe drinking behaviors, and negotiates change in alcohol use. In our trial, we will compare PPKAY to the usual care with no alcohol harm prevention education.

# Primary Hypothesis

*Stage 1.* Primary hypothesis that randomization to the intervention (PPKAY + Personalized Text Booster or PPKAY + Standard Text Booster) arms will result in reduction in # of binge drinking days per month up to 3 months after hospital discharge, relative to those in the Usual Care arm.

- 1. **Secondary Hypothesis**

*Stage 2.* Randomization to the PPKAY + Standard Text Booster intervention arm is non-inferior than the PPKAY + Personalized Text Booster in reducing the # of binge drinking days per month up to 3 months after hospital discharge.

*Stage 3.* Randomization to the PPKAY w/o Text Booster intervention arm is non-inferior to the PPKAY + Text Booster (Standard or Personalized, winner on Stage 2) in reducing the # of binge drinking days per month up to 3 months after hospital discharge.

# 1.3 Primary Outcomes

**Table 1. Primary Outcomes**

| **Outcome** | **Description** | **Variables and Source** |
| --- | --- | --- |
| Number of binge drinking days | Average change in the number of binge drinking days in the past four weeks | TLFB |

# 1.4 Secondary Outcomes

**Table 2. Secondary Outcomes**

| **Outcome** | **Description** | **Variables and Source** |
| --- | --- | --- |
| Frequency of drinking days | Average change in the number of drinking days in the past four weeks | TLFB |
| Quantity of alcohol use | Average change in the amounts of alcohol consumed in the past two weeks | TLFB |
| Alcohol-related harm s | Average change in the number of alcohol related consequences | DrInC |
| Alcohol dependence | Average change in the AUDIT scores | AUDIT |
| Depression | Average change in the PHQ-9 scores | PHQ-9 |

**1.5. Study design**

This study had been designed as an adaptive randomized control trial with 3 distinct stages (Figure 1). Stage 1 of our PRACT is a superiority trial comparing usual care to receiving PPKAY + Text Booster (Standard or Personalized). We will have three arms (Usual care, PPKAY + Standard Text Booster or PPKAY + Personalized Text Booster) using a 1:1:1 allocation with a 12-block randomization. Hypothesis will be tested comparing Usual care to a pooled intervention arm aggregating both types of PPKAY + Text Boosters.

Stage 2 will be a non-inferiority trial of PPKAY + Text Booster compared to PPKAY without Text Boosters. At this stage of our PRACT, we will carry on the patients enrolled in the intervention arms during Stage 1 and will start to enroll in a 1:1:4 allocation, a new arm with a PPKAY w/o Text Booster, using a 12-block randomization. Completion of our trial will occur when 1) we find statistical significance of one of the final intervention arms or 2) we have found no statistical significance with a maximum sample size.


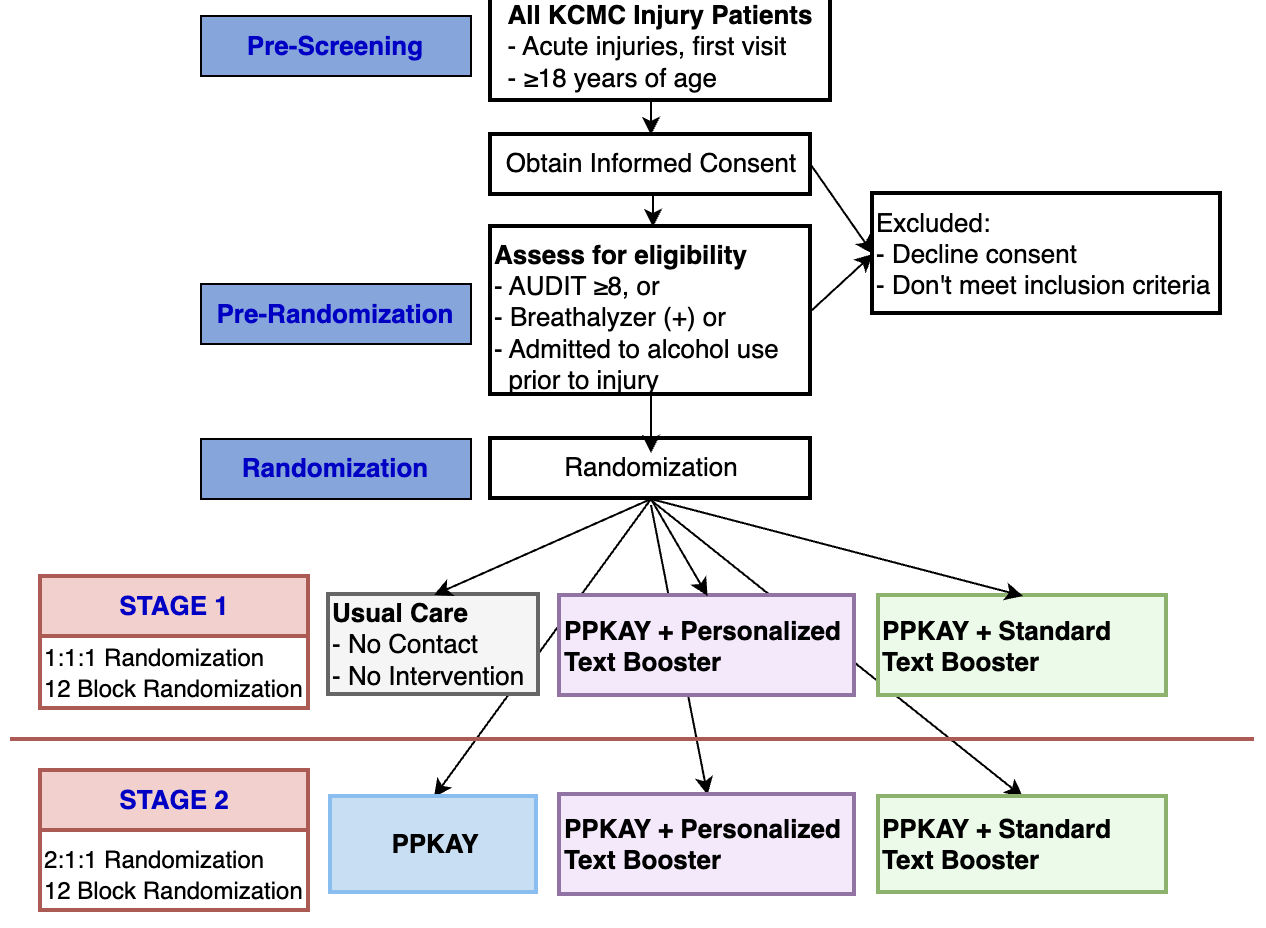


**Figure 1. PRACT study design**

# 2 Analysis Populations

**2.1 Description**

Data will be described with descriptive statistics with means and standard deviations. Tables 4 and 5 show the skeleton for the descriptive presentation of the results. Primary analysis of outcomes will include all participants randomized in study arms (ITT) and missing data will be handled by multiple imputation. Sensitivity analysis will be conducted to check the effect of missing data on results.

# 2.2 Sample size

*Stage 1:* An effective intervention for Stage 1 will be defined as having an effect size of at least 35% difference in the reduction in the primary outcome (number of binge drinking events per month), between control and intervention arms. Assuming an average of four binge drinking days per month (SD = 3.4), a 35% difference translates into a difference of about 1.2 days in the reduction in the number of binge drinking days per month. Evidence from studies in high-income settings suggests that studies similar to Stage 1 have shown moderate to high treatment effects of 20 - 40% in the difference. A sample size of 205 patients per arm will have 80% power to be able to identify the best study arm with a significance of 5%, and a conservative 80% retention rate.

*Stage 2*: Stage 2 is defined as a non-inferiority trial of PPKAY without SMS-Booster the PPKAY with SMS-Booster arms. A sample size of 202 participants per arm will have 80% power to identify an inferiority margin of one binge drinking day per month, with 2.5% significance and a standard deviation of 3, and a conservative 80% retention rate. Participants randomized to either booster arm in Stage 1 will be carried over to Stage 2.

Sample size calculations are attached to the end of this document using reproducible codes using R Language for Statistical Computing, v. 4.2.

**2.3 Interim Analyses and Adaptations:** In Stages 1 and 2, up to 3 interim analyses will be conducted after the first batch of patients complete 3 months of follow-up. At interim analyses, the PRACT will 1) continue the enrollment, 2) adapt to another Stage according to predefined criteria, or 3) end the study for success or futility. Success in Stage 1 will be defined as the randomization to the intervention arm (PPKAY + Personalized Text Booster or PPKAY + Standard Text Booster) demonstrating superiority to Usual Care in the difference in reduction of the number of binge drinking days per month, with 80% power to identify the necessary effect at interim analysis. If a stopping rule is reached, we will progress to Stage 2 and stop enrollment in UC. Futility in Stage 1 will be defined if no statistical difference is detected after maximum enrollment (N=205 per arm) in Stage 1. If that happens, the study will progress to Stage 3, comparing UC with the PPKAY w/o Text Booster.

Assessing success in Stage 2 will be defined in non-inferiority evaluation of PPKAY against PPKAY + Text Booster.

**2.4 Adaptive Sample Size:** The sample size was calculated for a change in the primary outcome of reduction in the number binge drinking days per month. Depending on the effect size found at each interim analysis, it is possible that significance could be reached earlier than expected. For Stage 1, recent literature supports effect sizes of 60% decrease. Additionally, there is no intervention currently available for alcohol use reduction post injury in Tanzania. Through adaptive sample re-estimation and the most effective scenario, total minimum enrollment could be as low as 520 patients. Hypothesis testing was adjusted at interim analysis according to the O'Brien-Fleming alpha-spending function. Thus, instead of spreading the alpha equally across interim analysis, the distribution is progressive leaving most of the significance levels later on the trial, when more patients are enrolled. If challenges arise with the adaptive nature of this trial, we will resort to standard sequential RCTs with maximum enrollment numbers. If standard RCT enrollment is used, an adequate sample size will be obtained to show the same effect size with higher power.

**Table 3. Maximum enrollment numbers**

**
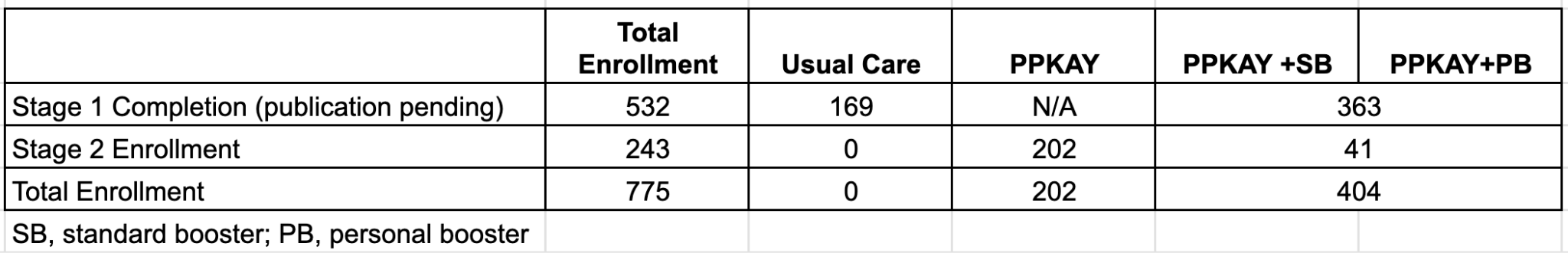
**

# 3 Statistical Methods

Patient demographics will be collected and compared across all arms of the study. Descriptive data will be reported as means, standard deviations, and frequencies. Outcome data will be reported as predicted means with confidence intervals for each follow up time point (3, 6, and 12 months), depicting the optimal intervention at each phase. Missing data will be handled by inverse probability weighting design with sequential sensitivity analysis to evaluate impact on the effect of the trial.

Preliminary analysis will be conducted to evaluate differences at baseline and differences in attrition among all study arms. These comparisons will be made using Student's t-test for continuous variables or Mann-Whitney U test according to the data distribution, and the Pearson chi-squared test for categorical variables. We will test the primary hypothesis of superior efficacy of the intervention arm in Stage 1 in relation to the control group using a longitudinal constrained approach (LCA), considering the outcome is the difference in the difference between baseline and follow-up[(1)](https://www.zotero.org/google-docs/?05y9Fg). This approach allows for the prediction of follow-up scores after controlling for participants’ baseline characteristics and other potential confounders (potential predictors of attrition and outcome confounders). Our previous observational data had shown that alcohol consumption data is overdispersed and inflated with the presence of zeros, and therefore we will use a zero-inflated negative binomial method with a log link function to model count outcomes. For AUDIT scores, depression, and alcohol related harms, linear models will be used.

# 4 Example tables and Figures

| **Table 4. Groupings** | | | |  |  |
| --- | --- | --- | --- | --- | --- |
|  | Overall | UC | PPKAY | PPKAY +standard texts | PPKAY + personalized texts |
| Total - no. (%) | xx.(100) | xx.(YY) | xx.(YY) | xx.(YY) | xx.(YY) |
| Continuous variable |  |  |  |  |  |
| Mean (SD) |  |  |  |  |  |
| Median (IQR) |  |  |  |  |  |
| Categorical Variable - no. (%) |  |  |  |  |  |
| Level 1 Row/Column Percentage |  |  |  |  |  |
| Level 2 Row/Column Percentage |  |  |  |  |  |

**Table 5. Regression Analysis**

| **Variable** | **Estimate (95% CI)** | **P-value** |
| --- | --- | --- |
| **Study Arm** |  |  |
| PPKAY + Booster | X.XXX (X.XXX, X.XXX) | 0.XXXX |
| **Time** |  |  |
| 3 months (vs. baseline) | X.XXX (X.XXX, X.XXX) | 0.XXXX |
| **Time × Study Arm Interaction** |  |  |
| 3 months × PPKAY | X.XXX (X.XXX, X.XXX) | 0.XXXX |
