# Supplementary material for: Effectiveness of a brief intervention and text-based booster in the emergency department to reduce harmful and hazardous alcohol use: A pragmatic randomized adaptive clinical trial in Moshi, Tanzania
Source: PLoS Med. 2025 Oct 27;22(10):e1004548. doi: 10.1371/journal.pmed.1004548 (PMC12578324; doi:10.1371/journal.pmed.1004548)
Supplement: S8 File — (DOCX) [file pmed.1004548.s008.docx]

# Supplementary File S8: Metadata document for the paper “Effectiveness of a Brief Negotiational Intervention and Text Based Booster to Reduce Harmful and Hazardous Alcohol Use in the Emergency Department of a Low Resource Setting: A Pragmatic Randomized Adaptive Clinical Trial in Moshi, Tanzania”

##

## Dataset Version

April 2023

##

## Data Collection Method

Paper forms, REDCap

##

## Data Cleaning

Numerical integers were substituted with their corresponding labels, as outlined in this document, whenever applicable. For the purpose of generating scores, relevant variables were summed up. Importantly, our data analysis was conducted strictly on complete cases; we did not employ any data imputation methods for filling in missing values. Any missing data points are explicitly represented in the descriptive tables accompanying the study.

## Code Availability

Analysis codes were made publicly available in this [GitHub Repository](https://github.com/gemini-duke/PRACT-3Months):

##

## Ethics and Privacy Considerations

In alignment with the guidelines set forth by the NIMH Data Archive, the data from this study will be made publicly available two years following the completion of data collection. This period allows for comprehensive reviews, verifications, and any necessary data anonymization to ensure it aligns with ethical and privacy norms before public release.

It is important to highlight that all patients involved in this study have been adequately informed about this data-sharing timeline and have given their informed consent accordingly. For those who opted not to have their data publicly shared, their preferences have been honored; thus, their data will not be included in the dataset that will become publicly available. This measure is in place to maintain the highest ethical standards while respecting both regulatory recommendations and the privacy concerns of our study participants.

All procedures were reviewed and approved by the Duke University Health System institutional review board (IRB), the National Institute for Medical Research National Health Research Ethics Committee (Tanzania), and the Kilimanjaro Christian Medical College Research Ethics and Review Committee.

## Descriptive Analysis

Patient demographics were examined across all study arms to understand the composition of each group. Data for these demographic variables was reported using a range of statistical measures: means, standard deviations, medians, interquartile ranges, and frequencies. Additionally, outcome data was organized and presented as predicted means, complete with confidence intervals for each timepoint under study.

## Inferential Analysis

The primary hypothesis concerning the efficacy of the intervention was tested using a Longitudinal Constrained Approach (LCA). To accommodate the count nature of the primary outcome-alcohol consumption data-and its overdispersion, as well as excess zero counts due to non-binge-drinking participants, the model was fitted using a zero-inflated negative binomial method with a log-link function.

Further, changes in the number of drinking days and the amount of alcohol consumed were analyzed through negative binomial models. Linear models were employed to evaluate changes in the Alcohol Use Disorders Identification Test (AUDIT) scores, the Drinker Inventory of Consequences (DrInC) scores and the Patient Health Questionnaire (PHQ-9) scores. For significant interaction terms, contrasts of marginal means were conducted, and the Wald chi-square test statistic was interpreted. Lastly, the effect size was reported in terms of differences in predicted means.

## Variables

### female

Variable Name: female

Description: Patient sex

Variable Type: String

Value Range: 0;1

Coding: 0 = Male;1 = Female

Measurement Unit: NA

Timepoints: Baseline, 3 Months

Data Processing Details: NA

NDA Element Name: sex

NDA Data Dictionary: Multiple

Necessary data transformations to share data with NDA: The value "Male" was recoded to "M" and the value "Female" was recoded to "F".

### age

Variable Name: age

Description: Age

Variable Type: Integer

Value Range: 18::120

Coding: NA

Measurement Unit: NA

Timepoints: Baseline, 3 Months

Data Processing Details: NA

NDA Element Name: interview_age

NDA Data Dictionary: Multiple

Necessary data transformations to share data with NDA: age in years is converted in age in months for sharing with the NDA

### tribe

Variable Name: tribe

Description: 5. What tribe do you affiliate with?

Variable Type: Integer

Value Range: 0;1;2;3;4;5;6;7;8;9;10;99

Coding: 0 = Chagga;1 = Pare;2 = Sambaa;3 = Maasai;4 = Iraq;5 = Sukuma;6 = Mmeru;7 = Nyaturu;8 = Muha;9 = Other African;10 = Non-African;99 = Refused/Don't know

Measurement Unit: NA

Timepoints: Baseline, 3 Months

Data Processing Details: NA

NDA Element Name: data structure not yet defined

NDA Data Dictionary: data structure not yet defined

Necessary data transformations to share data with NDA: NA

### tribe_recoded

Variable Name: tribe_recoded

Description: Recoding of the tribe variable.

Variable Type:

Value Range:

Coding:

Measurement Unit: NA

Timepoints: Baseline, 3 Months

Data Processing Details: all tribes other than "Chagga" and "Pare" under the generic label "Other" for the tribe_recoded variable.

NDA Element Name: data structure not yet defined

NDA Data Dictionary: data structure not yet defined

Necessary data transformations to share data with NDA: NA

### bacpositive

Variable Name: bacpositive

Description: Was blood alcohol levels positive (>0.00)?

Variable Type: Integer

Value Range: 0;1

Coding: 0 = No; 1 = Yes

Measurement Unit: NA

Timepoints: Baseline, 3 Months

Data Processing Details: NA

NDA Element Name: data structure not yet defined

NDA Data Dictionary: data structure not yet defined

Necessary data transformations to share data with NDA: NA

### edu_years

Variable Name: edu_years

Description: Years of education

Variable Type: Float

Value Range: 0::25

Coding: NA

Measurement Unit: NA

Timepoints: Baseline, 3 Months

Data Processing Details: NA

NDA Element Name: data structure not yet defined

NDA Data Dictionary: data structure not yet defined

Necessary data transformations to share data with NDA: NA

### employ

Variable Name: employ

Description: What is your current employment?

Variable Type: Integer

Value Range: 1;2;3;4;5;6;7;8;9;89;99

Coding: 1 = Professional; 2 = Clerical; 3 = Sales; 4 = Household; 5 = Agricultural - self-employed; 6 = Agricultural - employee; 7 = Services; 8 = Skilled manual; 9 = Unskilled manual; 89 = Other; 99 = Refused/Don't know.

Measurement Unit: NA

Timepoints: Baseline, 3 Months

Data Processing Details: Entries that were previously labeled "No I am a student" and had missing values for the employment category were re-labeled as "Student". Both the "Clerical" and "Skilled manual" categories were combined into a new category called "Skilled-employment". Meanwhile, the categories "Unskilled manual", "Household", "Services", and "Sales" were grouped together under the label "Unskilled-employment". The "Agricultural - self-employed" category was renamed to "Farmer (self-employed)", and "Agricultural - employee" was changed to "Farmer (employee)". The "Other" category remained unchanged, and "Refused/Don't know" was re-labeled as "Unknown".

NDA Element Name: data structure not yet defined

NDA Data Dictionary: data structure not yet defined

Necessary data transformations to share data with NDA: NA

### income_house

Variable Name: income_house

Description: total monthly income in your household

Variable Type: Integer

Value Range: 0::3e+07

Coding: NA

Measurement Unit: Tanzanian Shilling (TZS)

Timepoints: Baseline, 3 Months

Data Processing Details: NA

NDA Element Name: data structure not yet defined

NDA Data Dictionary: data structure not yet defined

Necessary data transformations to share data with NDA: NA

### income_self

Variable Name: income_self

Description: What is your income (per month)?

Variable Type: Integer

Value Range: 0::8e+06

Coding: NA

Measurement Unit: Tanzanian Shilling (TZS)

Timepoints: Baseline, 3 Months

Data Processing Details: NA

NDA Element Name: data structure not yet defined

NDA Data Dictionary: data structure not yet defined

Necessary data transformations to share data with NDA: NA

### drinkb4inj

Variable Name: drinkb4inj

Description: Did the patient report drinking alcohol in the 6 hours prior to injury?

Variable Type: Integer

Value Range: 0;1

Coding: 0 = No; 1 = Yes

Measurement Unit: NA

Timepoints: Baseline, 3 Months

Data Processing Details: NA

NDA Element Name: data structure not yet defined

NDA Data Dictionary: data structure not yet defined

Necessary data transformations to share data with NDA: NA

### drinc1

Variable Name: drinc1

Description: I have had a hangover or felt bad after drinking.

Variable Type: Integer

Value Range: 0;1;2;3

Coding: 0 = Never;1 = Once or a few times;2 = Once or twice a week;3 = Daily or almost daily

Measurement Unit: NA

Timepoints: Baseline, 3 Months

Data Processing Details:the DrInC variables were summed to calculate the DrInC score (Miller WR, Tonigan JS, Longabaugh R, Bethesda, MD: National Institute on Alcohol Abuse and Alcoholism, 1995). (Miller WR, Tonigan JS, Longabaugh R, Bethesda, MD: National Institute on Alcohol Abuse and Alcoholism, 1995).

NDA Element Name: drinc_01a

NDA Data Dictionary: The Drinker Inventory of Consequences (drinc01)

Necessary data transformations to share data with NDA: "Never" was coded as 0, "Once or a few times" was coded as 1, "Once or twice a week" was coded as 2, and "Daily or almost daily" was coded as 3.

### drinc2

Variable Name: drinc2

Description: I have felt bad about myself because of my drinking.

Variable Type: Integer

Value Range: 0;1;2;3

Coding: 0 = Never;1 = Once or a few times;2 = Once or twice a week;3 = Daily or almost daily

Measurement Unit: NA

Timepoints: Baseline, 3 Months

Data Processing Details:the DrInC variables were summed to calculate the DrInC score (Miller WR, Tonigan JS, Longabaugh R, Bethesda, MD: National Institute on Alcohol Abuse and Alcoholism, 1995).

NDA Element Name: drinc_02a

NDA Data Dictionary: The Drinker Inventory of Consequences (drinc01)

Necessary data transformations to share data with NDA: "Never" was coded as 0, "Once or a few times" was coded as 1, "Once or twice a week" was coded as 2, and "Daily or almost daily" was coded as 3.

### drinc3

Variable Name: drinc3

Description: I have missed days of work or school because of my drinking.

Variable Type: Integer

Value Range: 0;1;2;3

Coding: 0 = Never;1 = Once or a few times;2 = Once or twice a week;3 = Daily or almost daily

Measurement Unit: NA

Timepoints: Baseline, 3 Months

Data Processing Details:the DrInC variables were summed to calculate the DrInC score (Miller WR, Tonigan JS, Longabaugh R, Bethesda, MD: National Institute on Alcohol Abuse and Alcoholism, 1995).

NDA Element Name: drinc_03a

NDA Data Dictionary: The Drinker Inventory of Consequences (drinc01)

Necessary data transformations to share data with NDA: "Never" was coded as 0, "Once or a few times" was coded as 1, "Once or twice a week" was coded as 2, and "Daily or almost daily" was coded as 3.

### drinc4

Variable Name: drinc4

Description: My family or friends have worried or complained about my drinking.

Variable Type: Integer

Value Range: 0;1;2;3

Coding: 0 = Never;1 = Once or a few times;2 = Once or twice a week;3 = Daily or almost daily

Measurement Unit: NA

Timepoints: Baseline, 3 Months

Data Processing Details:the DrInC variables were summed to calculate the DrInC score (Miller WR, Tonigan JS, Longabaugh R, Bethesda, MD: National Institute on Alcohol Abuse and Alcoholism, 1995).

NDA Element Name: drinc_04a

NDA Data Dictionary: The Drinker Inventory of Consequences (drinc01)

Necessary data transformations to share data with NDA: "Never" was coded as 0, "Once or a few times" was coded as 1, "Once or twice a week" was coded as 2, and "Daily or almost daily" was coded as 3.

### drinc5

Variable Name: drinc5

Description: I have enjoyed the taste of beer, wine, or liquor.

Variable Type: Integer

Value Range: 0;1;2;3

Coding: 0 = Never;1 = Once or a few times;2 = Once or twice a week;3 = Daily or almost daily

Measurement Unit: NA

Timepoints: Baseline, 3 Months

Data Processing Details:the DrInC variables were summed to calculate the DrInC score (Miller WR, Tonigan JS, Longabaugh R, Bethesda, MD: National Institute on Alcohol Abuse and Alcoholism, 1995).

NDA Element Name: drinc_05a

NDA Data Dictionary: The Drinker Inventory of Consequences (drinc01)

Necessary data transformations to share data with NDA: "Never" was coded as 0, "Once or a few times" was coded as 1, "Once or twice a week" was coded as 2, and "Daily or almost daily" was coded as 3.

### drinc6

Variable Name: drinc6

Description:The quality of my work has suffered because of my drinking.

Variable Type: Integer

Value Range: 0;1;2;3

Coding: 0 = Never;1 = Once or a few times;2 = Once or twice a week;3 = Daily or almost daily

Measurement Unit: NA

Timepoints: Baseline, 3 Months

Data Processing Details:the DrInC variables were summed to calculate the DrInC score

NDA Element Name: drinc_06a

NDA Data Dictionary: The Drinker Inventory of Consequences (drinc01)

Necessary data transformations to share data with NDA: "Never" was coded as 0, "Once or a few times" was coded as 1, "Once or twice a week" was coded as 2, and "Daily or almost daily" was coded as 3.

### drinc7

Variable Name: drinc7

Description: My ability to be a good parent has been harmed by my drinking.

Variable Type: Integer

Value Range: 0;1;2;3

Coding: 0 = Never;1 = Once or a few times;2 = Once or twice a week;3 = Daily or almost daily

Measurement Unit: NA

Timepoints: Baseline, 3 Months

Data Processing Details:the DrInC variables were summed to calculate the DrInC score (Miller WR, Tonigan JS, Longabaugh R, Bethesda, MD: National Institute on Alcohol Abuse and Alcoholism, 1995).

NDA Element Name: drinc_07a

NDA Data Dictionary: The Drinker Inventory of Consequences (drinc01)

Necessary data transformations to share data with NDA: "Never" was coded as 0, "Once or a few times" was coded as 1, "Once or twice a week" was coded as 2, and "Daily or almost daily" was coded as 3.

### drinc8

Variable Name: drinc8

Description: After drinking, I have had trouble with sleeping, staying asleep, or nightmares.

Variable Type: Integer

Value Range: 0;1;2;3

Coding: 0 = Never;1 = Once or a few times;2 = Once or twice a week;3 = Daily or almost daily

Measurement Unit: NA

Timepoints: Baseline, 3 Months

Data Processing Details:the DrInC variables were summed to calculate the DrInC score (Miller WR, Tonigan JS, Longabaugh R, Bethesda, MD: National Institute on Alcohol Abuse and Alcoholism, 1995).

NDA Element Name: drinc_08a

NDA Data Dictionary: The Drinker Inventory of Consequences (drinc01)

Necessary data transformations to share data with NDA: "Never" was coded as 0, "Once or a few times" was coded as 1, "Once or twice a week" was coded as 2, and "Daily or almost daily" was coded as 3.

### drinc9

Variable Name: drinc9

Description: I have driven a motor vehicle after having three or more drinks.

Variable Type: Integer

Value Range: 0;1;2;3

Coding: 0 = Never;1 = Once or a few times;2 = Once or twice a week;3 = Daily or almost daily

Measurement Unit: NA

Timepoints: Baseline, 3 Months

Data Processing Details:the DrInC variables were summed to calculate the DrInC score (Miller WR, Tonigan JS, Longabaugh R, Bethesda, MD: National Institute on Alcohol Abuse and Alcoholism, 1995).

NDA Element Name: drinc_09a

NDA Data Dictionary: The Drinker Inventory of Consequences (drinc01)

Necessary data transformations to share data with NDA: "Never" was coded as 0, "Once or a few times" was coded as 1, "Once or twice a week" was coded as 2, and "Daily or almost daily" was coded as 3.

### drinc10

Variable Name: drinc10

Description: My drinking has caused me to use other drugs more.

Variable Type: Integer

Value Range: 0;1;2;3

Coding: 0 = Never;1 = Once or a few times;2 = Once or twice a week;3 = Daily or almost daily

Measurement Unit: NA

Timepoints: Baseline, 3 Months

Data Processing Details:the DrInC variables were summed to calculate the DrInC score (Miller WR, Tonigan JS, Longabaugh R, Bethesda, MD: National Institute on Alcohol Abuse and Alcoholism, 1995).

NDA Element Name: drinc_10a

NDA Data Dictionary: The Drinker Inventory of Consequences (drinc01)

Necessary data transformations to share data with NDA: "Never" was coded as 0, "Once or a few times" was coded as 1, "Once or twice a week" was coded as 2, and "Daily or almost daily" was coded as 3.

### drinc11

Variable Name: drinc11

Description: I have been sick and vomited after drinking.

Variable Type: Integer

Value Range: 0;1;2;3

Coding: 0 = Never;1 = Once or a few times;2 = Once or twice a week;3 = Daily or almost daily

Measurement Unit: NA

Timepoints: Baseline, 3 Months

Data Processing Details:the DrInC variables were summed to calculate the DrInC score (Miller WR, Tonigan JS, Longabaugh R, Bethesda, MD: National Institute on Alcohol Abuse and Alcoholism, 1995).

NDA Element Name: drinc_11a

NDA Data Dictionary: The Drinker Inventory of Consequences (drinc01)

Necessary data transformations to share data with NDA: "Never" was coded as 0, "Once or a few times" was coded as 1, "Once or twice a week" was coded as 2, and "Daily or almost daily" was coded as 3.

### drinc12

Variable Name: drinc12

Description: I have been unhappy because of my drinking.

Variable Type: Integer

Value Range: 0;1;2;3

Coding: 0 = Never;1 = Once or a few times;2 = Once or twice a week;3 = Daily or almost daily

Measurement Unit: NA

Timepoints: Baseline, 3 Months

Data Processing Details:the DrInC variables were summed to calculate the DrInC score (Miller WR, Tonigan JS, Longabaugh R, Bethesda, MD: National Institute on Alcohol Abuse and Alcoholism, 1995).

NDA Element Name: drinc_12a

NDA Data Dictionary: The Drinker Inventory of Consequences (drinc01)

Necessary data transformations to share data with NDA: "Never" was coded as 0, "Once or a few times" was coded as 1, "Once or twice a week" was coded as 2, and "Daily or almost daily" was coded as 3.

### drinc13

Variable Name: drinc13

Description: Because of my drinking, I have not eaten properly.

Variable Type: Integer

Value Range: 0;1;2;3

Coding: 0 = Never;1 = Once or a few times;2 = Once or twice a week;3 = Daily or almost daily

Measurement Unit: NA

Timepoints: Baseline, 3 Months

Data Processing Details:the DrInC variables were summed to calculate the DrInC score (Miller WR, Tonigan JS, Longabaugh R, Bethesda, MD: National Institute on Alcohol Abuse and Alcoholism, 1995).

NDA Element Name: drinc_13a

NDA Data Dictionary: The Drinker Inventory of Consequences (drinc01)

Necessary data transformations to share data with NDA: "Never" was coded as 0, "Once or a few times" was coded as 1, "Once or twice a week" was coded as 2, and "Daily or almost daily" was coded as 3.

### drinc14

Variable Name: drinc14

Description: I have failed to do what is expected of me because of my drinking.

Variable Type: Integer

Value Range: 0;1;2;3

Coding: 0 = Never;1 = Once or a few times;2 = Once or twice a week;3 = Daily or almost daily

Measurement Unit: NA

Timepoints: Baseline, 3 Months

Data Processing Details:the DrInC variables were summed to calculate the DrInC score (Miller WR, Tonigan JS, Longabaugh R, Bethesda, MD: National Institute on Alcohol Abuse and Alcoholism, 1995).

NDA Element Name: drinc_14a

NDA Data Dictionary: The Drinker Inventory of Consequences (drinc01)

Necessary data transformations to share data with NDA: "Never" was coded as 0, "Once or a few times" was coded as 1, "Once or twice a week" was coded as 2, and "Daily or almost daily" was coded as 3.

### drinc15

Variable Name: drinc15

Description: Drinking has helped me to relax

Variable Type: Integer

Value Range: 0;1;2;3

Coding: 0 = Never;1 = Once or a few times;2 = Once or twice a week;3 = Daily or almost daily

Measurement Unit: NA

Timepoints: Baseline, 3 Months

Data Processing Details:the DrInC variables were summed to calculate the DrInC score (Miller WR, Tonigan JS, Longabaugh R, Bethesda, MD: National Institute on Alcohol Abuse and Alcoholism, 1995).

NDA Element Name: drinc_15a

NDA Data Dictionary: The Drinker Inventory of Consequences (drinc01)

Necessary data transformations to share data with NDA: "Never" was coded as 0, "Once or a few times" was coded as 1, "Once or twice a week" was coded as 2, and "Daily or almost daily" was coded as 3.

### drinc16

Variable Name: drinc16

Description: I have felt guilty or ashamed because of my drinking.

Variable Type: Integer

Value Range: 0;1;2;3

Coding: 0 = Never;1 = Once or a few times;2 = Once or twice a week;3 = Daily or almost daily

Measurement Unit: NA

Timepoints: Baseline, 3 Months

Data Processing Details:the DrInC variables were summed to calculate the DrInC score (Miller WR, Tonigan JS, Longabaugh R, Bethesda, MD: National Institute on Alcohol Abuse and Alcoholism, 1995).

NDA Element Name: drinc_16a

NDA Data Dictionary: The Drinker Inventory of Consequences (drinc01)

Necessary data transformations to share data with NDA: "Never" was coded as 0, "Once or a few times" was coded as 1, "Once or twice a week" was coded as 2, and "Daily or almost daily" was coded as 3.

### drinc17

Variable Name: drinc17

Description: While drinking, I have said or done embarrassing things.

Variable Type: Integer

Value Range: 0;1;2;3

Coding: 0 = Never;1 = Once or a few times;2 = Once or twice a week;3 = Daily or almost daily

Measurement Unit: NA

Timepoints: Baseline, 3 Months

Data Processing Details:the DrInC variables were summed to calculate the DrInC score (Miller WR, Tonigan JS, Longabaugh R, Bethesda, MD: National Institute on Alcohol Abuse and Alcoholism, 1995).

NDA Element Name: drinc_17a

NDA Data Dictionary: The Drinker Inventory of Consequences (drinc01)

Necessary data transformations to share data with NDA: "Never" was coded as 0, "Once or a few times" was coded as 1, "Once or twice a week" was coded as 2, and "Daily or almost daily" was coded as 3.

### drinc18

Variable Name: drinc18

Description: When drinking, my personality has changed for the worse.

Variable Type: Integer

Value Range: 0;1;2;3

Coding: 0 = Never;1 = Once or a few times;2 = Once or twice a week;3 = Daily or almost daily

Measurement Unit: NA

Timepoints: Baseline, 3 Months

Data Processing Details:the DrInC variables were summed to calculate the DrInC score (Miller WR, Tonigan JS, Longabaugh R, Bethesda, MD: National Institute on Alcohol Abuse and Alcoholism, 1995).

NDA Element Name: drinc_18a

NDA Data Dictionary: The Drinker Inventory of Consequences (drinc01)

Necessary data transformations to share data with NDA: "Never" was coded as 0, "Once or a few times" was coded as 1, "Once or twice a week" was coded as 2, and "Daily or almost daily" was coded as 3.

### drinc19

Variable Name: drinc19

Description: I have taken foolish risks when I have been drinking.

Variable Type: Integer

Value Range: 0;1;2;3

Coding: 0 = Never;1 = Once or a few times;2 = Once or twice a week;3 = Daily or almost daily

Measurement Unit: NA

Timepoints: Baseline, 3 Months

Data Processing Details:the DrInC variables were summed to calculate the DrInC score (Miller WR, Tonigan JS, Longabaugh R, Bethesda, MD: National Institute on Alcohol Abuse and Alcoholism, 1995).

NDA Element Name: drinc_19a

NDA Data Dictionary: The Drinker Inventory of Consequences (drinc01)

Necessary data transformations to share data with NDA: "Never" was coded as 0, "Once or a few times" was coded as 1, "Once or twice a week" was coded as 2, and "Daily or almost daily" was coded as 3.

### drinc20

Variable Name: drinc20

Description: I have gotten into trouble because of drinking.

Variable Type: Integer

Value Range: 0;1;2;3

Coding: 0 = Never;1 = Once or a few times;2 = Once or twice a week;3 = Daily or almost daily

Measurement Unit: NA

Timepoints: Baseline, 3 Months

Data Processing Details:the DrInC variables were summed to calculate the DrInC score (Miller WR, Tonigan JS, Longabaugh R, Bethesda, MD: National Institute on Alcohol Abuse and Alcoholism, 1995).

NDA Element Name: drinc_20a

NDA Data Dictionary: The Drinker Inventory of Consequences (drinc01)

Necessary data transformations to share data with NDA: "Never" was coded as 0, "Once or a few times" was coded as 1, "Once or twice a week" was coded as 2, and "Daily or almost daily" was coded as 3.

### drinc21

Variable Name: drinc21

Description: While drinking or using drugs, I have said harsh or cruel things to someone.

Variable Type: Integer

Value Range: 0;1;2;3

Coding: 0 = Never;1 = Once or a few times;2 = Once or twice a week;3 = Daily or almost daily

Measurement Unit: NA

Timepoints: Baseline, 3 Months

Data Processing Details:the DrInC variables were summed to calculate the DrInC score (Miller WR, Tonigan JS, Longabaugh R, Bethesda, MD: National Institute on Alcohol Abuse and Alcoholism, 1995).

NDA Element Name: drinc_21a

NDA Data Dictionary: The Drinker Inventory of Consequences (drinc01)

Necessary data transformations to share data with NDA: "Never" was coded as 0, "Once or a few times" was coded as 1, "Once or twice a week" was coded as 2, and "Daily or almost daily" was coded as 3.

### drinc22

Variable Name: drinc22

Description: When drinking, I have done impulsive things that I regretted later

Variable Type: Integer

Value Range: 0;1;2;3

Coding: 0 = Never;1 = Once or a few times;2 = Once or twice a week;3 = Daily or almost daily

Measurement Unit: NA

Timepoints: Baseline, 3 Months

Data Processing Details:the DrInC variables were summed to calculate the DrInC score (Miller WR, Tonigan JS, Longabaugh R, Bethesda, MD: National Institute on Alcohol Abuse and Alcoholism, 1995).

NDA Element Name: drinc_22a

NDA Data Dictionary: The Drinker Inventory of Consequences (drinc01)

Necessary data transformations to share data with NDA: "Never" was coded as 0, "Once or a few times" was coded as 1, "Once or twice a week" was coded as 2, and "Daily or almost daily" was coded as 3.

### drinc23

Variable Name: drinc23

Description: I have gotten into a physical fight while drinking

Variable Type: Integer

Value Range: 0;1;2;3

Coding: 0 = Never;1 = Once or a few times;2 = Once or twice a week;3 = Daily or almost daily

Measurement Unit: NA

Timepoints: Baseline, 3 Months

Data Processing Details:the DrInC variables were summed to calculate the DrInC score (Miller WR, Tonigan JS, Longabaugh R, Bethesda, MD: National Institute on Alcohol Abuse and Alcoholism, 1995).

NDA Element Name: drinc_23a

NDA Data Dictionary: The Drinker Inventory of Consequences (drinc01)

Necessary data transformations to share data with NDA: "Never" was coded as 0, "Once or a few times" was coded as 1, "Once or twice a week" was coded as 2, and "Daily or almost daily" was coded as 3.

### drinc24

Variable Name: drinc24

Description: My physical health has been harmed by my drinking.

Variable Type: Integer

Value Range: 0;1;2;3

Coding: 0 = Never;1 = Once or a few times;2 = Once or twice a week;3 = Daily or almost daily

Measurement Unit: NA

Timepoints: Baseline, 3 Months

Data Processing Details:the DrInC variables were summed to calculate the DrInC score (Miller WR, Tonigan JS, Longabaugh R, Bethesda, MD: National Institute on Alcohol Abuse and Alcoholism, 1995).

NDA Element Name: drinc_24a

NDA Data Dictionary: The Drinker Inventory of Consequences (drinc01)

Necessary data transformations to share data with NDA: "Never" was coded as 0, "Once or a few times" was coded as 1, "Once or twice a week" was coded as 2, and "Daily or almost daily" was coded as 3.

### drinc25

Variable Name: drinc25

Description: Drinking has helped me to have a more positive outlook on life.

Variable Type: Integer

Value Range: 0;1;2;3

Coding: 0 = Never;1 = Once or a few times;2 = Once or twice a week;3 = Daily or almost daily

Measurement Unit: NA

Timepoints: Baseline, 3 Months

Data Processing Details:the DrInC variables were summed to calculate the DrInC score (Miller WR, Tonigan JS, Longabaugh R, Bethesda, MD: National Institute on Alcohol Abuse and Alcoholism, 1995).

NDA Element Name: drinc_25a

NDA Data Dictionary: The Drinker Inventory of Consequences (drinc01)

Necessary data transformations to share data with NDA: "Never" was coded as 0, "Once or a few times" was coded as 1, "Once or twice a week" was coded as 2, and "Daily or almost daily" was coded as 3.

### drinc26

Variable Name: drinc26

Description: I have had money problems because of my drinking.

Variable Type: Integer

Value Range: 0;1;2;3

Coding: 0 = Never;1 = Once or a few times;2 = Once or twice a week;3 = Daily or almost daily

Measurement Unit: NA

Timepoints: Baseline, 3 Months

Data Processing Details:the DrInC variables were summed to calculate the DrInC score (Miller WR, Tonigan JS, Longabaugh R, Bethesda, MD: National Institute on Alcohol Abuse and Alcoholism, 1995).

NDA Element Name: drinc_26a

NDA Data Dictionary: The Drinker Inventory of Consequences (drinc01)

Necessary data transformations to share data with NDA: "Never" was coded as 0, "Once or a few times" was coded as 1, "Once or twice a week" was coded as 2, and "Daily or almost daily" was coded as 3.

### drinc27

Variable Name: drinc27

Description: My marriage or love relationship has been harmed by my drinking.

Variable Type: Integer

Value Range: 0;1;2;3

Coding: 0 = Never;1 = Once or a few times;2 = Once or twice a week;3 = Daily or almost daily

Measurement Unit: NA

Timepoints: Baseline, 3 Months

Data Processing Details:the DrInC variables were summed to calculate the DrInC score (Miller WR, Tonigan JS, Longabaugh R, Bethesda, MD: National Institute on Alcohol Abuse and Alcoholism, 1995).

NDA Element Name: drinc_27a

NDA Data Dictionary: The Drinker Inventory of Consequences (drinc01)

Necessary data transformations to share data with NDA: "Never" was coded as 0, "Once or a few times" was coded as 1, "Once or twice a week" was coded as 2, and "Daily or almost daily" was coded as 3.

### drinc28

Variable Name: drinc28

Description: I have smoked tobacco more when I am drinking.

Variable Type: Integer

Value Range: 0;1;2;3

Coding: 0 = Never;1 = Once or a few times;2 = Once or twice a week;3 = Daily or almost daily

Measurement Unit: NA

Timepoints: Baseline, 3 Months

Data Processing Details:the DrInC variables were summed to calculate the DrInC score (Miller WR, Tonigan JS, Longabaugh R, Bethesda, MD: National Institute on Alcohol Abuse and Alcoholism, 1995).

NDA Element Name: drinc_28a

NDA Data Dictionary: The Drinker Inventory of Consequences (drinc01)

Necessary data transformations to share data with NDA: "Never" was coded as 0, "Once or a few times" was coded as 1, "Once or twice a week" was coded as 2, and "Daily or almost daily" was coded as 3.

### drinc29

Variable Name: drinc29

Description: My physical appearance has been harmed by my drinking.

Variable Type: Integer

Value Range: 0;1;2;3

Coding: 0 = Never;1 = Once or a few times;2 = Once or twice a week;3 = Daily or almost daily

Measurement Unit: NA

Timepoints: Baseline, 3 Months

Data Processing Details:the DrInC variables were summed to calculate the DrInC score (Miller WR, Tonigan JS, Longabaugh R, Bethesda, MD: National Institute on Alcohol Abuse and Alcoholism, 1995).

NDA Element Name: drinc_29a

NDA Data Dictionary: The Drinker Inventory of Consequences (drinc01)

Necessary data transformations to share data with NDA: "Never" was coded as 0, "Once or a few times" was coded as 1, "Once or twice a week" was coded as 2, and "Daily or almost daily" was coded as 3.

### drinc30

Variable Name: drinc30

Description: My family has been hurt by my drinking.

Variable Type: Integer

Value Range: 0;1;2;3

Coding: 0 = Never;1 = Once or a few times;2 = Once or twice a week;3 = Daily or almost daily

Measurement Unit: NA

Timepoints: Baseline, 3 Months

Data Processing Details:the DrInC variables were summed to calculate the DrInC score (Miller WR, Tonigan JS, Longabaugh R, Bethesda, MD: National Institute on Alcohol Abuse and Alcoholism, 1995).

NDA Element Name: drinc_30a

NDA Data Dictionary: The Drinker Inventory of Consequences (drinc01)

Necessary data transformations to share data with NDA: "Never" was coded as 0, "Once or a few times" was coded as 1, "Once or twice a week" was coded as 2, and "Daily or almost daily" was coded as 3.

### drinc31

Variable Name: drinc31

Description: A friendship or close relationship has been damaged by my drinking.

Variable Type: Integer

Value Range: 0;1;2;3

Coding: 0 = Never;1 = Once or a few times;2 = Once or twice a week;3 = Daily or almost daily

Measurement Unit: NA

Timepoints: Baseline, 3 Months

Data Processing Details:the DrInC variables were summed to calculate the DrInC score (Miller WR, Tonigan JS, Longabaugh R, Bethesda, MD: National Institute on Alcohol Abuse and Alcoholism, 1995).

NDA Element Name: drinc_31a

NDA Data Dictionary: The Drinker Inventory of Consequences (drinc01)

Necessary data transformations to share data with NDA: "Never" was coded as 0, "Once or a few times" was coded as 1, "Once or twice a week" was coded as 2, and "Daily or almost daily" was coded as 3.

### drinc32

Variable Name: drinc32

Description: I have been overweight because of my drinking

Variable Type: Integer

Value Range: 0;1;2;3

Coding: 0 = Never;1 = Once or a few times;2 = Once or twice a week;3 = Daily or almost daily

Measurement Unit: NA

Timepoints: Baseline, 3 Months

Data Processing Details:the DrInC variables were summed to calculate the DrInC score (Miller WR, Tonigan JS, Longabaugh R, Bethesda, MD: National Institute on Alcohol Abuse and Alcoholism, 1995).

NDA Element Name: drinc_32a

NDA Data Dictionary: The Drinker Inventory of Consequences (drinc01)

Necessary data transformations to share data with NDA: "Never" was coded as 0, "Once or a few times" was coded as 1, "Once or twice a week" was coded as 2, and "Daily or almost daily" was coded as 3.

### drinc33

Variable Name: drinc33

Description: My sex life has suffered because of my drinking.

Variable Type: Integer

Value Range: 0;1;2;3

Coding: 0 = Never;1 = Once or a few times;2 = Once or twice a week;3 = Daily or almost daily

Measurement Unit: NA

Timepoints: Baseline, 3 Months

Data Processing Details:the DrInC variables were summed to calculate the DrInC score (Miller WR, Tonigan JS, Longabaugh R, Bethesda, MD: National Institute on Alcohol Abuse and Alcoholism, 1995).

NDA Element Name: drinc_33a

NDA Data Dictionary: The Drinker Inventory of Consequences (drinc01)

Necessary data transformations to share data with NDA: "Never" was coded as 0, "Once or a few times" was coded as 1, "Once or twice a week" was coded as 2, and "Daily or almost daily" was coded as 3.

### drinc34

Variable Name: drinc34

Description: I have lost interest in activities and hobbies because of my drinking.

Variable Type: Integer

Value Range: 0;1;2;3

Coding: 0 = Never;1 = Once or a few times;2 = Once or twice a week;3 = Daily or almost daily

Measurement Unit: NA

Timepoints: Baseline, 3 Months

Data Processing Details:the DrInC variables were summed to calculate the DrInC score (Miller WR, Tonigan JS, Longabaugh R, Bethesda, MD: National Institute on Alcohol Abuse and Alcoholism, 1995).

NDA Element Name: drinc_34a

NDA Data Dictionary: The Drinker Inventory of Consequences (drinc01)

Necessary data transformations to share data with NDA: "Never" was coded as 0, "Once or a few times" was coded as 1, "Once or twice a week" was coded as 2, and "Daily or almost daily" was coded as 3.

### drinc35

Variable Name: drinc35

Description: When drinking, my social life has been more enjoyable

Variable Type: Integer

Value Range: 0;1;2;3

Coding: 0 = Never;1 = Once or a few times;2 = Once or twice a week;3 = Daily or almost daily

Measurement Unit: NA

Timepoints: Baseline, 3 Months

Data Processing Details:the DrInC variables were summed to calculate the DrInC score (Miller WR, Tonigan JS, Longabaugh R, Bethesda, MD: National Institute on Alcohol Abuse and Alcoholism, 1995).

NDA Element Name: drinc_35a

NDA Data Dictionary: The Drinker Inventory of Consequences (drinc01)

Necessary data transformations to share data with NDA: "Never" was coded as 0, "Once or a few times" was coded as 1, "Once or twice a week" was coded as 2, and "Daily or almost daily" was coded as 3.

### drinc36

Variable Name: drinc36

Description: My spiritual or moral life has been harmed by my drinking.

Variable Type: Integer

Value Range: 0;1;2;3

Coding: 0 = Never;1 = Once or a few times;2 = Once or twice a week;3 = Daily or almost daily

Measurement Unit: NA

Timepoints: Baseline, 3 Months

Data Processing Details:the DrInC variables were summed to calculate the DrInC score (Miller WR, Tonigan JS, Longabaugh R, Bethesda, MD: National Institute on Alcohol Abuse and Alcoholism, 1995).

NDA Element Name: drinc_36a

NDA Data Dictionary: The Drinker Inventory of Consequences (drinc01)

Necessary data transformations to share data with NDA: "Never" was coded as 0, "Once or a few times" was coded as 1, "Once or twice a week" was coded as 2, and "Daily or almost daily" was coded as 3.

### drinc37

Variable Name: drinc37

Description: Because of my drinking, I have not had the kind of life that I want

Variable Type: Integer

Value Range: 0;1;2;3

Coding: 0 = Never;1 = Once or a few times;2 = Once or twice a week;3 = Daily or almost daily

Measurement Unit: NA

Timepoints: Baseline, 3 Months

Data Processing Details:the DrInC variables were summed to calculate the DrInC score (Miller WR, Tonigan JS, Longabaugh R, Bethesda, MD: National Institute on Alcohol Abuse and Alcoholism, 1995).

NDA Element Name: drinc_37a

NDA Data Dictionary: The Drinker Inventory of Consequences (drinc01)

Necessary data transformations to share data with NDA: "Never" was coded as 0, "Once or a few times" was coded as 1, "Once or twice a week" was coded as 2, and "Daily or almost daily" was coded as 3.

### drinc38

Variable Name: drinc38

Description: My drinking has gotten in the way of my growth as a person

Variable Type: Integer

Value Range: 0;1;2;3

Coding: 0 = Never;1 = Once or a few times;2 = Once or twice a week;3 = Daily or almost daily

Measurement Unit: NA

Timepoints: Baseline, 3 Months

Data Processing Details:the DrInC variables were summed to calculate the DrInC score (Miller WR, Tonigan JS, Longabaugh R, Bethesda, MD: National Institute on Alcohol Abuse and Alcoholism, 1995).

NDA Element Name: drinc_38a

NDA Data Dictionary: The Drinker Inventory of Consequences (drinc01)

Necessary data transformations to share data with NDA: "Never" was coded as 0, "Once or a few times" was coded as 1, "Once or twice a week" was coded as 2, and "Daily or almost daily" was coded as 3.

### drinc39

Variable Name: drinc39

Description: My drinking has damaged my social life, popularity, or reputation

Variable Type: Integer

Value Range: 0;1;2;3

Coding: 0 = Never;1 = Once or a few times;2 = Once or twice a week;3 = Daily or almost daily

Measurement Unit: NA

Timepoints: Baseline, 3 Months

Data Processing Details:the DrInC variables were summed to calculate the DrInC score (Miller WR, Tonigan JS, Longabaugh R, Bethesda, MD: National Institute on Alcohol Abuse and Alcoholism, 1995).

NDA Element Name: drinc_39a

NDA Data Dictionary: The Drinker Inventory of Consequences (drinc01)

Necessary data transformations to share data with NDA: "Never" was coded as 0, "Once or a few times" was coded as 1, "Once or twice a week" was coded as 2, and "Daily or almost daily" was coded as 3.

### drinc40

Variable Name: drinc40

Description: I have spent too much or lost a lot of money because of my drinking.

Variable Type: Integer

Value Range: 0;1;2;3

Coding: 0 = Never;1 = Once or a few times;2 = Once or twice a week;3 = Daily or almost daily

Measurement Unit: NA

Timepoints: Baseline, 3 Months

Data Processing Details:the DrInC variables were summed to calculate the DrInC score (Miller WR, Tonigan JS, Longabaugh R, Bethesda, MD: National Institute on Alcohol Abuse and Alcoholism, 1995).

NDA Element Name: drinc_40a

NDA Data Dictionary: The Drinker Inventory of Consequences (drinc01)

Necessary data transformations to share data with NDA: "Never" was coded as 0, "Once or a few times" was coded as 1, "Once or twice a week" was coded as 2, and "Daily or almost daily" was coded as 3.

### drinc41

Variable Name: drinc41

Description: I have been arrested for driving under the influence of alcohol.

Variable Type: Integer

Value Range: 0;1;2;3

Coding: 0 = Never;1 = Once or a few times;2 = Once or twice a week;3 = Daily or almost daily

Measurement Unit: NA

Timepoints: Baseline, 3 Months

Data Processing Details:the DrInC variables were summed to calculate the DrInC score (Miller WR, Tonigan JS, Longabaugh R, Bethesda, MD: National Institute on Alcohol Abuse and Alcoholism, 1995).

NDA Element Name: drinc_41a

NDA Data Dictionary: The Drinker Inventory of Consequences (drinc01)

Necessary data transformations to share data with NDA: "Never" was coded as 0, "Once or a few times" was coded as 1, "Once or twice a week" was coded as 2, and "Daily or almost daily" was coded as 3.

### drinc42

Variable Name: drinc42

Description: have had trouble with the law (other than driving while intoxicated) because of my drinking.

Variable Type: Integer

Value Range: 0;1;2;3

Coding: 0 = Never;1 = Once or a few times;2 = Once or twice a week;3 = Daily or almost daily

Measurement Unit: NA

Timepoints: Baseline, 3 Months

Data Processing Details:the DrInC variables were summed to calculate the DrInC score (Miller WR, Tonigan JS, Longabaugh R, Bethesda, MD: National Institute on Alcohol Abuse and Alcoholism, 1995).

NDA Element Name: drinc_42a

NDA Data Dictionary: The Drinker Inventory of Consequences (drinc01)

Necessary data transformations to share data with NDA: "Never" was coded as 0, "Once or a few times" was coded as 1, "Once or twice a week" was coded as 2, and "Daily or almost daily" was coded as 3.

### drinc43

Variable Name: drinc43

Description: I have lost a marriage or a close love relationship because of my drinking.

Variable Type: Integer

Value Range: 0;1;2;3

Coding: 0 = Never;1 = Once or a few times;2 = Once or twice a week;3 = Daily or almost daily

Measurement Unit: NA

Timepoints: Baseline, 3 Months

Data Processing Details:the DrInC variables were summed to calculate the DrInC score (Miller WR, Tonigan JS, Longabaugh R, Bethesda, MD: National Institute on Alcohol Abuse and Alcoholism, 1995).

NDA Element Name: drinc_43a

NDA Data Dictionary: The Drinker Inventory of Consequences (drinc01)

Necessary data transformations to share data with NDA: "Never" was coded as 0, "Once or a few times" was coded as 1, "Once or twice a week" was coded as 2, and "Daily or almost daily" was coded as 3.

### drinc44

Variable Name: drinc44

Description: I have been suspended/fired from or left a job or school because of my drinking.

Variable Type: Integer

Value Range: 0;1;2;3

Coding: 0 = Never;1 = Once or a few times;2 = Once or twice a week;3 = Daily or almost daily

Measurement Unit: NA

Timepoints: Baseline, 3 Months

Data Processing Details:the DrInC variables were summed to calculate the DrInC score (Miller WR, Tonigan JS, Longabaugh R, Bethesda, MD: National Institute on Alcohol Abuse and Alcoholism, 1995).

NDA Element Name: drinc_44a

NDA Data Dictionary: The Drinker Inventory of Consequences (drinc01)

Necessary data transformations to share data with NDA: "Never" was coded as 0, "Once or a few times" was coded as 1, "Once or twice a week" was coded as 2, and "Daily or almost daily" was coded as 3.

### drinc45

Variable Name: drinc45

Description: I drank alcohol normally, without any problems.

Variable Type: Integer

Value Range: 0;1;2;3

Coding: 0 = Never;1 = Once or a few times;2 = Once or twice a week;3 = Daily or almost daily

Measurement Unit: NA

Timepoints: Baseline, 3 Months

Data Processing Details:the DrInC variables were summed to calculate the DrInC score (Miller WR, Tonigan JS, Longabaugh R, Bethesda, MD: National Institute on Alcohol Abuse and Alcoholism, 1995).

NDA Element Name: drinc_45a

NDA Data Dictionary: The Drinker Inventory of Consequences (drinc01)

Necessary data transformations to share data with NDA: "Never" was coded as 0, "Once or a few times" was coded as 1, "Once or twice a week" was coded as 2, and "Daily or almost daily" was coded as 3.

### drinc46

Variable Name: drinc46

Description: I have lost a friend because of my drinking.

Variable Type: Integer

Value Range: 0;1;2;3

Coding: 0 = Never;1 = Once or a few times;2 = Once or twice a week;3 = Daily or almost daily

Measurement Unit: NA

Timepoints: Baseline, 3 Months

Data Processing Details:the DrInC variables were summed to calculate the DrInC score (Miller WR, Tonigan JS, Longabaugh R, Bethesda, MD: National Institute on Alcohol Abuse and Alcoholism, 1995).

NDA Element Name: drinc_46a

NDA Data Dictionary: The Drinker Inventory of Consequences (drinc01)

Necessary data transformations to share data with NDA: "Never" was coded as 0, "Once or a few times" was coded as 1, "Once or twice a week" was coded as 2, and "Daily or almost daily" was coded as 3.

### drinc47

Variable Name: drinc47

Description: I have had an accident while drinking or intoxicated.

Variable Type: Integer

Value Range: 0;1;2;3

Coding: 0 = Never;1 = Once or a few times;2 = Once or twice a week;3 = Daily or almost daily

Measurement Unit: NA

Timepoints: Baseline, 3 Months

Data Processing Details:the DrInC variables were summed to calculate the DrInC score (Miller WR, Tonigan JS, Longabaugh R, Bethesda, MD: National Institute on Alcohol Abuse and Alcoholism, 1995).

NDA Element Name: drinc_47a

NDA Data Dictionary: The Drinker Inventory of Consequences (drinc01)

Necessary data transformations to share data with NDA: "Never" was coded as 0, "Once or a few times" was coded as 1, "Once or twice a week" was coded as 2, and "Daily or almost daily" was coded as 3.

### drinc48

Variable Name: drinc48

Description: While drinking or intoxicated, I have been physically hurt, injured, or burned.

Variable Type: Integer

Value Range: 0;1;2;3

Coding: 0 = Never;1 = Once or a few times;2 = Once or twice a week;3 = Daily or almost daily

Measurement Unit: NA

Timepoints: Baseline, 3 Months

Data Processing Details:the DrInC variables were summed to calculate the DrInC score (Miller WR, Tonigan JS, Longabaugh R, Bethesda, MD: National Institute on Alcohol Abuse and Alcoholism, 1995).

NDA Element Name: drinc_48a

NDA Data Dictionary: The Drinker Inventory of Consequences (drinc01)

Necessary data transformations to share data with NDA: "Never" was coded as 0, "Once or a few times" was coded as 1, "Once or twice a week" was coded as 2, and "Daily or almost daily" was coded as 3.

### drinc49

Variable Name: drinc49

Description: While drinking or intoxicated, I have injured someone else.

Variable Type: Integer

Value Range: 0;1;2;3

Coding: 0 = Never;1 = Once or a few times;2 = Once or twice a week;3 = Daily or almost daily

Measurement Unit: NA

Timepoints: Baseline, 3 Months

Data Processing Details:the DrInC variables were summed to calculate the DrInC score (Miller WR, Tonigan JS, Longabaugh R, Bethesda, MD: National Institute on Alcohol Abuse and Alcoholism, 1995).

NDA Element Name: drinc_49a

NDA Data Dictionary: The Drinker Inventory of Consequences (drinc01)

Necessary data transformations to share data with NDA: "Never" was coded as 0, "Once or a few times" was coded as 1, "Once or twice a week" was coded as 2, and "Daily or almost daily" was coded as 3.

### drinc50

Variable Name: drinc50

Description: I have broken things while drinking or intoxicated.

Variable Type: Integer

Value Range: 0;1;2;3

Coding: 0 = Never;1 = Once or a few times;2 = Once or twice a week;3 = Daily or almost daily

Measurement Unit: NA

Timepoints: Baseline, 3 Months

Data Processing Details:the DrInC variables were summed to calculate the DrInC score (Miller WR, Tonigan JS, Longabaugh R, Bethesda, MD: National Institute on Alcohol Abuse and Alcoholism, 1995).

NDA Element Name: drinc_50a

NDA Data Dictionary: The Drinker Inventory of Consequences (drinc01)

Necessary data transformations to share data with NDA: "Never" was coded as 0, "Once or a few times" was coded as 1, "Once or twice a week" was coded as 2, and "Daily or almost daily" was coded as 3.

### drinc_sum

Variable Name: drink_sum

Description: Total InDUC score

Variable Type: Integer

Value Range:

Coding: NA

Measurement Unit: days

Timepoints: Baseline, 3 Months

Data Processing Details: sum of the physical, interpersonal, social, intrapersonal, and impulse domains of the InDUC instrument.

NDA Element Name: drinc_tis

NDA Data Dictionary: The Drinker Inventory of Consequences (drinc01)

Necessary data transformations to share data with NDA: NA

### audit1

Variable Name: audit1

Description: How often during the last year do you have a drink containing alcohol?

Variable Type: Integer

Value Range: 0;1;2;3;4

Coding: 0 = Never;1 = Monthly or less;2 = 2 to 4 times a month;3 = 2 to 3 times a week;4 = 4 4 or more times a week

Measurement Unit: NA

Timepoints: Baseline, 3 Months

Data Processing Details: the AUDIT variables were summed to calculate the AUDIT score (Saunders JB, Aasland OG, Babor TF, de la Fuente JR, Grant M. Addiction. 1993)

NDA Element Name: audit1

NDA Data Dictionary: Alcohol and Drug Use Disorders Identification Test (audit01)

Necessary data transformations to share data with NDA: "Never" was coded as 0, "Monthly or less" was coded as 6, "2 to 4 times a month" was coded as 2, "2 to 3 times a week" was coded as 3, and "4 or more times a week" was coded as 4.

### audit2

Variable Name: audit2

Description: How many drinks containing alcohol do you have on a typical day when you are drinking during the last year?

Variable Type: Integer

Value Range: 0;1;2;3;4

Coding: 0 = 1 or 2;1 = 3 or 4;2 = 5 or 6;3 = 7 to 9;4 = 10 or more

Measurement Unit: NA

Timepoints: Baseline, 3 Months

Data Processing Details: the AUDIT variables were summed to calculate the AUDIT score (Saunders JB, Aasland OG, Babor TF, de la Fuente JR, Grant M. Addiction. 1993)

NDA Element Name: audit2

NDA Data Dictionary: Alcohol and Drug Use Disorders Identification Test (audit01)

Necessary data transformations to share data with NDA: values set as "1 or 2" have been re-labeled as 6. Those labeled "3 or 4" were re-labeled as 1. Entries labeled "5 or 6" have been changed to 2. The values "7 to 9" were re-labeled as 3, and those that were previously labeled "10 or more" have been changed to 4.

### audit3

Variable Name: audit3

Description: How often during the last year do you have six or more drinks on one occasion?

Variable Type: Integer

Value Range: 0;1;2;3;4

Coding: 0 = Never;1 = Less than monthly;2 = Monthly;3 =Weekly;4 = Daily or almost daily

Measurement Unit: NA

Timepoints: Baseline, 3 Months

Data Processing Details: the AUDIT variables were summed to calculate the AUDIT score (Saunders JB, Aasland OG, Babor TF, de la Fuente JR, Grant M. Addiction. 1993)

NDA Element Name: audit3

NDA Data Dictionary: Alcohol and Drug Use Disorders Identification Test (audit01)

Necessary data transformations to share data with NDA: "Never" have been re-labeled as 0. Those that read "Less than monthly" were changed to 1. Entries previously labeled "Monthly" have been re-labeled as 2. The "Weekly" label has been changed to 3, and entries previously indicating "Daily/ almost daily" have been re-labeled as 4.

### audit4

Variable Name: audit4

Description: How often during the last year have you found that you were not able to stop drinking once you had started?

Variable Type: Integer

Value Range: 0;1;2;3;4

Coding: 0 = Never;1 = Less than monthly;2 = Monthly;3 =Weekly;4 = Daily or almost daily

Measurement Unit: NA

Timepoints: Baseline, 3 Months

Data Processing Details: the AUDIT variables were summed to calculate the AUDIT score (Saunders JB, Aasland OG, Babor TF, de la Fuente JR, Grant M. Addiction. 1993)

NDA Element Name: audit5

NDA Data Dictionary: Alcohol and Drug Use Disorders Identification Test (audit01)

Necessary data transformations to share data with NDA: "Never" have been re-labeled as 0. Those that read "Less than monthly" were changed to 1. Entries previously labeled "Monthly" have been re-labeled as 2. The "Weekly" label has been changed to 3, and entries previously indicating "Daily/ almost daily" have been re-labeled as 4.

### audit5

Variable Name: audit5

Description: How often during the last year have you failed to do what was normally expected of you because of drinking?

Variable Type: Integer

Value Range: 0;1;2;3;4

Coding: 0 = Never;1 = Less than monthly;2 = Monthly;3 =Weekly;4 = Daily or almost daily

Measurement Unit: NA

Timepoints: Baseline, 3 Months

Data Processing Details: the AUDIT variables were summed to calculate the AUDIT score (Saunders JB, Aasland OG, Babor TF, de la Fuente JR, Grant M. Addiction. 1993)

NDA Element Name: audit6

NDA Data Dictionary: Alcohol and Drug Use Disorders Identification Test (audit01)

Necessary data transformations to share data with NDA: "Never" have been re-labeled as 0. Those that read "Less than monthly" were changed to 1. Entries previously labeled "Monthly" have been re-labeled as 2. The "Weekly" label has been changed to 3, and entries previously indicating "Daily/ almost daily" have been re-labeled as 4.

### audit6

Variable Name: audit6

Description: How often during the last year have you needed a first drink in the morning to get yourself going after a heavy drinking session?

Variable Type: Integer

Value Range: 0;1;2;3;4

Coding: 0 = Never;1 = Less than monthly;2 = Monthly;3 =Weekly;4 = Daily or almost daily

Measurement Unit: NA

Timepoints: Baseline, 3 Months

Data Processing Details: the AUDIT variables were summed to calculate the AUDIT score (Saunders JB, Aasland OG, Babor TF, de la Fuente JR, Grant M. Addiction. 1993)

NDA Element Name: audit9

NDA Data Dictionary: Alcohol and Drug Use Disorders Identification Test (audit01)

Necessary data transformations to share data with NDA: "Never" have been re-labeled as 0. Those that read "Less than monthly" were changed to 1. Entries previously labeled "Monthly" have been re-labeled as 2. The "Weekly" label has been changed to 3, and entries previously indicating "Daily/ almost daily" have been re-labeled as 4.

### audit7

Variable Name: audit7

Description: How often during the last year have you had a feeling of guilt or remorse after drinking?

Variable Type: Integer

Value Range: 0;1;2;3;4

Coding: 0 = Never;1 = Less than monthly;2 = Monthly;3 =Weekly;4 = Daily or almost daily

Measurement Unit: NA

Timepoints: Baseline, 3 Months

Data Processing Details: the AUDIT variables were summed to calculate the AUDIT score (Saunders JB, Aasland OG, Babor TF, de la Fuente JR, Grant M. Addiction. 1993)

NDA Element Name: audit10

NDA Data Dictionary: Alcohol and Drug Use Disorders Identification Test (audit01)

Necessary data transformations to share data with NDA: "Never" have been re-labeled as 0. Those that read "Less than monthly" were changed to 1. Entries previously labeled "Monthly" have been re-labeled as 2. The "Weekly" label has been changed to 3, and entries previously indicating "Daily/ almost daily" have been re-labeled as 4.

### audit8

Variable Name: audit8

Description: How often during the last year have you been unable to remember what happened the night before because of your drinking?

Variable Type: Integer

Value Range: 0;1;2;3;4

Coding: 0 = Never;1 = Less than monthly;2 = Monthly;3 =Weekly;4 = Daily or almost daily

Measurement Unit: NA

Timepoints: Baseline, 3 Months

Data Processing Details: the AUDIT variables were summed to calculate the AUDIT score (Saunders JB, Aasland OG, Babor TF, de la Fuente JR, Grant M. Addiction. 1993)

NDA Element Name: audit4

NDA Data Dictionary: Alcohol and Drug Use Disorders Identification Test (audit01)

Necessary data transformations to share data with NDA: "Never" have been re-labeled as 0. Those that read "Less than monthly" were changed to 1. Entries previously labeled "Monthly" have been re-labeled as 2. The "Weekly" label has been changed to 3, and entries previously indicating "Daily/ almost daily" have been re-labeled as 4.

### audit9_final

Variable Name: audit9_final

Description: How often during the last year have you failed to do what was normally expected from you because of drinking (e.g., missed deadlines, poor classroom or work attendance, failed committee responsibilities, inconsistent work patterns)?

Variable Type: Integer

Value Range: 0;2;4

Coding: 0 = No; 2 = Yes, but not in the last year; 4 = Yes, during the last year

Measurement Unit: NA

Timepoints: Baseline, 3 Months

Data Processing Details: the AUDIT variables were summed to calculate the AUDIT score (Saunders JB, Aasland OG, Babor TF, de la Fuente JR, Grant M. Addiction. 1993)

NDA Element Name: audit7

NDA Data Dictionary: Alcohol and Drug Use Disorders Identification Test (audit01)

Necessary data transformations to share data with NDA: "No" have been re-labeled as 0. Entries reading "Yes, but not in the last year" have been changed to 2. Additionally, entries that were previously labeled "Yes, during the last year" have been re-labeled as 4.

### audit10

Variable Name: audit10

Description: Has a relative, friend, doctor, or other health care worker been concerned about your drinking or suggested you cut down?

Variable Type: Integer

Value Range: 0;2;4

Coding: 0 = No;2 = Yes, but not in the last year;4 = Yes, during the last year

Measurement Unit: NA

Timepoints: Baseline, 3 Months

Data Processing Details: the AUDIT variables were summed to calculate the AUDIT score (Saunders JB, Aasland OG, Babor TF, de la Fuente JR, Grant M. Addiction. 1993)

NDA Element Name: audit8

NDA Data Dictionary: Alcohol and Drug Use Disorders Identification Test (audit01)

Necessary data transformations to share data with NDA: "Never" have been re-labeled as 0. Those that read "Less than monthly" were changed to 1. Entries previously labeled "Monthly" have been re-labeled as 2. The "Weekly" label has been changed to 3, and entries previously indicating "Daily/ almost daily" have been re-labeled as 4.

### auditover8

Variable Name: auditover8

Description: Was AUDIT 8 or greater?

Variable Type: Integer

Value Range: 0;1

Coding: 0 = No; 1 = Yes

Measurement Unit: NA

Timepoints: Baseline, 3 Months

Data Processing Details: if the value of AUDIT score is 8 or higher, the corresponding entry in the auditover8 column is set to 1. If the value is less than 8, the corresponding entry in the auditover8 is set to 0.

NDA Element Name: auditdx_t1

NDA Data Dictionary: Alcohol and Drug Use Disorders Identification Test (audit01)

Necessary data transformations to share data with NDA: if the value of AUDIT score is 8 or higher, the corresponding entry in the auditover8 column is set to 1. If the value is less than 8, the corresponding entry in the auditover8 is set to 0.

### audit_sum

Variable Name: audit_sum

Description: Sum of Alcohol Use Items

Variable Type: Integer

Value Range:

Coding: NA

Measurement Unit: NA

Timepoints: Baseline, 3 Months

Data Processing Details: Sum of Alcohol Use Items.

NDA Element Name: alcq

NDA Data Dictionary: Alcohol and Drug Use Disorders Identification Test (audit01)

Necessary data transformations to share data with NDA: NA

### phq1

Variable Name: phq1

Description: Over the past 2 weeks, how often have you bothered by: Little interest or pleasure in doing things

Variable Type: Integer

Value Range: 0;1;2;3

Coding: 0 = Not at all; 1 = Some days; 2 = More than half the days; 3 = Nearly every day

Measurement Unit: NA

Timepoints: Baseline, 3 Months

Data Processing Details: The PHQ variables were summed to get the PHQ9 score (Spitzer RL, Kroenke K, Williams JB. JAMA. 1999)

NDA Element Name: anhedonia

NDA Data Dictionary: Patient Health Questionnaire (phq01)

Necessary data transformations to share data with NDA: "Not at all" have been coded as 0. Those labeled "Some days" have been coded as 1. Entries labeled "More than half the days" are coded as 2, those labeled "Nearly every day" have been coded as 3, and those missing are coded as 999.

### phq2

Variable Name: phq2

Description: Over the past 2 weeks, how often have you bothered by: Feeling down, depressed, or hopeless

Variable Type: Integer

Value Range: 0;1;2;3

Coding: 0 = Not at all; 1 = Some days; 2 = More than half the days; 3 = Nearly every day

Measurement Unit: NA

Timepoints: Baseline, 3 Months

Data Processing Details: The PHQ variables were summed to get the PHQ9 score (Spitzer RL, Kroenke K, Williams JB. JAMA. 1999)

NDA Element Name: down

NDA Data Dictionary: Patient Health Questionnaire (phq01)

Necessary data transformations to share data with NDA: "Not at all" have been coded as 0. Those labeled "Some days" have been coded as 1. Entries labeled "More than half the days" are coded as 2, those labeled "Nearly every day" have been coded as 3, and those missing are coded as 999.

### phq3

Variable Name: phq3

Description: Over the past 2 weeks, how often have you bothered by: Trouble falling or staying asleep or sleeping too much

Variable Type: Integer

Value Range: 0;1;2;3

Coding: 0 = Not at all; 1 = Some days; 2 = More than half the days; 3 = Nearly every day

Measurement Unit: NA

Timepoints: Baseline, 3 Months

Data Processing Details: The PHQ variables were summed to get the PHQ9 score (Spitzer RL, Kroenke K, Williams JB. JAMA. 1999)

NDA Element Name: sleep_trouble

NDA Data Dictionary: Patient Health Questionnaire (phq01)

Necessary data transformations to share data with NDA: "Not at all" have been coded as 0. Those labeled "Some days" have been coded as 1. Entries labeled "More than half the days" are coded as 2, those labeled "Nearly every day" have been coded as 3, and those missing are coded as 999.

### phq4

Variable Name: phq4

Description: Over the past 2 weeks, how often have you bothered by: Feeling tired or having little energy

Variable Type: Integer

Value Range: 0;1;2;3

Coding: 0 = Not at all; 1 = Some days; 2 = More than half the days; 3 = Nearly every day

Measurement Unit: NA

Timepoints: Baseline, 3 Months

Data Processing Details: The PHQ variables were summed to get the PHQ9 score (Spitzer RL, Kroenke K, Williams JB. JAMA. 1999)

NDA Element Name: phq_tired

NDA Data Dictionary: Patient Health Questionnaire (phq01)

Necessary data transformations to share data with NDA: "Not at all" have been coded as 0. Those labeled "Some days" have been coded as 1. Entries labeled "More than half the days" are coded as 2, those labeled "Nearly every day" have been coded as 3, and those missing are coded as 999.

### phq5

Variable Name: phq5

Description: Over the past 2 weeks, how often have you bothered by: Poor appetite or overeating

Variable Type: Integer

Value Range: 0;1;2;3

Coding: 0 = Not at all; 1 = Some days; 2 = More than half the days; 3 = Nearly every day

Measurement Unit: NA

Timepoints: Baseline, 3 Months

Data Processing Details: The PHQ variables were summed to get the PHQ9 score (Spitzer RL, Kroenke K, Williams JB. JAMA. 1999)

NDA Element Name: appetite

NDA Data Dictionary: Patient Health Questionnaire (phq01)

Necessary data transformations to share data with NDA: "Not at all" have been coded as 0. Those labeled "Some days" have been coded as 1. Entries labeled "More than half the days" are coded as 2, those labeled "Nearly every day" have been coded as 3, and those missing are coded as 999.

### phq6

Variable Name: phq6

Description: Over the past 2 weeks, how often have you bothered by: Feeling bad about yourself - or that you are a failure or have let yourself or your family down

Variable Type: Integer

Value Range: 0;1;2;3

Coding: 0 = Not at all; 1 = Some days; 2 = More than half the days; 3 = Nearly every day

Measurement Unit: NA

Timepoints: Baseline, 3 Months

Data Processing Details: The PHQ variables were summed to get the PHQ9 score (Spitzer RL, Kroenke K, Williams JB. JAMA. 1999)

NDA Element Name: failure

NDA Data Dictionary: Patient Health Questionnaire (phq01)

Necessary data transformations to share data with NDA: "Not at all" have been coded as 0. Those labeled "Some days" have been coded as 1. Entries labeled "More than half the days" are coded as 2, those labeled "Nearly every day" have been coded as 3, and those missing are coded as 999.

### phq7

Variable Name: phq7

Description: Over the past 2 weeks, how often have you bothered by: Trouble concentrating on things, such as reading the newspaper or watching television

Variable Type: Integer

Value Range: 0;1;2;3

Coding: 0 = Not at all; 1 = Some days; 2 = More than half the days; 3 = Nearly every day

Measurement Unit: NA

Timepoints: Baseline, 3 Months

Data Processing Details: The PHQ variables were summed to get the PHQ9 score (Spitzer RL, Kroenke K, Williams JB. JAMA. 1999)

NDA Element Name: concentration_problems

NDA Data Dictionary: Patient Health Questionnaire (phq01)

Necessary data transformations to share data with NDA: "Not at all" have been coded as 0. Those labeled "Some days" have been coded as 1. Entries labeled "More than half the days" are coded as 2, those labeled "Nearly every day" have been coded as 3, and those missing are coded as 999.

### phq8

Variable Name: phq8

Description: Over the past 2 weeks, how often have you bothered by: Moving or speaking so slowly that other people could have noticed? Or the opposite - being so fidgety or restless that you have been moving around a lot more than usual

Variable Type: Integer

Value Range: 0;1;2;3

Coding: 0 = Not at all; 1 = Some days; 2 = More than half the days; 3 = Nearly every day

Measurement Unit: NA

Timepoints: Baseline, 3 Months

Data Processing Details: The PHQ variables were summed to get the PHQ9 score (Spitzer RL, Kroenke K, Williams JB. JAMA. 1999)

NDA Element Name: psychomotor_retardation

NDA Data Dictionary: Patient Health Questionnaire (phq01)

Necessary data transformations to share data with NDA: "Not at all" have been coded as 0. Those labeled "Some days" have been coded as 1. Entries labeled "More than half the days" are coded as 2, those labeled "Nearly every day" have been coded as 3, and those missing are coded as 999.

### phq9

Variable Name: phq9

Description: Over the past 2 weeks, how often have you: Thoughts that you would be better off dead or of hurting yourself in some way

Variable Type: Integer

Value Range: 0;1;2;3

Coding: 0 = Not at all; 1 = Some days; 2 = More than half the days; 3 = Nearly every day

Measurement Unit: NA

Timepoints: Baseline, 3 Months

Data Processing Details: The PHQ variables were summed to get the PHQ9 score (Spitzer RL, Kroenke K, Williams JB. JAMA. 1999)

NDA Element Name: si_hi

NDA Data Dictionary: Patient Health Questionnaire (phq01)

Necessary data transformations to share data with NDA: "Not at all" have been coded as 0. Those labeled "Some days" have been coded as 1. Entries labeled "More than half the days" are coded as 2, those labeled "Nearly every day" have been coded as 3, and those missing are coded as 999.

### phq_sum

Variable Name: phq_sum

Description: the PHQ9 score

Variable Type: Integer

Value Range: 0::27

Coding:

Measurement Unit: NA

Timepoints: Baseline, 3 Months

Data Processing Details: The PHQ variables were summed to get the PHQ9 score (Spitzer RL, Kroenke K, Williams JB. JAMA. 1999)

NDA Element Name: phq, xphq

NDA Data Dictionary: Patient Health Questionnaire (phq01)

Necessary data transformations to share data with NDA: The PHQ variables were summed to get the PHQ9 score

### practid

Variable Name: practid

Description: Patient study id

Variable Type: Integer

Value Range: 1000::9550

Coding:

Measurement Unit: NA

Timepoints: Baseline, 3 Months

Data Processing Details:

NDA Element Name: data structure not yet defined

NDA Data Dictionary: data structure not yet defined

Necessary data transformations to share data with NDA: NA

###

### drink_q_1d

Variable Name: drink_q_1d

Description: Yesterday, how many standard alcoholic drinks did you have?

Variable Type: Float

Value Range: 0::25

Coding:

Measurement Unit: NA

Timepoints: Baseline, 3 Months

Data Processing Details: the variables for the number of daily drinks in the last 28 days were used to create the drinking outcomes: binge-drinking days, number of drinks and drinking days

NDA Element Name: data structure not yet defined

NDA Data Dictionary: data structure not yet defined

Necessary data transformations to share data with NDA: NA

### drink_q_2d

Variable Name: drink_q_2d

Description: 2 days ago, how many standard alcoholic drinks did you have?

Variable Type: Float

Value Range: 0::25

Coding:

Measurement Unit: NA

Timepoints: Baseline, 3 Months

Data Processing Details: the variables for the number of daily drinks in the last 28 days were used to create the drinking outcomes: binge-drinking days, number of drinks and drinking days

NDA Element Name: data structure not yet defined

NDA Data Dictionary: data structure not yet defined

Necessary data transformations to share data with NDA: NA

### drink_q_3d

Variable Name: drink_q_3d

Description: 3 days ago, how many standard alcoholic drinks did you have?

Variable Type: Float

Value Range: 0::25

Coding:

Measurement Unit: NA

Timepoints: Baseline, 3 Months

Data Processing Details: the variables for the number of daily drinks in the last 28 days were used to create the drinking outcomes: binge-drinking days, number of drinks and drinking days

NDA Element Name: data structure not yet defined

NDA Data Dictionary: data structure not yet defined

Necessary data transformations to share data with NDA: NA

### drink_q_4d

Variable Name: drink_q_4d

Description: 4 days ago, how many standard alcoholic drinks did you have?

Variable Type: Float

Value Range: 0::25

Coding:

Measurement Unit: NA

Timepoints: Baseline, 3 Months

Data Processing Details: the variables for the number of daily drinks in the last 28 days were used to create the drinking outcomes: binge-drinking days, number of drinks and drinking days

NDA Element Name: data structure not yet defined

NDA Data Dictionary: data structure not yet defined

Necessary data transformations to share data with NDA: NA

### drink_q_5d

Variable Name: drink_q_5d

Description: 5 days ago, how many standard alcoholic drinks did you have?

Variable Type: Float

Value Range: 0::25

Coding:

Measurement Unit: NA

Timepoints: Baseline, 3 Months

Data Processing Details: the variables for the number of daily drinks in the last 28 days were used to create the drinking outcomes: binge-drinking days, number of drinks and drinking days

NDA Element Name: data structure not yet defined

NDA Data Dictionary: data structure not yet defined

Necessary data transformations to share data with NDA: NA

### drink_q_6d

Variable Name: drink_q_6d

Description: 6 days ago, how many standard alcoholic drinks did you have?

Variable Type: Float

Value Range: 0::25

Coding:

Measurement Unit: NA

Timepoints: Baseline, 3 Months

Data Processing Details: the variables for the number of daily drinks in the last 28 days were used to create the drinking outcomes: binge-drinking days, number of drinks and drinking days

NDA Element Name: data structure not yet defined

NDA Data Dictionary: data structure not yet defined

Necessary data transformations to share data with NDA: NA

### drink_q_7d

Variable Name: drink_q_7d

Description: 7 days ago, how many standard alcoholic drinks did you have?

Variable Type: Float

Value Range: 0::25

Coding:

Measurement Unit: NA

Timepoints: Baseline, 3 Months

Data Processing Details: the variables for the number of daily drinks in the last 28 days were used to create the drinking outcomes: binge-drinking days, number of drinks and drinking days

NDA Element Name: data structure not yet defined

NDA Data Dictionary: data structure not yet defined

Necessary data transformations to share data with NDA: NA

### drink_q_8d

Variable Name: drink_q_8d

Description: 8 days ago, how many standard alcoholic drinks did you have?

Variable Type: Float

Value Range: 0::25

Coding:

Measurement Unit: NA

Timepoints: Baseline, 3 Months

Data Processing Details: the variables for the number of daily drinks in the last 28 days were used to create the drinking outcomes: binge-drinking days, number of drinks and drinking days

NDA Element Name: data structure not yet defined

NDA Data Dictionary: data structure not yet defined

Necessary data transformations to share data with NDA: NA

### drink_q_9d

Variable Name: drink_q_9d

Description: 9 days ago, how many standard alcoholic drinks did you have?

Variable Type: Float

Value Range: 0::25

Coding:

Measurement Unit: NA

Timepoints: Baseline, 3 Months

Data Processing Details: the variables for the number of daily drinks in the last 28 days were used to create the drinking outcomes: binge-drinking days, number of drinks and drinking days

NDA Element Name: data structure not yet defined

NDA Data Dictionary: data structure not yet defined

Necessary data transformations to share data with NDA: NA

### drink_q_10d

Variable Name: drink_q_10d

Description: 10 days ago, how many standard alcoholic drinks did you have?

Variable Type: Float

Value Range: 0::25

Coding:

Measurement Unit: NA

Timepoints: Baseline, 3 Months

Data Processing Details: the variables for the number of daily drinks in the last 28 days were used to create the drinking outcomes: binge-drinking days, number of drinks and drinking days

NDA Element Name: data structure not yet defined

NDA Data Dictionary: data structure not yet defined

Necessary data transformations to share data with NDA: NA

### drink_q_11d

Variable Name: drink_q_11d

Description: 11 days ago, how many standard alcoholic drinks did you have?

Variable Type: Float

Value Range: 0::25

Coding:

Measurement Unit: NA

Timepoints: Baseline, 3 Months

Data Processing Details: the variables for the number of daily drinks in the last 28 days were used to create the drinking outcomes: binge-drinking days, number of drinks and drinking days

NDA Element Name: data structure not yet defined

NDA Data Dictionary: data structure not yet defined

Necessary data transformations to share data with NDA: NA

### drink_q_12d

Variable Name: drink_q_12d

Description: 12 days ago, how many standard alcoholic drinks did you have?

Variable Type: Float

Value Range: 0::25

Coding:

Measurement Unit: NA

Timepoints: Baseline, 3 Months

Data Processing Details: the variables for the number of daily drinks in the last 28 days were used to create the drinking outcomes: binge-drinking days, number of drinks and drinking days

NDA Element Name: data structure not yet defined

NDA Data Dictionary: data structure not yet defined

Necessary data transformations to share data with NDA: NA

drink_q_13d

Variable Name: drink_q_13d

Description: 13 days ago, how many standard alcoholic drinks did you have?

Variable Type: Float

Value Range: 0::25

Coding:

Measurement Unit: NA

Timepoints: Baseline, 3 Months

Data Processing Details: the variables for the number of daily drinks in the last 28 days were used to create the drinking outcomes: binge-drinking days, number of drinks and drinking days

NDA Element Name: data structure not yet defined

NDA Data Dictionary: data structure not yet defined

Necessary data transformations to share data with NDA: NA

### drink_q_14d

Variable Name: drink_q_14d

Description: 14 days ago, how many standard alcoholic drinks did you have?

Variable Type: Float

Value Range: 0::25

Coding:

Measurement Unit: NA

Timepoints: Baseline, 3 Months

Data Processing Details: the variables for the number of daily drinks in the last 28 days were used to create the drinking outcomes: binge-drinking days, number of drinks and drinking days

NDA Element Name: data structure not yet defined

NDA Data Dictionary: data structure not yet defined

Necessary data transformations to share data with NDA: NA

### drink_q_15d

Variable Name: drink_q_15d

Description: 15 days ago, how many standard alcoholic drinks did you have?

Variable Type: Float

Value Range: 0::25

Coding:

Measurement Unit: NA

Timepoints: Baseline, 3 Months

Data Processing Details: the variables for the number of daily drinks in the last 28 days were used to create the drinking outcomes: binge-drinking days, number of drinks and drinking days

NDA Element Name: data structure not yet defined

NDA Data Dictionary: data structure not yet defined

Necessary data transformations to share data with NDA: NA

### drink_q_16d

Variable Name: drink_q_16d

Description: 16 days ago, how many standard alcoholic drinks did you have?

Variable Type: Float

Value Range: 0::25

Coding:

Measurement Unit: NA

Timepoints: Baseline, 3 Months

Data Processing Details: the variables for the number of daily drinks in the last 28 days were used to create the drinking outcomes: binge-drinking days, number of drinks and drinking days

NDA Element Name: data structure not yet defined

NDA Data Dictionary: data structure not yet defined

Necessary data transformations to share data with NDA: NA

### drink_q_17d

Variable Name: drink_q_17d

Description: 17 days ago, how many standard alcoholic drinks did you have?

Variable Type: Float

Value Range: 0::25

Coding:

Measurement Unit: NA

Timepoints: Baseline, 3 Months

Data Processing Details: the variables for the number of daily drinks in the last 28 days were used to create the drinking outcomes: binge-drinking days, number of drinks and drinking days

NDA Element Name: data structure not yet defined

NDA Data Dictionary: data structure not yet defined

Necessary data transformations to share data with NDA: NA

### drink_q_18d

Variable Name: drink_q_18d

Description: 18 days ago, how many standard alcoholic drinks did you have?

Variable Type: Float

Value Range: 0::25

Coding:

Measurement Unit: NA

Timepoints: Baseline, 3 Months

Data Processing Details: the variables for the number of daily drinks in the last 28 days were used to create the drinking outcomes: binge-drinking days, number of drinks and drinking days

NDA Element Name: data structure not yet defined

NDA Data Dictionary: data structure not yet defined

Necessary data transformations to share data with NDA: NA

### drink_q_19d

Variable Name: drink_q_19d

Description: 19 days ago, how many standard alcoholic drinks did you have?

Variable Type: Float

Value Range: 0::25

Coding:

Measurement Unit: NA

Timepoints: Baseline, 3 Months

Data Processing Details: the variables for the number of daily drinks in the last 28 days were used to create the drinking outcomes: binge-drinking days, number of drinks and drinking days

NDA Element Name: data structure not yet defined

NDA Data Dictionary: data structure not yet defined

Necessary data transformations to share data with NDA: NA

### drink_q_20d

Variable Name: drink_q_20d

Description: 20 days ago, how many standard alcoholic drinks did you have?

Variable Type: Float

Value Range: 0::25

Coding:

Measurement Unit: NA

Timepoints: Baseline, 3 Months

Data Processing Details: the variables for the number of daily drinks in the last 28 days were used to create the drinking outcomes: binge-drinking days, number of drinks and drinking days

NDA Element Name: data structure not yet defined

NDA Data Dictionary: data structure not yet defined

Necessary data transformations to share data with NDA: NA

### drink_q_21d

Variable Name: drink_q_21d

Description: 21 days ago, how many standard alcoholic drinks did you have?

Variable Type: Float

Value Range: 0::25

Coding:

Measurement Unit: NA

Timepoints: Baseline, 3 Months

Data Processing Details: the variables for the number of daily drinks in the last 28 days were used to create the drinking outcomes: binge-drinking days, number of drinks and drinking days

NDA Element Name: data structure not yet defined

NDA Data Dictionary: data structure not yet defined

Necessary data transformations to share data with NDA: NA

### drink_q_22d

Variable Name: drink_q_22d

Description: 22 days ago, how many standard alcoholic drinks did you have?

Variable Type: Float

Value Range: 0::25

Coding:

Measurement Unit: NA

Timepoints: Baseline, 3 Months

Data Processing Details: the variables for the number of daily drinks in the last 28 days were used to create the drinking outcomes: binge-drinking days, number of drinks and drinking days

NDA Element Name: data structure not yet defined

NDA Data Dictionary: data structure not yet defined

Necessary data transformations to share data with NDA: NA

### drink_q_23d

Variable Name: drink_q_23d

Description: 23 days ago, how many standard alcoholic drinks did you have?

Variable Type: Float

Value Range: 0::25

Coding:

Measurement Unit: NA

Timepoints: Baseline, 3 Months

Data Processing Details: the variables for the number of daily drinks in the last 28 days were used to create the drinking outcomes: binge-drinking days, number of drinks and drinking days

NDA Element Name: data structure not yet defined

NDA Data Dictionary: data structure not yet defined

Necessary data transformations to share data with NDA: NA

### drink_q_24d

Variable Name: drink_q_24d

Description: 24 days ago, how many standard alcoholic drinks did you have?

Variable Type: Float

Value Range: 0::25

Coding:

Measurement Unit: NA

Timepoints: Baseline, 3 Months

Data Processing Details: the variables for the number of daily drinks in the last 28 days were used to create the drinking outcomes: binge-drinking days, number of drinks and drinking days

NDA Element Name: data structure not yet defined

NDA Data Dictionary: data structure not yet defined

Necessary data transformations to share data with NDA: NA

### drink_q_25d

Variable Name: drink_q_25d

Description: 25 days ago, how many standard alcoholic drinks did you have?

Variable Type: Float

Value Range: 0::25

Coding:

Measurement Unit: NA

Timepoints: Baseline, 3 Months

Data Processing Details: the variables for the number of daily drinks in the last 28 days were used to create the drinking outcomes: binge-drinking days, number of drinks and drinking days

NDA Element Name: data structure not yet defined

NDA Data Dictionary: data structure not yet defined

Necessary data transformations to share data with NDA: NA

### drink_q_26d

Variable Name: drink_q_26d

Description: 26 days ago, how many standard alcoholic drinks did you have?

Variable Type: Float

Value Range: 0::25

Coding:

Measurement Unit: NA

Timepoints: Baseline, 3 Months

Data Processing Details: the variables for the number of daily drinks in the last 28 days were used to create the drinking outcomes: binge-drinking days, number of drinks and drinking days

NDA Element Name: data structure not yet defined

NDA Data Dictionary: data structure not yet defined

Necessary data transformations to share data with NDA: NA

### drink_q_27d

Variable Name: drink_q_27d

Description: 27 days ago, how many standard alcoholic drinks did you have?

Variable Type: Float

Value Range: 0::25

Coding:

Measurement Unit: NA

Timepoints: Baseline, 3 Months

Data Processing Details: the variables for the number of daily drinks in the last 28 days were used to create the drinking outcomes: binge-drinking days, number of drinks and drinking days

NDA Element Name: data structure not yet defined

NDA Data Dictionary: data structure not yet defined

Necessary data transformations to share data with NDA: NA

### drink_q_28d

Variable Name: drink_q_28d

Description: 28 days ago, how many standard alcoholic drinks did you have?

Variable Type: Float

Value Range: 0::25

Coding:

Measurement Unit: NA

Timepoints: Baseline, 3 Months

Data Processing Details: the variables for the number of daily drinks in the last 28 days were used to create the drinking outcomes: binge-drinking days, number of drinks and drinking days

NDA Element Name: data structure not yet defined

NDA Data Dictionary: data structure not yet defined

Necessary data transformations to share data with NDA: NA

### binge.days

Variable Name: binge.days

Description: Number of binge-drinking days in the last 28 days

Variable Type: Integer

Value Range:

Coding: NA

Measurement Unit: days

Timepoints: Baseline, 3 Months

Data Processing Details: the variables for the number of daily standard drinks in the last 28 days (i.e. drink_q_xxd) were summed to calculate the binge-drinking days, number of drinks and drinking days

NDA Element Name: data structure not yet defined

NDA Data Dictionary: data structure not yet defined

Necessary data transformations to share data with NDA: NA

### drinking.amount

Variable Name: drinking.amount

Description: Number of standard drinks the individual consumed in the last 28 days

Variable Type: Integer

Value Range:

Coding: NA

Measurement Unit: number of standard drinks

Timepoints: Baseline, 3 Months

Data Processing Details: the variables for the number of daily standard drinks in the last 28 days (i.e. drink_q_xxd) were summed to calculate the binge-drinking days, number of drinks and drinking days

NDA Element Name: data structure not yet defined

NDA Data Dictionary: data structure not yet defined

Necessary data transformations to share data with NDA: NA

### drinking.days

Variable Name: drinking.days

Description: Number of drinking days in the last 28 days

Variable Type: Integer

Value Range:

Coding: NA

Measurement Unit: number of standard drinks

Timepoints: Baseline, 3 Months

Data Processing Details: the variables for the number of daily standard drinks in the last 28 days (i.e. drink_q_xxd) were summed to calculate the binge-drinking days, number of drinks and drinking days

NDA Element Name: data structure not yet defined

NDA Data Dictionary: data structure not yet defined

Necessary data transformations to share data with NDA: NA
