# Supplementary material for: Effectiveness of a brief intervention and text-based booster in the emergency department to reduce harmful and hazardous alcohol use: A pragmatic randomized adaptive clinical trial in Moshi, Tanzania
Source: PLoS Med. 2025 Oct 27;22(10):e1004548. doi: 10.1371/journal.pmed.1004548 (PMC12578324; doi:10.1371/journal.pmed.1004548)
Supplement: Attachment — Submitted filename: Answer to reviewers comments.docx [file pmed.1004548.s012.docx]

## Comments from the editorial team:

Please note that, for further consideration, it is essential to address the reviewers' concerns to their satisfaction.

The editorial team agrees with Reviewer #2 that the self-reported outcome should be presented as a major limitation in both the Discussion and the Abstract. In addition, when revising the manuscript, please thoroughly document the statistical methods in a way that would allow others to replicate your findings.

R: Thank you for your comments. We agree with the importance of clearly acknowledging the limitations of self-reported outcomes. We have revised the abstract and rephrased the relevant section of the Limitations to present this as a key limitation. We have also updated the statistical appendix to provide a more detailed description of our analytic approach. The authors are committed towards reproducibility and have also shared the analytical code in a public GitHub repository and continue to support transparency by expanding the supplementary materials accordingly.

## Comments from the reviewers:

### Reviewer 2

Reviewer #2: This manuscript has been improved from the prior version. However, many of my original concerns remain, and there are a few additional that need clarification.

**Major concerns:**

1.	Self-reported outcomes: As reiterated by multiple reviewers, there is the potential for social desirability bias, and it could be differential due to non-blinding of participants. The manuscript still says in the limitations, "Social desirability may have influenced overall reporting levels, but such bias is likely to have been similar across both arms." There is no basis for this. The fact that secondary endpoints were not statistically significant doesn't mean there was no social desirability bias in the primary endpoint. Due to its potential for impacting the primary findings, this limitation needs to be clearly acknowledged in the limitations section and, I would argue, the abstract.

R: Thank you for this important comment. We agree that social desirability bias is an important limitation, and we acknowledge that its potential impact on the primary outcome cannot be ruled out. In response, we have revised the abstract and reworded the Limitations section to more clearly highlight the reliance on self-reported data as a key limitation of the study.

2.	Missing Data/Multiple imputation: MI was pre-specified in the protocol, and thus should be the primary analysis presented in the main text, not as a sensitivity analysis. 10 imputations seems low to me - can the authors explain how this was chosen? Furthermore, the MI approach should result in an estimate of the same endpoint as is currently presented for the primary outcome, not just a table of model coefficients as in Table S4. For the description of missing data, the authors conclude that missingness is consistent with a MAR mechanism. MNAR cannot be distinguished from MAR in tables of observed covariates, so this statement is much too strong. They can say instead that MCAR was ruled out and that they will assume MAR based on a given set of covariates. Covariates used in the MI model need to be listed.

R: Thank you for the thorough comment, it helped us understand a weakness in our reporting and allowed us to improve how we describe our analysis. We understand the rationale behind reporting the MI models as primary in the manuscript and have adjusted accordingly. Our past choice of reporting the complete case was due to our initial MAR assumption. However, we appreciate the thorough comment about the MAR assumption being too strong and agree with the reviewers comments. We also agree that reporting the MI as the primary results is consistent with the prespecified plan and makes the analysis reporting more rigorous. We have revised the manuscript to present the multiple imputation analysis as the primary analysis in the main text (Table 3, Figure 2), consistent with our pre-specified plan. These results, including model-predicted binge drinking days and difference-in-difference estimates with corresponding confidence intervals, are now clearly reported in the Results section and supporting figures. We moved the complete case analysis results to the Supplementary Material Table S4.

We agree with the reviewer that increasing the number of imputed datasets improves the stability and reliability of the results. To address this, we generated 100 imputed datasets. For each dataset, up to 50 iterations were performed to ensure convergence of the imputation model, following an initial 100 burn-in iterations. These steps were taken to enhance the robustness of the multiple imputation procedure and the resulting model estimates. We have revised and improved the Statistical Appendix and Methods section. We have also rephrased our description of the missing data assessment. In the original manuscript, we stated that the data were consistent with a missing at random (MAR) mechanism. We now acknowledge that missing not at random (MNAR) cannot be ruled out based solely on observed covariates and that the assumption of MAR is based on the inclusion of relevant predictors in the imputation model. We have updated the Methods section accordingly, clarifying that we ruled out missing completely at random (MCAR) and assumed MAR conditional on observed covariates.

To enhance clarity and reproducibility, we have expanded the statistical appendix to include detailed descriptions of the modeling approaches used across all outcomes, including the specification of models, the treatment of time as a categorical variable, and the covariates used in each component of the models. We also provide a complete list of covariates included in the multiple imputation model. Finally, we note that we have shared the full analysis code in a public GitHub repository and continue to adhere to principles of open and reproducible science by expanding the supplementary materials in this revision.

3.	Sensitivity Analysis: I maintain that the analytic methods are unnecessarily complicated and introduce unnecessary parametric assumptions. They are also susceptible to overinterpreting the intervention due to outliers. I recommend that the authors do include the more straightforward, less assumption dependent, and less prone to the influence of outliers Wilcoxon results (median, 95% CI) as an added sensitivity analysis.

R: Thank you for your comment. We have added the results from the Wilcoxon rank-sum test as an additional sensitivity analysis. The full set of results, including versions with and without statistical outliers and across both complete case and imputed datasets, is presented in Supplementary Table S7. We have also added explicit references to this table in the main text to improve visibility and clarity. We belive that this addition has made the reporting of these results more robust, showing a consistency accorss analytical approaches and sensitivity evaluations.

4.	Detailed statistical methods need to be described. For each model, authors need to say what covariates were included. For zero-inflated models, there is a model for both the probability of being an excess zero and a model for the mean count given that one is not an excess zero. Covariates in each need to be described. How was time incorporated in each longitudinal model (e.g., as a linear term)? In general, authors should say what variables were included in each model and how overall estimates were obtained from the models. As previously suggested, a detailed statistical appendix would improve clarity/interpretability of all analyses. The current appendix is just a list of R packages, and the SAP itself does not provide these details. The SAP even includes a different endpoint than is reported in the paper (odds ratios).

R: Thank you for your comment. We have added detailed model specifications to the statistical appendix to improve clarity and reproducibility. For each model, we now explicitly describe the covariates used, including both the count and zero-inflation components where applicable. For the primary outcome of binge drinking days, we used a zero-inflated negative binomial model implemented with the glmmTMB package, in R. The count component included fixed effects for time (modeled as a categorical indicator for 3-month follow-up) and its interaction with treatment assignment, with baseline as the reference. The zero-inflation component included the treatment group as a predictor, modeling the probability of excess zeros. We used a similar approach for secondary outcomes. Drinking days and drinking amount were modeled with negative binomial mixed-effects models (no zero-inflation), and continuous outcomes including DrInC, AUDIT, and PHQ-9 scores were modeled using linear mixed-effects models via the lmerTest package, with the same structure of fixed effects and a random intercept for participant to account for within-subject correlation. No additional covariates were included in the outcome models themselves. Estimates of treatment effects were obtained from model-predicted values, comparing within-group change from baseline to follow-up and calculating the difference-in-differences between arms. Confidence intervals and p-values were derived using non-parametric bootstrap resampling with 1,000 iterations per imputed dataset. Complete details on model specification, covariate structure, and estimation procedures are now included in the revised Statistical Appendix and Supplementary Files, along with links to the GitHub repository containing all analysis code. As part of our sensitivity analysis, we conducted alternative model fits, including models without the zero inflation, negative binomial, the generalized Poisson regression models and non-parametric Wilcoxon rank-sum tests. These were performed to assess the robustness of our findings under different modeling assumptions. Results remained directionally consistent across these approaches, reinforcing the validity of the observed treatment effects. We report these results as part of our sesitivity analysis. Our final choice was to use the pre-specified modelling approach in the main analysis, as it provided the best fit for the data given the distributional characteristics of the outcome particularly the high variance and excess zeros observed for binge drinking days.

As for the SAP, the version submitted with the previous revision does state under Section 3 (“Statistical Methods”) that:

*“Our previous observational data had shown that alcohol consumption data is overdispersed and inflated with the presence of zeros, and therefore we will use a zero-inflated negative binomial method with a log link function to model count outcomes. For AUDIT scores, depression, and alcohol-related harms, linear models will be used.”*

We acknowledge that the table skeleton included at the end of the SAP lists hazard ratios and odds ratios, which may be misleading. This was a holdover from an earlier draft and does not reflect the modeling approach actually used in the analyses. We regret the inconsistency and have revised the SAP appendix to align with the final analytic approach described in the manuscript.

5.	Influence of outliers: I share a concern voiced by other reviewers. The difference could potentially be driven by a few outliers. To see if this is the case, I recommend four things. (1) add the proportion of participants with 1+ binge drinking days to table 2, (2) add the max for each outcome in table 2 (in addition to Q1/Q3/mean/median), (3) in Figure 2, as previously recommended, add individual level data, like via a scatterplot in the background of the bars, and (4) include the sensitivity analysis recommended above.

R: Thank you for your constructive suggestions. In order to address the points you raised: (1) we added the proportion of participants reporting at least one binge drinking day at each time point to Table 2, as requested. (2) the maximum value for each outcome has now been included in Table 2, alongside the existing summary statistics (Q1, Q3, mean, and median).; (3) As suggested, we added a version of Figure 2 with individual data points overlaid (via scatterplot) to the sensitivity analysis. To maintain clarity in the main text and avoid overloading Figure 2, we have included this updated figure in the Supplementary File (Figure S1), where it is referenced alongside the outlier sensitivity analysis. (4) We have conducted the recommended sensitivity analysis by excluding participants with binge drinking values beyond 1.5 times the interquartile range from the first and third quartiles (i.e., extreme outliers). The results are now included in Table S5 and described in the main text. As can be noted by the results presented, outliers have an impact on the average estimate, but the results are still consisten with the direction and magnitute of the effect..

6.	Use of p-values: The "statistical significance" language needs to be toned down. Per guidance from the American Statistical Association's statement on the recommended use of p-values ([https://nam11.safelinks.protection.outlook.com/?url=https%3A%2F%2Fjournals.lww.com%2Fjoacp%2Ffulltext%2F2016%2F32040%2Fthe_american_statistical_association_statement_on.1.aspx&data=05%7C02%7Ccatherine.lynch%40duke.edu%7C1f2867741ca74e44fcc508ddbe2b4cc2%7Ccb72c54e4a314d9eb14a1ea36dfac94c%7C0%7C0%7C638875815361996305%7CUnknown%7CTWFpbGZsb3d8eyJFbXB0eU1hcGkiOnRydWUsIlYiOiIwLjAuMDAwMCIsIlAiOiJXaW4zMiIsIkFOIjoiTWFpbCIsIldUIjoyfQ%3D%3D%7C0%7C%7C%7C&sdata=JhwMWji00OaJiOz0q%2Fao9R7AV3GBzHz1qHOxH%2FpsScg%3D&reserved=0](https://journals.lww.com/joacp/fulltext/2016/32040/the_american_statistical_association_statement_on.1.aspx)), I recommend only discussing statistical significance for outcomes that were powered. For secondary outcomes, the "no significant differences" language should be removed, and instead a meaningful comparison of differences with CIs should be discussed. Furthermore, statistical testing should not be conducted for missing data analysis. The study was not powered for these comparisons, so p-values are not meaningful.

R: We agree with the reviewer’s recommendation and have made changes throughout the manuscript to align with the American Statistical Association’s guidance on the interpretation of p-values. Specifically, we have: a) removed p-values from the analysis of missing data and replaced statistical testing with descriptive summaries; b) reframed language related to secondary outcomes to avoid the focus on statistical tests and instead described the estimated differences along with their confidence intervals; c) clarified that these secondary outcomes were not powered for formal hypothesis testing and should be interpreted as exploratory. We believe these changes better reflect the uncertainty in our estimates and enhance the interpretability and transparency of our findings.

7.	Participant Drop out. Participants should state in the text that data for the primary outcome was only available for 68% of intervention participants and 71% in usual care so that readers don't have to calculate this from Table 1.

R: We have included language in the first paragraph of the results section to acknowledge these numbers.

**Other comments:**

1.	In the response to reviewer 2, comment 3c, the authors say that "However, we had defined to conduct a more robust model that would allow us to estimate the more accurate estimate adjusting for baseline confounders." I would emphasize that there is no confounding in a truly randomized clinical trial (unless you mean informative missing data). Adjustments for baseline covariates are for the purposes of efficiency gain, not bias reduction.

R: Thank you for this clarification. We agree that, in a randomized controlled trial, confounding is minimized by design. We previously included that language to reflect an earlier reviewer’s suggestion and acknowledge that our wording may have overstated the rationale.

2.	In author summary, authors should summarize both significant findings as well as secondary outcomes that were similar between arms.

R: Thank you for your comment. We have edited the Author Summary to account for those similarities.

### Reviewer 3

Reviewer #3: The authors made a great work to answer reviewers' points. Most points were adequately treated (in particular presentation of the design) and the resulting manuscript is highly enhanced.

There are however a few important points that remain unclear.

1. It is not clear how the authors handled time in their analyses. It looks like they used both baseline adjustment and change scores (difference between T1 and T2). If authors used a change score, they should not adjust this for baseline value, since this information would be used twice.

R: We thank the reviewer for this important observation. To clarify, we did not use change scores as outcome variables. Instead, we modeled binge drinking days as a count outcome using a zero-inflated negative binomial regression, with time (baseline vs. 3-month follow-up), treatment assignment, and their interaction included as covariates. Specifically, the model included indicators for the follow-up time point (period3mo) and the interaction between follow-up and treatment group, allowing us to estimate the differential effect of the intervention at follow-up compared to baseline. This approach aligns with a longitudinal constrained model, where baseline values are treated as outcomes within the model rather than covariates. This method improves efficiency and avoids the pitfalls of adjusting for baseline in change-score models, such as introducing bias or redundancy. We have improved our Statistical Appendix to describe our approach.

2. The "difference in differences" are sometimes highy significant while the confidence intervals include 0. How is this possible? Either results are incorrect or it raises high doubts the quality of the data.

R: Thank you for pointing out this concern. Indeed the “difference-in-differences” values reported in the main text are based on model-predicted means, with confidence intervals derived from non-parametric bootstrap resampling of those predictions. The corresponding p-values are calculated from the model coefficient estimates. These inference approaches are not mathematically identical and can show minor variations. We did evaluate the confidence interval of the model estimated (effect) and observed a consistent confidence interval that did not include 0. This result (the Multiple Imputation model-based pooled coefficient estimates and their 95% confidence intervals) we included into the Supplementary Material. These model-based intervals do not cross the null value and align with the reported p-values.We decided to report these results to be consistent with our pre-specified analytical approach. However, the direction and magnitude of the effect of the intervention in both the primary and secondary outcomes were thoroughly evaluated in sensitivity analysis, producing consistent results. In particular, when analyzing the data with non-parametric methods, and with a less restrictive Generalized Poisson model, we did observe consistent confidence intervals (S9 and S10 - drinking days; and S12 and S13 for drinking amount). The models based on the negative binomial assumption did show that minor variation.

We present the results from the generalized Poisson, negative binomial, and zero-inflated negative binomial models as well as Kruskal-Wallis test in the observed data in the Supplementary Material (Tables S9-S13) to allow readers to assess the robustness of our findings across different modeling assumptions.

3. The flow chart should include detials on exclusion criteria.

R: Thank you for your comment. We have updated the caption for Figure 1 to clarify the alcohol-related inclusion criteria. Participants were eligible if they met at least one of the specified criteria related to alcohol use.Data on information criteria is also available in the Supplementary Material (Table S1).

## Technical Requests:

1) Please insert an Ethics Statement at the beginning of your Methods section, under a subheading 'Ethics Statement'. It must include:

i) A statement that formal consent was obtained (must state whether verbal/written) OR the reason consent was not obtained (e.g. anonymity). NOTE: If child participants, the statement must declare that formal consent was obtained from the parent/guardian.

R: This heading and statement were added to the methods section.
