# Supplementary material for: Effectiveness of a brief intervention and text-based booster in the emergency department to reduce harmful and hazardous alcohol use: A pragmatic randomized adaptive clinical trial in Moshi, Tanzania
Source: PLoS Med. 2025 Oct 27;22(10):e1004548. doi: 10.1371/journal.pmed.1004548 (PMC12578324; doi:10.1371/journal.pmed.1004548)
Supplement: Attachment — Submitted filename: Response to Reviewers_.docx [file pmed.1004548.s013.docx]

Comments from Reviewers:

## Reviewer 2

Reviewer #2: The authors have done an excellent job responding to the previous reviews. My only comment is that it would be good to provide a high-level summary of the sensitivity analyses for the primary outcome in the main text rather than just pointing to them in the supplement.

R: We appreciate the suggestion to include a high-level summary of the sensitivity analyses for the primary outcome in the main text. We have revised the Results to add this summary.

## Reviewer 3

1. Reviewer #3: The revisions carried out by the authors have satisfactorily addressed reviewers' comments.

Here are a few last minor points.

2. In the discussion, the sentence « Our study demonstrates the effectiveness of a culturally adapted and pragmatic implementation of a brief intervention with specific intervention fidelity focus for injury patients" should be toned down, taking into account study limitations and design.

Same comment for the first sentence of the Conclusions.

R Thank you for the suggestion. We revised the Discussion and Conclusions to tone down the language, addressing your comment.

3. The number of decimals in some reported numbers is unusually long. Refer to journal guidelines, but numbers (e.g. p values) are usually rounded to two decimals.

R: Thank you for the suggestion. We standardized p-values to two decimal places in Table 3 and the Results. For very small p-values, we retained higher precision (typically 3–5 decimals) to avoid rounding to 0.00 and to convey the magnitude accurately (e.g., p=0.0023), which we felt would be clearer than reporting 0.00. We also preferred to report in this way to increase verification of the result found.

4. As the adaptative design may be less familiar to many readers, the conclusions of the abstract and the conclusions of the article should mention further stages of the adaptative design.

R: A brief implication of the results on the adaptive study design was added to the abstract and conclusions.

## Requests from Editors:

GENERAL

1. Please note that we require authors to include page and line numbers in their manuscript for review purposes.

R: We have added page and line numbers

2. Please confirm that your title complies with to PLOS Medicine's style. Your title must be nondeclarative and not a question. It should begin with main concept if possible. "Effect of" should be used only if causality can be inferred, i.e., for an RCT. Please place the study design ("A randomized controlled trial," "A retrospective study," "A modelling study," etc.) in the subtitle (i.e., after a colon).

Suggestion: Effectiveness of a Brief Intervention and Text-Based Booster in the Emergency Department to Reduce Harmful and Hazardous Alcohol Use: A Pragmatic Randomized Adaptive Clinical Trial in Moshi, Tanzania

R: We have replaced our title to follow PLOS Medicine's style recommendations.

3. Statistical reporting: Please revise throughout the manuscript, including tables and figures.

- Please report statistical information as follows to improve clarity for the reader ""XX (95% CI [XX,XX]; p</=)"".

- Please separate upper and lower bounds with commas instead of hyphens as the latter can be confused with reporting of negative values.

- Please repeat statistical definitions (HR, CI etc.) for each set of parentheses.

R: We have made all the changes suggested in the main text.

4. Please ensure that all abbreviations are defined at first use throughout the text (including statistical abbreviations). Please also check figures and tables.

R: We have revised the main text and made the changes necessary.

5. Please ensure that tables and figures, including those in supplementary files, are appropriately referenced in the main text.

R: We have revised the main text and made the changes necessary.

6. Please check that any use of statistical terms (such as trend or significant) are supported by the data, and if not please remove them.

R: We have revised the main text and made the changes necessary.

7. The Data Availability Statement (DAS) following the main text (“Data Sharing”) and the DAS in the online submission form differ. Please check and ensure to provide the correct DAS in the online submission form. Please note that PLOS Medicine requires that the de-identified data underlying the specific results in a published article be made available, without restrictions on access, in a public repository or as Supporting Information at the time of article publication, provided it is legal and ethical to do so.

R: We have revised the “Data Sharing” section in the main text and the DAS in the online submission form for uniformity.

8. Please revise for use of patient-centered language. Please note that patient-centered language is constructed with the use of post-modified nouns (e.g. 'patients with injury’ (or similar) instead of ‘injury patients’) putting the person first in the sentence structure.

R: We have revised the main text to address this comment.

ABSTRACT

9. Please confirm that your abstract complies with our requirements, including providing all the information relevant to this study type [https://nam11.safelinks.protection.outlook.com/?url=https%3A%2F%2Fjournals.plos.org%2Fplosmedicine%2Fs%2Fsubmission-guidelines%23loc-abstract&data=05%7C02%7Ccatherine.lynch%40duke.edu%7Ccacaca1f55794341aa8408ddf1df9f9a%7Ccb72c54e4a314d9eb14a1ea36dfac94c%7C0%7C0%7C638932664943415116%7CUnknown%7CTWFpbGZsb3d8eyJFbXB0eU1hcGkiOnRydWUsIlYiOiIwLjAuMDAwMCIsIlAiOiJXaW4zMiIsIkFOIjoiTWFpbCIsIldUIjoyfQ%3D%3D%7C0%7C%7C%7C&sdata=JknkiGTz9O9xQ05coSlJ4RUaMtSDeLyRByrCylYRpqo%3D&reserved=0](https://journals.plos.org/plosmedicine/s/submission-guidelines#loc-abstract)

R: We have revised our abstract and confirm that our abstract complies with the requirements.

10. Per CONSORT, please note that only the primary outcome of the trial should be reported in your Abstract. Secondary outcomes should only be included in the Abstract if all secondary outcomes are fully reported. For trials that have many secondary outcomes, the Abstract should be limited to reporting the primary outcome.

R: We have edited the abstract to only cover primary outcome results.

11. “We report the results of a superiority clinical trial comparing the effectiveness of a culturally adapted brief intervention, “Punguza Pombe Kwa Afya Yako” (PPKAY, Reduce Alcohol for your Health), with text-based boosters to usual care.” – Please note that the final sentence should clearly state the study question.

R: We revised the final sentence of the Abstract’s Background to explicitly state our study hypothesis.

12. “This manuscript will report…” – please change tense.

R: We changed this and other occurrences.

13. Please clearly state the primary outcome of the trial.

R: We edited the last sentence of the Methods and findings section to clearly state our primary outcome

14. Please include years during which the study took place.

R: We added a sentence to the Methods and Findings section.

15. Please state that analysis was intention to treat.

R: We added a sentence to the Methods and Findings section.

16. Please provide the number of participants lost to follow up in each group.

R: We added a sentence to the Methods and Findings section.

17. Please include baseline characteristics of the study population (e.g. mean age, sex).

R: We added a sentence to the Methods and Findings section.

18. Please ensure that all numbers presented in the abstract are present and identical to numbers presented in the main manuscript text.

R: We made the changes in the Abstract when appropriate.

19. Please change ‘Interpretation’ to ‘Conclusions’.

R: We made the changes in the Abstract.

20. In the last sentence of the Abstract Methods and Findings section, please describe the main limitation(s) of the study's methodology.

R: The main limitations were previously addressed in the Conclusion section of the Abstract. We have moved the limitations to the Methods and Findings section of the Abstract

AUTHOR SUMMARY

21. In the author summary, in the final bullet point of 'What Do These Findings Mean?', please include the main limitations of the study in non-technical language (as a separate bullet point).

R: We made the changes in the “What Do These Findings Mean?” Section of the Author Summary.

22. We suggest removing the information on secondary outcomes and focusing only on the primary outcome. This would streamline the author summary.

R: We made the changes in the Author Summary to address this comment.

METHODS AND RESULTS

23. Thank you for providing your CONSORT checklist. Please replace the page numbers with paragraph numbers per section (e.g. "Methods, paragraph 1"), since the page numbers of the final published paper may be different from the page numbers in the current manuscript. Also, please ensure to use the CONSORT 2025 checklist.

R: New 2025 CONSORT Checklist was included, and references section and paragraph numbers as suggested.

24. “…but will be presented in brief below and in the Supplementary Information (S2 File).” – please ensure to add clear labels to the files. Currently, there’s no file labelled as S2 file.

R: We have edited the file names and mentions throughout the text to ensure consistency in numbering of the Supplementary Material

25. Since the trial focuses on the PPKAY intervention, we suggest providing more information about the intervention (e.g., under "Brief Intervention") rather than relying on references 19 and 26. We want readers to find information about the cultural adaptations in the main text.

R: Information about cultural adaptation was added to the intervention description.

26. Please confirm that the primary and secondary outcomes are reported in accordance with the trial protocol, and indicate any changes in the main text, if applicable.

R: We have added a sentence to the “Outcome Measures” section of our manuscript to address the minor changes between the primary outcomes from the published protocol and the version used in this manuscript.

27. Please include the statement on code in the data availability statement.

R: We revised the text to address that.

28. Please confirm that changes in the analysis-- including those made in response to peer review comments—have been identified as such in the Methods section of the paper, with rationale. If not, please revise accordingly.

R: We revised the text and made sure that all changes were addressed.

29. You state that the current manuscript presents the second interim analysis with 65% enrollment. However, according to your sample size calculation, the second interim analysis should include approximately 320 participants. However, it seems that you had already included 448 participants by that time. Please comment and clarify.

R: Agreed, we planned for the interim analysis whenwhen we 65% complete follow up was reached. Our initial manuscript submitted to the journal reported this complete case analysis as the main analysis for this same reason. However, as pointed by the reviewers, we reported the analysis with all patients completing the FUP window using the multiple imputation approach. Thus the difference in the %. We edited the methods to reflect that we conducted the interim analysis when 65% of the complete FUP was reached.

30. “The primary and secondary outcome results presented in this study represent data imputed using mice.” – please capitalize ‘mice’. If used for the first time, please define the abbreviation.

R: We have made changes to fix this issue throughout the main text.

31. Table 1/2: Please clarify that the intervention group is a combination of personalized and standard booster messages.

R: We added footnotes to the tables to address this issue.

32. “Across all evaluations, results were directionally consistent, with greater reductions in binge drinking days observed in the intervention group.” – based on Figure S1, we recommend toning down this statement and providing a more accurate description of the results.

R: We have made changes to this and other similar sentences throughout the main text.

33. Figure 2: Why aren't you using a box plot with single data points, as shown in Figure S1? We believe that box plots are more informative and transparent. Also, when a p-value is provided, please include the statistical test used to determine it in the legend.

R: Thank you for the comment. Agreed that the boxplot figure is more informative and transparent. We added the boxplot to the figure. We also decided to keep the bar graph to facilitate the comprehension for readers not as versed in reading boxplots and data distributions.

34. Table 3: When a p value is given, please specify the statistical test used to determine it in the legend.

R: We added footnotes to Table 3 to address this issue.

35. Due to the large standard deviations for the primary and secondary outcomes and the rarity of the outcome(s), clearly state that there is a high degree of uncertainty.

R: We added a statement to state this uncertainty in the first paragraph of the discussion.

36. Tables: Please specify the variables controlled for in all relevant Tables.

R: The analysis reported were not controlled for additional baseline characteristics. We had initially provided these analysis as sensitivity, but this results were dropped during one of the revisions cycles, as per reviewers suggestion, given the baseline characteristics.

DISCUSSION

37. Pleas remove all subheadings, including the ‘Conclusion’ subheading.

R: We have removed all unnecessary subheadings.

38. When revising the discussion, please consider toning down any conclusions given the rarity of your primary outcome and the lack of a comparator group.

R: We have revised the discussion based on the reviewers comments to tone down the conclusions based on the rarity of the primary outcome and the lack of the comparator group.

39. ”Our pragmatic randomized adaptive clinical trial has demonstrated effectiveness of our culturally- adapted brief intervention, Punguza Pombe Kwa Afya Yako (PPKAY) with mobile health-based boosters reduces, on average, 1.2 binge drinking days per 4 weeks compared to usual care.” – We believe this should say: “Our pragmatic, randomized, adaptive clinical trial demonstrated the effectiveness of our culturally adapted brief intervention, Punguza Pombe Kwa Afya Yako (PPKAY). Compared to usual care, PPKAY with mobile health-based boosters reduced binge drinking by an average of 1.2 days more per four weeks.”

R: We agree with this suggestion and have made changes to the section in the text.

------------------------------------------------------------

General Editorial Requests

1) We ask every co-author listed on the manuscript to fill in a contributing author statement. If any of the co-authors have not filled in the statement, we will remind them to do so when the paper is revised. If all statements are not completed in a timely fashion this could hold up the re-review process. Should there be a problem getting one of your co-authors to fill in a statement we will be in contact. YOU MUST NOT ADD OR REMOVE AUTHORS UNLESS YOU HAVE ALERTED THE EDITOR HANDLING THE MANUSCRIPT TO THE CHANGE AND THEY SPECIFICALLY HAVE AGREED TO IT.

R: Acknowledged.

2) Please ensure that the paper adheres to the PLOS Data Availability Policy (see [https://nam11.safelinks.protection.outlook.com/?url=http%3A%2F%2Fjournals.plos.org%2Fplosmedicine%2Fs%2Fdata-availability&data=05%7C02%7Ccatherine.lynch%40duke.edu%7Ccacaca1f55794341aa8408ddf1df9f9a%7Ccb72c54e4a314d9eb14a1ea36dfac94c%7C0%7C0%7C638932664943432150%7CUnknown%7CTWFpbGZsb3d8eyJFbXB0eU1hcGkiOnRydWUsIlYiOiIwLjAuMDAwMCIsIlAiOiJXaW4zMiIsIkFOIjoiTWFpbCIsIldUIjoyfQ%3D%3D%7C0%7C%7C%7C&sdata=IonB3vvNebCdfGmonFvZazFLRPJ3hJEDaouqptQRXgc%3D&reserved=0)](http://journals.plos.org/plosmedicine/s/data-availability), which requires that all data underlying the study's findings be provided in a repository or as Supporting Information. For data residing with a third party, authors are required to provide instructions with contact information for obtaining the data. PLOS journals do not allow statements supported by "data not shown" or "unpublished results." For such statements, authors must provide supporting data or cite public sources that include it.

R: Acknowledged.

3) Please review your reference list to ensure that it is complete and correct. If you have cited papers that have been retracted, please include the rationale for doing so in the manuscript text, or remove these references and replace them with relevant current references. Any changes to the reference list should be mentioned in the rebuttal letter that accompanies your revised manuscript.

R: We have revised the reference list, but no changes were needed.
