# Supplementary material for: Effectiveness of a brief intervention and text-based booster in the emergency department to reduce harmful and hazardous alcohol use: A pragmatic randomized adaptive clinical trial in Moshi, Tanzania
Source: PLoS Med. 2025 Oct 27;22(10):e1004548. doi: 10.1371/journal.pmed.1004548 (PMC12578324; doi:10.1371/journal.pmed.1004548)
Supplement: Attachment — Submitted filename: Response to Reviewer Comments update.pdf [file pmed.1004548.s011.pdf]

To whom it may concern:

Thank you for the opportunity to receive this excellent feedback and to revise this manuscript. We have reviewed each and every piece of feedback suggested by both the academic editor and the reviewers. We have created the following point-by-point response to the feedback and made relevant changes to the manuscript to improve it based on this constructive feedback.

General editorial requests:

1. We ask every co-author listed on the manuscript to fill in a contributing author statement, making sure to declare all competing interests. If any of the co-authors have not filled in the statement, we will remind them to do so when the paper is revised. If all statements are not completed in a timely fashion this could hold up the re-review process. If new competing interests are declared later in the revision process, this may also hold up the submission. Should there be a problem getting one of your co-authors to fill in a statement we will be in contact. Please do not add or remove authors without first discussing this with the handling editor. You can see our competing interests policy here: https://nam11.safelinks.protection.outlook.com/?url=http%3A%2F%2Fjournals.plos.org%2Fplosmedicine%2Fs%2Fcompeting-interests&data=05%7C02%7Ccatherine.lynch%40duke.edu%7C30f63cd06c024d409b9f08dd57d397c1%7Ccb72c54e4a314d9eb14a1ea36dfac94c%7C0%7C0%7C638763288486881004%7CUnknown%7CTWFpbGZsb3d8eyJFbXB0eU1hcGkiOnRydWUsIlYiOiIwLjAuMDAwMCIsIlAiOiJXaW4zMiIsIkFOIjoiTWFpbCIsIldUIjoyfQ%3D%3D%7C0%7C%7C%7C&sdata=9GuTuyiQJdIOIQrERxukAXgq15CiQOcvbPHyuRt5KfM%3D&reserved=0.
   All authors have been reminded to confirm authorship and declare interests.

2. Please upload any figures associated with your paper as individual TIF or EPS files with 300dpi resolution at resubmission; please read our figure guidelines for more information on our requirements: https://nam11.safelinks.protection.outlook.com/?url=http%3A%2F%2Fjournals.plos.org%2Fplosmedicine%2Fs%2Ffigures&data=05%7C02%7Ccatherine.lynch%40duke.edu%7C30f63cd06c024d409b9f08dd57d397c1%7Ccb72c54e4a314d9eb14a1ea36dfac94c%7C0%7C0%7C638763288486895907%7CUnknown%7CTWFpbGZsb3d8eyJFbXB0eU1hcGkiOnRydWUsIlYiOiIwLjAuMDAwMCIsIlAiOiJXaW4zMiIsIkFOIjoiTWFpbCIsIldUIjoyfQ%3D%3D%7C0%7C%7C%7C&sdata=VwunuMYIohn9vRvbuNaYOEMOI9rk01G41CGI3MQ2hck%3D&reserved=0. While revising your submission, please upload your figure files to the PACE digital diagnostic tool, https://nam11.safelinks.protection.outlook.com/?url=https%3A%2F%2Fpacev2.apexcovantage.com%2F&data=05%7C02%7Ccatherine.lynch%40duke.edu%7C30f63cd06c024d409b9f08dd57d397c1%7Ccb72c54e4a314d9eb14a1ea36dfac94c%7C0%7C0%7C638763288486913851%7CUnknown%7CTWFpbGZsb3d8eyJFbXB0eU1hcGkiOnRydWUsIlYiOiIwLjAuMDAwMCIsIlAiOiJXaW4zMiIsIkFOIjoiTWFpbCIsIldUIjoyfQ%3D%3D%7C0%7C%7C%7C&sdata=hRbIwnqxkW1UK68GkDhxyygV1bgjvNFMHZom3oRnrKc%3D&reserved=0. PACE helps ensure that figures meet PLOS requirements. To use PACE, you must first register as a user. Then, login and navigate to the UPLOAD tab, where you will find detailed instructions on how to use the tool. If you encounter any issues or have any questions when using PACE, please email us at PLOSMedicine@plos.org.
   Figures were uploaded in the format requested

3. Please ensure that the paper adheres to the PLOS Data Availability Policy (see https://nam11.safelinks.protection.outlook.com/?url=http%3A%2F%2Fjournals.plos.org%2Fplosmedicine%2Fs%2Fdata-availability&data=05%7C02%7Ccatherine.lynch%40duke.edu%7C30f63cd06c024d409b9f08dd57d397c1%7Ccb72c54e4a314d9eb14a1ea36dfac94c%7C0%7C0%7C638763288486935599%7CUnknown%7CTWFpbGZsb3d8eyJFbXB0eU1hcGkiOnRydWUsIlYiOiIwLjAuMDAwMCIsIlAiOiJXaW4zMiIsIkFOIjoiTWFpbCIsIldUIjoyfQ%3D%3D%7C0%7C%7C%7C&sdata=IsRhzgqeB6wFqMMCc2sgdm%2B7pSbdf%2B4%2BKcPYq6fzuKg%3D&reserved=0), which requires that all data underlying the study's findings be provided in a repository or as Supporting Information. For data residing with a third party, authors are required to provide instructions with contact information (web or email address) for obtaining the data. Please note that a study author cannot be the contact person for the data. PLOS journals do not allow statements supported by "data not shown" or "unpublished results." For such statements, authors must provide supporting data or cite public sources that include it.

The paper adheres to the PLOS Data Availability Policy

4. We expect all researchers with submissions to PLOS in which author-generated code underpins the findings in the manuscript to make all author-generated code available without restrictions upon publication of the work. In cases where code is central to the manuscript, we may require the code to be made available as a condition of publication. Authors are responsible for ensuring that the code is reusable and well documented. Please make any custom code available, either as part of your data deposition or as a supplementary file. Please add a sentence to your data availability statement regarding any code used in the study, e.g. "The code used in the analysis is available from Github [URL] and archived in Zenodo [DOI link]" Please review our guidelines at https://nam11.safelinks.protection.outlook.com/?url=https%3A%2F%2Fjournals.plos.org%2Fplosmedicine%2Fs%2Fmaterials-software-and-code-sharing&data=05%7C02%7Ccatherine.lynch%40duke.edu%7C30f63cd06c024d409b9f08dd57d397c1%7Ccb72c54e4a314d9eb14a1ea36dfac94c%7C0%7C0%7C638763288486949971%7CUnknown%7CTWFpbGZsb3d8eyJFbXB0eU1hcGkiOnRydWUsIlYiOiIwLjAuMDAwMCIsIlAiOiJXaW4zMiIsIkFOIjoiTWFpbCIsIldUIjoyfQ%3D%3D%7C0%7C%7C%7C&sdata=7t%2BHM%2Bv63USfTXLHCNIICDpg%2FerNiHg4h1DrXEABgXk%3D&reserved=0 and ensure that your code is shared in a way that follows best practice and facilitates reproducibility and reuse. Because Github depositions can be readily changed or deleted, we encourage you to make a permanent DOI'd copy (e.g. in Zenodo) and provide the URL.

   The Github reference has been added to the manuscript.

5. FINANCIAL DISCLOSURES: The funding statement should include: specific grant numbers, initials of authors who received each award, URLs to sponsors' websites. Also, please state whether any sponsors or funders (other than the named authors) played any role in study design, data collection and analysis, the decision to publish, or preparation of the manuscript. If they had no role in the research, include this sentence: "The funders had no role in study design, data collection and analysis, decision to publish, or preparation of the manuscript."

   Financial disclosure was updated to note the recipient and funder website.

6. COMPETING INTERESTS: All authors must declare their relevant competing interests per the PLOS policy, which can be seen here: https://nam11.safelinks.protection.outlook.com/?url=https%3A%2F%2Fjournals.plos.org%2Fplosmedicine%2Fs%2Fcompeting-interests&data=05%7C02%7Ccatherine.lynch%40duke.edu%7C30f63cd06c024d409b9f08dd57d397c1%7Ccb72c54e4a314d9eb14a1ea36dfac94c%7C0%7C0%7C638763288486964954%7CUnknown%7CTWFpbGZsb3d8eyJFbXB0eU1hcGkiOnRydWUsIlYiOiIwLjAuMDAwMCIsIlAiOiJXaW4zMiIsIkFOIjoiTWFpbCIsIldUIjoyfQ%3D%3D%7C0%7C%7C%7C&sdata=gtSIkFdfNWXjsCDT9f8SuEtjHSwGkOyjQF6dpzZILBA%3D&reserved=0

   All authors have been reminded to declare interests.

7. DATA AVAILABILITY: The Data Availability Statement (DAS) requires revision. For each data source used in your study: a) If the data are freely or publicly available, note this and state the location of the data: within the paper, in Supporting Information files, or in a public repository (include the DOI or accession number). b) If the data are owned by a third party but freely available upon request, please note this and state the owner of the data set and contact information for data requests (web or email address). Note that a study author cannot be the contact person for the data. c) If the data are not freely available, please describe briefly the ethical, legal, or contractual restriction that prevents you from sharing it. Please also include an appropriate contact (web or email address) for inquiries (again, this cannot be a study author).

   Data included in the current study (with the exception of participants who opted out of public data sharing) will be made publicly available at the time of manuscript publication within the National Institute on Alcohol Abuse and Alcoholism Data Archive at the following permanent address: https://dx.doi.org/10.15154/jeve-g615

   Data availability statement has been updated accordingly, to read as follows:

   Data used in the preparation of this manuscript (with the exception of participants who opted out of public data sharing) are available from the National Institute on Alcohol Abuse and Alcoholism Data Archive (NIAAADA). NIAAADA is a collaborative informatics system created by

the National Institutes of Health (NIH) to provide a national resource to support the sharing of federally-funded data for accelerating research. Data can be accessed via http://nda.nih.gov/niaaa. Dataset identifier(s): [Collection ID: 3425; Digital Object Identifier (DOI): 10.15154/jeve-g615. This manuscript reflects the views of the authors and may not reflect the opinions or views of the NIH.

FORMATTING - GENERAL

8. Abstract: Please structure your abstract using the PLOS Medicine headings (Background, Methods and Findings, Conclusions). Please combine the Methods and Findings sections into one section.

    Abstract was restructured accordingly.

9. At this stage, we ask that you include a short, non-technical Author Summary of your research to make findings accessible to a wide audience that includes both scientists and non-scientists. The Author Summary should immediately follow the Abstract in your revised manuscript. This text is subject to editorial change and should be distinct from the scientific abstract. Ideally each sub-heading should contain 2-3 single sentence, concise bullet points containing the most salient points from your study. In the final bullet point of 'What Do These Findings Mean?', please include the main limitations of the study in non-technical language. Please see our author guidelines for more information: https://nam11.safelinks.protection.outlook.com/?url=https%3A%2F%2Fjournals.plos.org%2Fplosmedicine%2Fs%2Frevising-your-manuscript%23loc-author-summary&data=05%7C02%7Ccatherine.lynch%40duke.edu%7C30f63cd06c024d409b9f08dd57d397c1%7Ccb72c54e4a314d9eb14a1ea36dfac94c%7C0%7C0%7C638763288486979642%7CUnknown%7CTWFpbGZsb3d8eyJFbXB0eU1hcGkiOnRydWUsIlYiOiIwLjAuMDAwMCIsIlAiOiJXaW4zMiIsIkFOIjoiTWFpbCIsIldUIjoyfQ%3D%3D%7C0%7C%7C%7C&sdata=j%2FqW0Lmcno062En9Ex99ztRy0Dp%2FGt1%2FoDYsQgQoWKk%3D&reserved=0.

    The author summary was included in the revised manuscript:

10. Please express the main results with 95% CIs as well as p values. When reporting p values please report as p<0.001 and where higher as the exact p value p=0.002, for example. Throughout, suggest reporting statistical information as follows to improve clarity for the reader "22% (95% CI [13%,28%]; p</=)". Please be sure to define all numerical values at first use.

    In instances of missing or inexact p-values, those figures were updated.

11. Please include page numbers and line numbers in the manuscript file. Use continuous line numbers (do not restart the numbering on each page).

    Page and line numbers were included

12. Please cite the reference numbers in square brackets. Citations should precede punctuation.

    References were updated accordingly to align with journal style.

FIGURES AND TABLES

13. Please provide titles and legends for all figures and tables (including those in Supporting Information files).

    Titles and legends included as required.

14. Please define all abbreviations used in each figure/table (including those in Supporting Information files).

    Abbreviations were included in each table where previously missing.

15. Please consider avoiding the use of red and green in order to make your figure more accessible to those with color blindness.

    The use of all color has been avoided.

SUPPLEMENTARY MATERIAL: Please note that supplementary material will be posted as supplied by the authors. Therefore, please amend it according to the relevant comments outlined here.

16. Please cite your Supporting Information as outlined here: https://nam11.safelinks.protection.outlook.com/?url=https%3A%2F%2Fjournals.plos.org%2Fplosmedicine%2Fs%2Fsupporting-information&data=05%7C02%7Ccatherine.lynch%40duke.edu%7C30f63cd06c024d409b9f08dd57d397c1%7Ccb72c54e4a314d9eb14a1ea36dfac94c%7C0%7C0%7C638763288486994093%7CUnknown%7CTWFpbGZsb3d8eyJFbXB0eU1hcGkiOnRydWUsIlYiOiIwLjAuMDAwMCIsIlAiOiJXaW4zMiIsIkFOIjoiTWFpbCIsIldUIjoyfQ%3D%3D%7C0%7C%7C%7C&sdata=UAEBBorxkFFcmrzJoQIPULSGzlMQeESqvTJwpoEXeiE%3D&reserved=0

    Where incorrect, supplementary material citations have been updated.

REFERENCES

17. PLOS uses the numbered citation (citation-sequence) method and first six authors, et al.

    All references were updated to comply with PLOS style. Please note that these changes, though completed as requested, will not display as tracked due to the use of citation management software.

18. Please ensure that journal name abbreviations match those found in the National Center for Biotechnology Information (NCBI) databases (https://nam11.safelinks.protection.outlook.com/?url=http%3A%2F%2Fwww.ncbi.nlm.nih.gov%2Fnlmcatalog%2Fjournals&data=05%7C02%7Ccatherine.lynch%40duke.edu%7C30f63cd06c024d409b9f08dd57d397c1%7Ccb72c54e4a314d9eb14a1ea36dfac94c%7C0%7C0%7C638763288487008300%7CUnknown%7CTWFpbGZsb3d8eyJFbXB0eU1hcGkiOnRydWUsIlYiOiIwLjAuMDAwMCIsIlAiOiJXaW4zMiIsIkFOIjoiTWFpbCIsIldUIjoyfQ%3D%3D%7C0%7C%7C%7C&sdata=edDki3x%2BvL5264nd64d7%2FhuZEU9VmtbH5fBMNVa4%2F44%3D&reserved=0), and are appropriately formatted and capitalised.

    All references were updated to comply with PLOS style. As noted above, these changes, though completed as requested, will not display as tracked due to the use of citation management software.

19. Where website addresses are cited, please include the complete URL and specify the date of access (e.g. [accessed: 12/06/2023]).

    All website references were updated to include date of access.

20. Please also see https://nam11.safelinks.protection.outlook.com/?url=https%3A%2F%2Fjournals.plos.org%2Fplosmedicine%2Fs%2Fsubmission-guidelines%23loc-references&data=05%7C02%7Ccatherine.lynch%40duke.edu%7C30f63cd06c024d409b9f08dd57d397c1%7Ccb72c54e4a314d9eb14a1ea36dfac94c%7C0%7C0%7C638763288487022500%7CUnknown%7CTWFpbGZsb3d8eyJFbXB0eU1hcGkiOnRydWUsIlYiOiIwLjAuMDAwMCIsIlAiOiJXaW4zMiIsIkFOIjoiTWFpbCIsIldUIjoyfQ%3D%3D%7C0%7C%7C%7C&sdata=015oYZH0PZrF5FHHuqBl4GVb5EGotMrFFBzCblt779I%3D&reserved=0 for further details on reference formatting.

    All references were updated to comply with PLOS style. As noted above, these changes, though completed as requested, will not display as tracked due to the use of citation management software.

STUDY TYPE-SPECIFIC REQUESTS

21. PLOS Medicine requires that all trials be prospectively registered in one of registries recognized by WHO. Please ensure that study registration details are included in the Methods section.

    Trial registration is included as required.

22. Please structure the Methods section using the following sub-headings: Study design and participants, Randomization and masking, Procedures, Outcomes, Statistical analysis.

    Subheadings in the methods were updated as suggested.

23. Please ensure that all outcomes measures are reported according to the trial protocol [and/or trial registry]. Please clarify and explain all discrepancies between the paper and protocol. If the outcomes were not prespecified in the protocol, please define them in the Methods (Outcomes section) as post hoc and explain why they were added. Post-hoc comparisons should be presented as hypothesis generating rather than conclusive.

Trial protocol and outcomes were clarified and are addressed in the reviewer comments below.

24. Please ensure that all prespecified outcomes (primary, secondary, and exploratory) are listed in the Methods/Outcomes section and indicate whether there are outcomes that are not presented in the current report.

    All primary and secondary outcomes are included in the outcomes subheading.

25. Please specify the dates (Month Day, Year) during which study enrollment and follow up occurred.

    Dates are now included in the updated manuscript.

26. Please include absolute numbers wherever you report percentages; eg, n/N (%)

    All results including percentages now include absolute numbers.

27. Please present the safety data for the study including numbers of specific events and whether or not adverse events are thought to be related to treatment. AEs should be reported in the abstract, per CONSORT and CONSORT-Harms.

    Information on AEs has been added.

28. Please complete the CONSORT checklist (https://nam11.safelinks.protection.outlook.com/?url=https%3A%2F%2Fwww.equator-network.org%2Freporting-guidelines%2Fconsort%2F&data=05%7C02%7Ccatherine.lynch%40duke.edu%7C30f63cd06c024d409b9f08dd57d397c1%7Ccb72c54e4a314d9eb14a1ea36dfac94c%7C0%7C0%7C638763288487037643%7CUnknown%7CTWFpbGZsb3d8eyJFbXB0eU1hcGkiOnRydWUsIlYiOiIwLjAuMDAwMCIsIlAiOiJXaW4zMiIsIkFOIjoiTWFpbCIsIldUIjoyfQ%3D%3D%7C0%7C%7C%7C&sdata=2U4BSHoIvOKgdzdd0B%2FoFhLrtfc2GuqcI5ZnxiJDCI8%3D&reserved=0) and ensure that all components of CONSORT are present in the manuscript, including how randomization was performed, allocation concealment, blinding of intervention, definition of lost to follow-up, power statement. When completing the checklist, please use section and paragraph numbers, rather than page numbers.

    The CONSORT checklist was updated to include line numbers in addition to page numbers.

29. Please report your abstract according to CONSORT for abstracts, following the PLOS Medicine abstract structure (Background, Methods and Findings, Conclusions) https://nam11.safelinks.protection.outlook.com/?url=https%3A%2F%2Fwww.equator-network.org%2Freporting-guidelines%2Fconsort-abstracts%2F&data=05%7C02%7Ccatherine.lynch%40duke.edu%7C30f63cd06c024d409b9f08dd57d397c1%7Ccb72c54e4a314d9eb14a1ea36dfac94c%7C0%7C0%7C638763288487057013%7CUnknown%7CTWFpbGZsb3d8eyJFbXB0eU1hcGkiOnRydWUsIlYiOiIwLjAuMDAwMCIsIlAiOiJXaW4zMiIsIkFOIjoiTWFpbCIsIldUIjoyfQ%3D%3D%7C0%7C%7C%7C&sdata=3ARGx4C0YWCeSvcOM1eqzqQNoW6g0V9YoviiK8w6yHY%3D&reserved=0

    Abstract was updated to use the proper structure, and necessary information was added where previously omitted.

30. If your trial had to undergo important modifications in response to extenuating circumstances, please complete the CONSERVE-CONSORT checklist and provide in your Supporting Information; (https://nam11.safelinks.protection.outlook.com/?url=https%3A%2F%2Fwww.equator-network.org%2Freporting-guidelines%2Fguidelines-for-reporting-trial-protocols-and-completed-trials-modified-due-to-the-covid-19-pandemic-and-other-extenuating-circumstances-the-conserve-2021-statement%2F&data=05%7C02%7Ccatherine.lynch%40duke.edu%7C30f63cd06c024d409b9f08dd57d397c1%7Ccb72c54e4a314d9eb14a1ea36dfac94c%7C0%7C0%7C638763288487076199%7CUnknown%7CTWFpbGZsb3d8eyJFbXB0eU1hcGkiOnRydWUsIlYiOiIwLjAuMDAwMCIsIlAiOiJXaW4zMiIsIkFOIjoiTWFpbCIsIldUIjoyfQ%3D%3D%7C0%7C%7C%7C&sdata=SPYtV7RF3bbv0xmBb7pqsKJVLYxRirAPIvWB18sBkxk%3D&reserved=0). When completing the checklist, please use section and paragraph numbers, rather than page numbers.

    As the protocol was revised prior to study initiation, the modifications are not reported via the CONSERVE-CONSORT Checklist. Details clarifying protocol modification prior to study initiation are addressed directly to reviewer comments below.

31. In keeping with our commitment to Open Science, please include the study protocol document and analysis plan (including any amendments) as Supporting Information to be published with the manuscript if accepted.

    Study protocol and analysis plan are included as supporting information.

32. Please note that PLOS Medicine requires prospective, public registration of a data sharing plan (as part of mandatory clinical trials registration) for all clinical trials that began enrollment on or after January 1, 2019, in accordance with ICMJE requirements.

Academic editor's comments:

In addition to the reviewers' comments, I recommend that authors are asked to make the overall design of their adaptive trial clearer to the reader, and to add a few sentences about the value of adaptive RCTs, as they are not familiar to everyone.

R: This is a great suggestion. We have added more information in the study design to discuss what an adaptive trial is and how it works (lines 147-156).

Reviewer #1 Comments

Reviewer #1: Thank you for the opportunity to review this manuscript. I commend the authors for writing a clear, succinct, and easy to to read manuscript. I have some comments and suggestions.

Major comments

1. Why were the two PPKAY arms (one with standard SMS booster and another with personalized SMS booster) analysed as one group? Of course, I understand that they both received the brief intervention, but why include these two distinct intervention arms if they are not analysed separately? This may be done at a later stage, in another analysis, but its omission here seems ominous.

R: The PRACT study was designed as a two-stage adaptive trial, with Stage 1 (representing the current manuscript) powered to assess the superiority of the intervention (PPKAY + any SMS Booster) compared to usual care at the 3-month follow-up. This interim analysis was pre-specified in the trial protocol as the primary analysis for evaluating the initial effectiveness of the intervention. Further information about the adaptive study design, the Stage 1 and Stage 2 research questions and justification of this process were included in the "*Study Design*" section of the methods (lines 147-156).

2. I would expect the baseline binge drinking days, drinking days, number of drinks etc. to be balanced between groups as baseline, and there is no discussion to hypothesise why this is not the case? Is it a failure of randomization, too small sample size?

R: Thank you for this excellent question. While the averages may seem unbalanced, the distribution of the data is very similar between groups. Variations in baseline assessments in patient reports measures are common in such studies. We had anticipated this could be the case, especially around the counts of drinks that tend to be highly influenced by zeros. In our protocol, we anticipated this variation and had planned to apply a conditional approach to account for this variation. For further assurance, the mean of binge drinking days across arms at baseline (2.2 ± 5.4 for the usual care group, compared to 3 ± 6 for the intervention group), did show a statistically significant difference ($p = 0.08$). Additionally, all other demographics were balanced providing another layer of evidence that the randomization was appropriate for this study.

3. Given the open label nature of the intervention and self reporting of the outcome, there is a big risk of detection bias; those in the intervention arm are more likely to self-report lower alcohol use. The authors state in the limitations that they do not believe the self-reporting practices should differ by intervention arm, but there is no empirical evidence to prove this. One way to address this is to have a negative control outcome, that shares similar determinants of detection as the outcome of interest, but is not expected to be affected by the intervention. For example, this could be something like number of days eating junk food in the last 3 months? Did the authors collect any outcomes they could use as a negative control outcome?

R: Thank you for this thoughtful comment. We did not collect a designated negative control outcome in this study. However, we agree that this is a valuable suggestion and have added it as a limitation in the revised

manuscript. Although we lacked a formal negative control, we note that other self-reported measures collected in the study—such as the AUDIT and DrInC scores—did not show evidence of significant differences between arms, which may suggest that reporting bias was limited. If participants in the intervention arm were systematically underreporting alcohol use due to social desirability or perceived expectations, we might also expect to see corresponding reductions in these related self-report measures, which we did not observe. Nonetheless, we acknowledge that the potential for detection bias remains, particularly given the open-label nature of the trial and reliance on self-reported outcomes. While self-report is a standard method for measuring alcohol use in behavioral trials, including in the U.S. and globally, we acknowledge this limitation and suggest that ours and other future studies should consider the valuable comment of incorporating objective biomarkers or negative control outcomes to further assess and minimize the risk of reporting bias. We have edited the limitation section to make this more clear (lines 484-517).

4. Looking at the results in Table 2, the median binge drinking days is 0 for all groups both at baseline and at 3 months. Similarly, for the other outcomes the median values are very low. Across all outcomes, the mean is considerably larger than the median, which suggests the data are considerably skewed. A way to address this problem would be to define the outcomes as the proportion of people above a certain clinically relevant threshold. For example, the proportion of people with greater than 3 binge drinking days in the last 3 months. The addition of analyses with outcome definitions like above, perhaps as secondary analyses, would greatly improve the paper.
R: Thank you for this thoughtful suggestion. We agree that the data are highly skewed, as reflected by the fact that the median number of binge drinking days is zero across all groups and time points, while the mean values are higher. This pattern is common in alcohol intervention trials, especially those conducted in emergency care settings, where many individuals report little or no alcohol use at follow-up—regardless of intervention—due to contextual factors such as trauma exposure or the acute care experience. This results in a distribution with a high proportion of zeros and a small subset of participants driving the variability in mean values. Given the nature of the data, we selected an analytical approach appropriate for overdispersed and zero-inflated count outcomes. We modeled binge drinking days as a numeric outcome using a zero-inflated negative binomial model and reported predicted means to provide adjusted, model-based estimates that account for the skewed distribution. While categorizing the outcome into clinically relevant thresholds (e.g., number of participants with more than 3 binge drinking days) is a reasonable suggestion, there are currently no widely accepted clinical cutoffs for binge drinking days. This creates a complex paradigm. The WHO suggested no alcohol is safe, while it is also unrealistic to expect that alcohol use is expected to be reduced to zero, especially in countries where alcohol use is widely cultural. Thus, we focused on a measure that captured the riskier alcohol consumption behavior, that has shown to be more impacted in other trials, but that brings the challenges of modeling we encountered. As a result, we opted to retain the full numeric outcome to preserve statistical power and sensitivity to change, acknowledging that even small reductions in drinking days may yield meaningful public health benefits. This approach is consistent with WHO guidance and current international standards in alcohol research.

Minor comments

1. There are too many acronyms used throughout the manuscript, which makes it hard to read in places
R: Thank you, we have reduced the number of acronyms used in the manuscript.

2. Were all the nurses trained on how to deliver the PPKAY? And was that training standardized?
R: The nurses were trained by our research team in regular sessions hosted in the Kilimanjaro Christian Medical Centre. The training sessions were standardized and followed a defined script. Considering the turnover of nurses and healthcare professionals in general in KCMC, our team hosted training sessions on a needs based fashion, with new nurses being trained whenever the demand of interventions increased. An additional citation (published after initial submission) has been referenced (line 213).

3. How many replications were used in the bootstrapping process?
R: One thousand (1,000) replications. We added this to the methods section to make it more clear (lines 301-305).

4. Why are the limitations positioned after the conclusions? Surely the conclusions should be the last part of the manuscript?
R: We agree that the limitations should come before the conclusions. We have edited the manuscript to address that.

5. P-values do not need to be reported to 4 decimal places. 2 is sufficient.
R: Thank you, this was changed.

Reviewer #2 Comments
Reviewer #2: This manuscript describes the results of an adaptive randomized trial of a harmful alcohol reduction intervention conducted in Tanzania. The trial has an interesting design, and some of the findings support the intervention. I do have some major concerns and clarifying questions about how the analysis was conducted and how results are currently reported.

Major concerns:
1. The primary outcome, as reported in the manuscript, differs from the pre-specified primary outcome in the protocol. The protocol specifies that DrInC (here presented as a secondary outcome that was not statistically significant) is the primary outcome, and specifies binge drinking as one of a few secondary outcomes. Was a protocol revision made prior to unblinding that changed the primary outcome? If not, this manuscript needs to transparently report the original primary outcome and secondary outcomes and not switch them.
R: Thank you for your comment and for noting this mistake on our behalf during the submission of this paper to Plos Medicine. In this submission an older version of the protocol (dated 2019) was included as supplementary material. This version did not include changes made to the protocol prior to the implementation of the trial. We ask reviewers to reference the updated protocol attached. Our initial protocol published in clinicaltrials.gov indeed listed the "change in alcohol-related harms measured by the Drinker Inventory of Consequences (DrInC)" as the primary outcome (published on 2020-08-27, https://clinicaltrials.gov/study/NCT04535011?tab=history&a=1#version-content-panel). The change in the primary outcome in favor of the "change in number of binge drinking days in the previous 4 weeks" was published in the clinicaltrials.gov website on 2020-12-07 (https://clinicaltrials.gov/study/NCT04535011?tab=history&a=2#version-content-panel). Similarly, the protocol paper also mentions the binge drinking days outcome https://trialsjournal.biomedcentral.com/articles/10.1186/s13063-022-06060-y. The changes in the protocol were initiated prior to starting enrollment based on feedback from our Advisory Board and documentation was completed (trial and clinicaltrials.gov) after final approval by our Data Safety Monitoring Board and all relevant regulatory bodies, prior to any participant enrollment.

2. Outcomes are self-reported. Authors indicate in the discussion that this could be subject to social desirability bias, but that these biases would balance across arms. I don't necessarily agree. Participants were not blinded to the intervention they received, correct? So those who received interventions related to alcohol harm reduction might be more likely to report less harmful alcohol behavior, particularly in a personal interview setting. If this type of differential social desirability in reporting occurred, the intervention effect would be arbitrarily inflated.
R: Thank you for this feedback. We agree that there is no way to blind our patients to the intervention, just our outcome assessors and our trial managers. And similarly, social desirability bias might be more prevalent when participants are unblinded to the intervention. In line with reviewer #1's feedback, we believe self-report bias was not a main driver of the differences we observed since other self-report measures did not yield differences between groups, thus providing evidence that the change in drinking behavior might be influenced by the intervention. Thus, we agree this is a common limitation in alcohol use reduction trials and have expanded on our limitation section (lines 483-516).

3. I have several concerns about the analysis conducted and how results are reported.
a. The authors need to clearly state which analyses were prespecified and which were not. For example, the protocol does not specify the use of zero inflated modeling. Was this prespecified in the SAP (which wasn't included) or changed after analysis began? Were the covariates included in models pre-specified somewhere?

R: Thank you for your comment. We apologize for the mistake of not including the Statistical Analysis Plan (SAP) in the original submission. The SAP included in the resubmission outlines the use of count-based models such as Poisson or negative binomial regression, as stated in the protocol paper (https://doi.org/10.1186/s13063-022-06060-y). Based on the observed distribution of the outcome data—which exhibited both overdispersion and an excess of zeros—we used a zero-inflated negative binomial (ZINB) model. While the use of a zero-inflated model was not explicitly stated in the protocol, this decision was made prior to conducting outcome analyses, included in our updated SAP and was reviewed and approved by the study's Advisory Board and Data Safety Monitoring Board. To improve the interpretability of the sensitivity analyses, we added a new table to the Supplementary Material (Table S4) that compares adjusted and unadjusted models. The results are consistent across complete case and imputed datasets, as well as between adjusted and unadjusted models. In all cases, the effect estimates and their confidence intervals remain within the confidence range, suggesting the findings are robust to both missing data handling and covariate adjustment.

b. The authors pre-specified in the protocol that they would conduct multiple imputation for missing data, and they did not. There is a substantial amount of missing data in both arms (29% usual care, 32% intervention arms), so complete case analysis alone is not sufficient. For all complete case analyses, results need to be caveated by the n, % of missing data, which they currently don't report clearly (it can be gleaned from Figure 1). But consistent with the protocol, the primary analysis should include MI.

R: Thank you for raising this important point. We apologize for not including the multiple imputation (MI) analysis in the original submission, despite it being pre-specified in the protocol. Our missigness was deemed to be at random and we initially chose to report the complete case analysis of this Stage I report, leaving the full analysis for when Stage II was completed. However, we agree with the importance of reporting the MI results and have included them in this report.

We also acknowledge the importance of clearly reporting the extent of missing data. While Figure 1 includes this information, we have revised the manuscript to make it more explicit in the text (310-322). In response to your comment, we report the sensitivity analysis using multiple imputation, with full details included in both the main text and Supplementary Table S4. The results of the imputed models were consistent with those from the complete case analyses, supporting the robustness of our findings.

Importantly, Figure 1 describes the sample of patients used for the main analysis displayed in the original submission. Overall, we have made changes to the Methods and Results sections, and the imputation analysis has been added to ensure consistency with the protocol and strengthen the validity of our conclusions.

c. The analysis methods seem unnecessarily complicated (and introduce unnecessary parametric assumptions). For each outcome, the authors could just compute the change in scores or counts for participants from baseline to the end of study and conduct a nonparametric test for these change scores (e.g., Wilcoxon rank sum test). Corresponding estimates of median difference between arms with 95% CIs can be reported. I recommend this be conducted as a sensitivity analysis, as the inclusion of so many baseline covariates and parametric assumptions make the current results difficult to interpret.

R: Thank you for this suggestion. As suggested, we conducted a Wilcoxon rank-sum test comparing change scores in binge drinking days between baseline and 3-month follow-up. The results confirmed the difference observed between groups ($p < 0.05$). However, we had defined to conduct a more robust model that would allow us to estimate the more accurate estimate adjusting for baseline confounders. We opted to keep the reports defined in our analysis protocol and SAP. We can report additional analysis on the same data but opted not to report the Wilcoxon test and other simpler models to be consistent with our analytical approach defined in our protocol. We believe that, given the data distribution, the approach we report addresses the data distribution and allows us to estimate group-level differences in expected binge drinking days over time.

d. For each model, authors need to say what covariates were included and how the "predicted means" were computed. For zero-inflated models, there is a model for both the probability of being an excess zero and a model for the mean count given that one is not an excess zero. Covariates in each need to be described. How was time incorporated in each longitudinal model (e.g., as a linear term)? In general, authors should say what variables were included in each model and how overall estimates were obtained from the models. A detailed statistical appendix would improve clarity/interpretability of all analyses.

R: Covariates and Model Specification: We report a series of sensitivity analysis in our supplementary Table S4, including adjusted and unadjusted models. These models control for age, tribe, self-reported sex,

employment status, monthly personal income, and monthly household income. The results from the adjusted models are consistent with the unadjusted findings.
For the primary analysis, we used a zero-inflated negative binomial model, implemented using the glmmTMB package in R. The count part of the model included fixed effects for treatment arm (intervention vs. usual care), time (baseline vs. 3-month follow-up), and their interaction, along with a random intercept to account for clustering by participant ID. The zero-inflation part of the model included the treatment arm as a predictor of the probability of being in the excess-zero group. This model allows us to estimate both the average number of binge drinking days among participants who drink (count model) and the probability that a participant reports zero days due to structural reasons (zero-inflation model). Predicted means were calculated by combining estimates from both parts of the model and averaging over the sample, accounting for the random effects structure.
Time modeling: Time (variable name = period) was incorporated into all longitudinal models as a binary variable indicating baseline or 3-month follow-up. This allowed us to estimate the change in outcomes over time and test for differences in change between the intervention and usual care arms (variable name = treat) via an interaction term (period * treat).
Predicted means: Predicted means and marginal effects were calculated using post-estimation simulation from the fitted models. For the zero-inflated negative binomial models, this involved combining results from both the count and zero-inflation components to estimate expected binge drinking days for each individual. These individual-level estimates were then averaged to obtain predicted means by study arm and time point.

e. It is not appropriate to conduct statistical testing for baseline/Table 1 variables.
R: Thank you for pointing this out. Our original Table 1 did not include p-values, but it mistakenly included a footnote indicating that a statistical test had been conducted in a prior draft as a previous reviewer request. We apologize for this oversight and have revised the table to remove that annotation. The updated version now correctly presents baseline characteristics without any reference to statistical testing.

4. The sample size calculations differ from the protocol, as those were based on a different primary outcome. Which was used to power the study? Authors also need to say what statistical test was used to determine power and exactly what sample size was needed according to the calculations.
R: Thank you for this comment. We apologize for the confusion, which stemmed from referencing an outdated version of the protocol in the original submission. We have now included the correct version of the protocol (dated 16 June 2023) in the updated supplementary material. As described in the revised protocol, the sample size calculation for Stage 1 was based on the updated primary outcome: the number of binge drinking days per month. We further clarified this on the paper.

Other comments:
1. This manuscript pools together two intervention arms. This is prespecified in the protocol and part of the adaptive design, but this pooling will likely confuse readers. Authors should explain why this was done early on to avoid confusion.
R: Thank you for this feedback. We have described why we pooled our intervention arms in a new paragraph in the *Study Design* section of the methods (lines 147-156).

2. The incidence rate reported in the second sentence of the introduction needs to be in terms of some unit of time, for it to be a rate.
R: This reference was corrected as suggested (lines 75-77).

3. Authors talk about using the bootstrap to "estimate the confidence intervals". We only estimate parameters, and confidence intervals are not parameters. Instead, they could say "compute" or "construct".
R: The term “estimate” was replaced in the methods section (lines 302-304).

4. Table 2 would be more interpretable if mean/median differences in change scores between arms were included, with 95% CIs. This would parallel nicely with the sensitivity analysis I recommended above.
R: Thank you for this suggestion. We agree that presenting mean or median differences in change scores between arms can be helpful for interpretation. However, given our predefined analysis approach, we believe that reporting these values on this table could mislead the interpretation of the analysis results. We included this table to report the observed data, but given its highly dispersion and zero influence, opted to draw the

reader's attention to the predicted means that also contains the 95% CIs, a common reporting practice for ZINB models. However, we are willing to make this change if the reviewer believes it will not deviate the reader from the primary prespecified analysis.

5. Figure 2 is overly complicated. The bars just indicate where the means are and could be removed. A more meaningful figure might depict individual level data, like via a scatterplot in the background of the bars.
R: Thank you for your suggestion regarding Figure 2. We appreciate the idea of incorporating individual-level data into the visualization. However, we believe that the current version of the figure effectively communicates the group-level trends over time in a clear and interpretable way. We opted for this simplistic representation of individual data to facilitate the comprehension of the analytical approach. The bars are intended to provide a visual reference for the mean values, complementing the modeled estimates and confidence intervals. Given the skewed distribution and large proportion of zeros in the data, plotting individual points would likely result in a dense and less interpretable figure. For these reasons, we have opted to retain the current format of Figure 2. We are happy to change this visualization if the reviewer believes it will be better represented.

6. In the limitations, authors refer to "a regression to the mean" but don't explain what they mean by this or cite other literature for the "As in many alcohol trials" claim.
R: Thank you for this helpful comment. We agree that the phrase “regression to the mean” was used imprecisely and may have caused confusion. We have revised the limitations section to clarify our intent. Specifically, we now explain that the observed reductions in alcohol use across both arms are likely influenced by contextual factors rather than statistical regression to the mean. This pattern is common in alcohol intervention trials, especially those conducted in emergency care settings, where many individuals report little or no alcohol use at follow-up—regardless of intervention—due to factors such as trauma exposure or the acute care experience. We have removed the term "regression to the mean" and replaced it with a more accurate description of this phenomenon, along with appropriate context (lines 518-520).

Reviewer #3 Comments

Reviewer #3: This article reports the results of a superiority clinical trial comparing the effectiveness of a culturally adapted brief intervention with mobile health-based boosters to usual care. Participants were adults presenting to an Emergency Department in Tanzania for an acute injury and either reporting alcohol use prior to the injury, Alcohol Use Disorder Identification Test ≥8, and/or having positive BAC according to breathalyzer measures. Participants were randomly assigned to 2 intervention conditions (ED brief intervention + personalized mobile booster, and ED brief intervention + standard mobile booster) or usual care, with a 1:1:1 allocation ratio. The authors pooled the 2 intervention arms and compared them to usual care to determine the effectiveness of the intervention in reducing alcohol use and consequences at 3-month follow-up. Analyses showed statistically significant differences for 3 alcohol use measures, but no significant differences were found between the two groups in terms of drinking-related consequences or depression.

The topic is of interest and testing the effects of a culturally adapted intervention approach is both timely and well-founded. Nevertheless, there are several methodological concerns and lack of design description resulting in skepticism about finding reliability and relevance. These major concerns are described below, followed by minor points listed in order of appearance in the text.

First of all, it is not clear why the design have 3 arms, 2 of which are pooled in the analysis. I think I understand after having read the study protocol, but neither the introduction nor the methods section clearly explains this complicated design. As such, the study design looks wrong (one should not combine 2 groups when randomization is conducted 1:1:1, at least not as the primary analysis).
R: Thank you for this feedback. We have further described the study design, the adaptive processes of this trial, Stage 1’s research question and how subsequent stages would be using stage 1 data for their analysis, thus describing why the allocation and combining intervention groups (lines 147-156).

According to the study protocol, other endpoints were collected (6, 9, 12 and 24 months), but this paper only report 3-month outcomes. The publication strategy is not clear and should be described in the introduction. Without a more detailed explanation, it might look like a "salami slicing" strategy.

R: Thank you for this feedback. This paper is a report of the first Stage of this Pragmatic Adaptive Trial. We are reporting only our outcomes at our primary endpoint of 3 months in this manuscript as the primary analysis for the first Stage I, while Stage II is ongoing. We believe that a larger paper with both Stages results would not have enough emphasis on the primary analysis, and space to properly report on the decision that guides our assumption that the intervention is efficacious in this setting. We are doing this because 1) we want to obtain all follow up in order to report all long-term outcomes possible for this stage 2) we anticipate these effects to be less than the 3-month outcomes. Our reasoning is, a reduction of 2 binge drinking events per month at 3 months is effective enough to proceed planning for implementation in a context where no other treatments currently exist. Further data on which, or if, a text-based booster should be implemented, and the length of time the reduction is seen from these intervention arms will be included in future manuscripts; these data speak to how (not if) this intervention should be implemented and possible follow up needs for longer-term treatment impact.

The choice of the primary outcome is questionable. The median is 0 at baseline and the 25th and 75th percentile are 0 at follow-up. The standard deviations also indicate a strongly skewed distribution. Why using such a rare behavior as the main outcome? It also raises the question of participants' selection. Were the inclusion criteria too wide?
R: Thank you for this feedback. Binge drinking is a relatively rare event but with significant risks associated with it. After suffering an injury, there is also a known process where people reduce their alcohol use behavior, called "a regression to the mean" in the alcohol literature. This is seen globally and can be due to usual changes in alcohol use or by 'new normals' after an injury and change in behavior due to an injury. We had extensive discussions with our Advisory Board, who are experts in adaptive trials, and alcohol trials. We decided that binge drinking days are the best outcome because overall it is a marker of highest risk of alcohol related harms and repeat injury or emergency department visits. Alternatively, quantity of alcohol use, could be one drink daily which carries far lesser injury and treatment seeking risk.

As for participant selection, it is possible that our inclusion criteria were too wide. We are pragmatically implementing this intervention in order to understand its impact and then will be conducting further moderator and mediator analyses to understand if our population can or should be narrowed. As there are no other alcohol harm reduction interventions available for this population, narrowing the sample size might improve the impact but would reduce generalizability; overall, we are interested in finding effectiveness data for a pragmatic intervention generalizable for the region.

Related to this, the main results are complicated to understand. Looking at medians, there were no changes across time among both groups (0 to 0 in both groups). Looking at means, there were important decreases in both groups (2.2 to 0.8 in the control group and 3.0 to 0.4 in the intervention group). Looking at regression models, there is a decrease of 3.4 (95% CI -4.8; -2.3). How is this possible? How is such a 95% CI possible with both medians at 0? An explanation is that most participants did not change (since they could not decrease as they did not binge…), but a small group did decrease and draws all the effect. This should be better described (and maybe tested in secondary analyses). The findings in the abstract should also be revised since currently only the important effect of the regression model is reported.
R: As noted, the median number of binge drinking days remained 0 at both baseline and 3-month follow-up in both arms, indicating that more than half of participants reported zero binge drinking days at both time points. In contrast, the mean values suggest notable reductions (from 2.2 to 0.8 in the usual care group and from 3.0 to 0.4 in the intervention group). Indeed, we expected this behavior in the data given the rarity of the event, aligned with the reviewers previous comment. We had anticipated this behavior in our analytical strategy, defining a prior the use of count models to address this nuance in change on count vs. continuous data. This difference arises due to the highly skewed distribution of the outcome, with a large proportion of participants reporting no binge drinking. To improve interpretability, we report predicted means for each group at each time point, which were derived from the fitted zero-inflated negative binomial model. These predicted means reflect the expected number of binge drinking days, accounting for both the probability of excess zeros and the count of days among those who do drink. The predicted values are based on marginal effects, averaging over the random effects structure. To estimate the difference in predicted means between groups and time points—and to construct confidence intervals around these differences—we used non-parametric bootstrapping with 1,000 resamples. For each bootstrap sample, we re-fitted the model and re-estimated the predicted means and

contrasts of interest. The 95% confidence intervals for the differences were then calculated using the percentile method, capturing the uncertainty associated with both the outcome distribution and model estimation. This approach allows us to present robust, model-based estimates that account for the skewness and excess zeros in the data, while providing empirically derived confidence intervals that do not rely on asymptotic assumptions.

MINOR POINTS

Abstract
The three conditions should be better described in the abstract.
R: Thank you these changes were made (lines 40-50).

Authors should indicate participation rate (448 over how many eligible?) in the Findings section.
R: Thank you, these changes were made (line 55).

The sentence reporting the main effect is not correct. The difference reported is the difference in difference and not the reduction among the intervention group per se. See also, my comment above about the findings being reported.
R: Thank you for your comment. We have revised the sentence in the abstract to clearly indicate that the observed reduction of 2 binge drinking days reflects a difference-in-differences estimate comparing the intervention group to the usual care group (lines 59-61).

Introduction
"Alcohol use is a significant global health crisis". This sentence should be rephrased.
R: Thank you, these changes were made (lines 74-76).

Authors should add a reference when describing BI as incorporating MI.
R: Thank you, these changes were made (lines 103-104.

The literature on BI in the ED is restricted to a few studies. There are several other studies, as well as literature reviews on this topic. There are also other studies testing the effect of models conducted by addiction liaison staff. And other studies testing the effects of booster sessions.
R: Additional references, including review articles, have been added to the manuscript (lines 103-108).

Methods
How was the binge drinking days outcome measured? Timeline follow-back procedure? Or a single self-report question? Same question for number of standard drinks. It would be difficult to remember number of drinks over the last 30 days without a TLFB procedure.
R: Thank you for this question. Yes, a timeline follow-back procedure was used for all binge drinking days, drinking days, and quantity of standard drinks. This information was clarified in the methods (261-266).

Consider renaming "alcohol use behavior" as measured by the AUDIT. Either keep "AUDIT score" or state something like "AUD severity".
R: Thank you, these changes were made throughout.

The sample size estimation is poorly presented in the methods. Additional information should be provided to justify the expected size of effect and the corresponding numbers.
R: Thank you for this comment. We have revised the Methods section to provide a clearer and more detailed explanation of the sample size estimation (lines 271-283). The sample size was calculated to detect a 35% greater reduction in the number of binge drinking days per month in the intervention arm compared to the control arm. This effect size was based on prior alcohol intervention trials conducted in acute care settings, which have demonstrated treatment effects ranging from 20% to 40%. Assuming a baseline mean of four binge drinking days per month and a standard deviation of 3.4, a 35% reduction translates to an absolute difference of approximately 1.2 days between groups. Assuming 80% power and a two-sided alpha of 0.05, we estimated that 164 participants per arm would be needed. This trial was designed with three planned interim analyses

following an O'Brien-Fleming alpha-spending approach to control for type I error. The current paper reports on data collected at the second planned interim analysis, after approximately two-thirds of the total sample size had been reached. The significance level for this analysis was adjusted according to the O'Brien-Fleming boundary.

Why did the authors use different approaches for drinking and non-drinking outcomes. A baseline adjustment approach would have been possible for AUDIT, DRINC, and PHQ outcomes. Why using mixed models here and not for drinking outcomes? In the protocol, baseline adjusted approach was proposed for all analyses.
R: Thank you for your comment. We used a longitudinal constrained approach for all outcomes, including drinking and non-drinking outcomes. For the AUDIT, DrInC, and PHQ-9 outcomes, we used linear mixed-effects models to assess changes over time. This decision was based on the continuous nature of these outcomes. In contrast, drinking outcomes (such as number of drinking days, binge drinking days, and drinking amount) are highly skewed count variables with many zeros, which are not well modeled using linear models. For these outcomes, we used generalized linear mixed models (GLMMs) with a zero-inflated negative binomial distribution to appropriately account for overdispersion and excess zeros. Covariate-adjusted versions of all models are included in the supplementary materials (Table S4), and results were consistent with the unadjusted models. We have clarified these modeling decisions and their rationale in the Methods section to improve transparency and alignment with the protocol (291-300).

Results
The first sentence of the Results is unclear. Authors did conduct a second interim look for 2 years and a half? This sentence should be rephrased and the use of interim analyses and stopping rules should be presented in the methods (and maybe introduced in the introduction).
R: Thank you for this observation. We have rephrased the first sentence to clarify that the second interim analysis was conducted in April 2023 (like 326-328). Also, we have added information on how interim analyses were planned in the methods section (276-283).

The patient flow should be more detailed. What were the causes of exclusion of the 1032 not included?
R: The 1032 did not meet any one of the alcohol screening criteria (self-report of alcohol use, AUDIT greater than or equal to 8, or breathalyzer positive). This was added in the patient flow discussion (328-331).

What are the "Other" reasons for not completing follow-up? How come that staff did not attempt to contact 4 participants in the intervention group?
R: Thank you for your question. We agree that the “Other” category was not sufficiently clear in describing the reasons participants were lost to follow-up. We reviewed the study records and worked closely with the study staff to reclassify these cases more accurately based on the available documentation. A revised version of Figure 1 is now included, clearly presenting the updated and specific reasons for loss to follow-up.
Regarding the participants who were not contacted for follow-up, this occurred during the first year of the study, when the number of follow-up visits increased (i.e., at 3, 6, 9, and 12 months). In a few cases, follow-up attempts were unintentionally missed due to study staff error. Once this issue was identified, we clarified the follow-up procedures, conducted additional staff training, and strengthened documentation and monitoring processes to ensure consistent follow-up in subsequent phases of the study.

Discussion was not reviewed since there were too many issues in the introduction and methods, preventing reliable and interpretable findings.

<u>Reviewer #4 Comments</u>

Reviewer #4: C1. Thank you for the opportunity to review this manuscript evaluating the effectiveness of brief intervention plus text-based messages to reduce harmful and hazardous alcohol use in the emergency department. Many aspects of this work need to be clarified to enhance the reporting of both methods and results and to enhance the overall relevance of this work to the journal's readers.
For example, it is unclear if recruited participants in the emergency department worked with one nurse or multiple nurses for the entirety of the brief intervention and SMS Booster messages. If multiple, how can authors ensure the fidelity of the intervention?

R: Thank you for this excellent question. For our pragmatic implementation, we trained multiple Tanzanian nurses to conduct this intervention. Information on our intervention fidelity program is included now in the manuscript.

C2. The lack of details on training the nurses and research assistants to ensure the delivery of a standardized intervention is unclear.

R: Thank you for this excellent question. For our pragmatic implementation, we trained multiple Tanzanian nurses to conduct this intervention. Information on our intervention fidelity program is included now in the manuscript (lines 115-118).

C3. The authors need to discuss in detail the possible response bias introduced by having unblinded research nurses open the SNOSE to determine group assignment, conduct the recruitment, and implement the intervention.

R: We are sorry for this error, all enrollment and recruitment was done by blinded research assistants. Opening the allocation envelopes was performed by unblinded clinical nurses (who would do the intervention if assigned), and follow up was performed by blinded research assistants. This is further described in the blinding processes (lines 197-204).

C4. There are considerable concerns given the reliance on self-reporting for primary and secondary outcomes. For example, the standardized AUDIT questions assess alcohol use within one year, and it is not appropriate to change the questions' timeframe to a three-month time window. The authors also need to explain why they used the number of binge drinking days as the primary outcome instead of alcohol consumption in grams per week. The authors need to check the core outcome set (COS) recommended by the International Network on Brief Interventions for Alcohol and Other Drugs (INEBRIA) organization. The COS covers domains of average alcohol consumption (e.g., alcohol consumption in grams per week), frequency of alcohol consumption (e.g., frequency of drinking in past 30-day), and episodes of hazardous or harmful drinking (e.g., episodes of binge drinking and heavy drinking in past 30-day). The COS followed the Core Outcome Measures in Effectiveness Trials (COMET) guidelines and recommended the evaluation of the effectiveness of alcohol trials in all settings.

R: Thank you for this feedback. There are no pragmatic alternatives to self-reported outcomes in our Tanzania, and low resource settings. We have further described why our primary outcomes were determined to be binge drinking days. This project was funded in January 2020 prior to the publication of the COS/COMET guidelines; that being said, as a primary outcome, we are using INEBRIA's COS recommended outcomes of frequency of heavy episodic drinking determined by a timeline follow back process over the prior 28 days. We did not use AUDIT #6 because we believe that asking one question was less specific than our process of conducting an interview using a TLFB process to determine the amount of alcohol in standard drinks and identify binge drinking days.

C5. The sample size estimation needed to be written in detail and the calculation did not account for the possible retention rate at the follow-up and the allocation ratio.

R: Thank you for this comment. We have made changes to the methods section to include a more clear description of the sample size calculation performed in our trial. In detail, the trial was powered to detect a 35% difference in reduction between the intervention and control arms. Assuming an average of 4 binge drinking days per month (standard deviation = 3.4), this corresponds to an expected difference of approximately 1.2 days between groups. The sample size calculation of 164 patients was deemed needed to reach 80% power and a 5% significance level. Accounting for an anticipated retention rate of 80%, we estimated that 205 participants per arm would be enrolled (lines 270-283).

C6. The authors stated that the primary outcome analysis was based on intention-to-treat. However, the number of participants included for primary outcome analysis in the Figure 1 flowchart did not say so.

R: We have reviewed Figure 1 to improve clarity.

C7. The authors did not perform sensitivity analyses (a requirement of the CONSORT guideline for RCT study) to handle the missing data.

R: Thank you for raising this important point. We apologize for not including the multiple imputation (MI) analysis in the original submission, despite it being pre-specified in the protocol. In response, we have now added details in both the main text (lines 310-323) and the supplementary material describing a sensitivity

analysis using multiple imputation (Table S4). Multiple imputation was conducted using the mice package in R. We used Predictive Mean Matching (PMM) to impute missing values for continuous variables. PMM is a semi-parametric method that preserves the distribution of observed data by selecting real, observed values from similar cases, rather than generating synthetic values. This approach ensures that imputed values remain realistic and within the observed range. We generated 10 imputed datasets (m = 10); the imputation process was run for up to (maxit=50) 50 iterations with 100 burn-in iterations, and a random seed was set for reproducibility. The results of the MI analysis are consistent with the complete case analysis.

C8. It is unclear from the results if there was a dose-response between the number of SMS messages a participant read and reductions in the number of binge drinking days, standard drinks, and drinking days.
R: Given the pragmatic nature of our study, we are not able to identify if a participant read a text message. We will further evaluate the effectiveness of the text based booster in Stage 2 of our adaptive trial, as such this is outside of the scope of this project.

C9. The authors need to state the theoretical framework that guided the design of the SMS booster messages to increase the study's transferability.
R: The creation and feasibility of the SMS booster messages is fully described in another manuscript cited in this manuscript and is outside of the scope of this manuscript. The citation for this information was added to the manuscript.

C10. The authors did not mention any details on the usual care practice.
R: Data on the usual care practice are included in the description of the arms. Currently, standard practice at KCMC for injury patients does not include screening or testing for alcohol use, alcohol use disorder, or its consequences, nor education on the consequences of alcohol use (233-237).

C11. The authors stated that this is a pragmatic RCT. It is unclear which components of this study's design were pragmatic in nature. Clarification is needed, including using the CONSORT extension to report on pragmatic trials and enhance transparency.
R: We adopted a pragmatic goal in our trial development and applied it to all our decisions made during the trial development and implementation. Using the PRECIS-2 tool, these components included a typical patient population with minimal inclusion criteria, bedside recruitment during already occurring ED visits, a pragmatic setting occurring in an ED where the results will be applied, aligned the training with clinical care and expertise so there is minimal change to clinical delivery after the trial is complete. Given the prior literature in brief interventions and intervention fidelity, we choose to have a more rigid intervention delivery and adherence standards applied to the process but in an easily implementable and contextually relevant way. We also had few to no in -person follow-ups for the study, attempting to keep the care as similar to usual care as possible and analyzed the data in an intention to treat analysis. This information was added to the study design section (158-162).

C12. The authors did not exclude the potential participants with psychiatric disorders or participating in other alcohol prevention and treatment programme. For example, there is a bi-directional association between alcohol consumption and mental illness that might considerably affect the study outcomes.
R: Thank you for this excellent observation. We are very aware there is an overlap between psychiatric disorder and alcohol use. Our pragmatic approach to screening and treatment being conducted in an emergency department supported our focus on how this intervention can be effective when applied in a broad and pragmatic fashion regardless of underlying or overlapping psychiatric disorder. Given our methods, we anticipate that the impact of this intervention to be UNDERestimated given our pragmatic approach. This clarification was added to the interpretation and manuscript.

C13. The authors did not mention how to handle the potential confounders when performing data analyses in the methodology nor include them in the footnote of the tables. Although it is an RCT study, the confounder still need to be adjusted. Please check the reference below.
Holmberg MJ, Andersen LW. Adjustment for Baseline Characteristics in Randomized Clinical Trials. JAMA. 2022 Dec 6;328(21):2155-2156.
R: The main models presented in the manuscript are indeed unadjusted and do not include any covariates. In response to reviewer feedback, we conducted additional adjusted analyses, now included in Supplementary

Table S4. These models control for age, tribe, self-reported sex, employment status, monthly personal income, and monthly household income. The results from the adjusted models are consistent with the unadjusted findings—effect estimates remain statistically significant and fall within the confidence intervals of the original models, supporting the applicability of the original results presented.

C14. The author did not mention that any incentive would be given to the participants who completed the follow-up. Details on compensation or what was driving intervention engagement need to be thoroughly discussed and assessed.
R: Thank you for this excellent comment and we are sorry for the oversight. It is now documented that participants received 5000 Tanzanian Shillings or approximately US$2.00 by MPesa, or a phone managed money system, for each follow-up performed on phone (lines 266-268).
